# Assessment of Stability and Discrimination Capacity of Radiomic Features on Apparent Diffusion Coefficient Images

**DOI:** 10.1007/s10278-018-0092-9

**Published:** 2018-05-03

**Authors:** Marco Bologna, Valentina D. A. Corino, Eros Montin, Antonella Messina, Giuseppina Calareso, Francesca G. Greco, Silvana Sdao, Luca T. Mainardi

**Affiliations:** 1 Departement of Electronics, Information and Bioengineering, Milan, Italy; 2 Fondazione IRCCS Istituto Nazionale dei Tumori, Milan, Italy

**Keywords:** Apparent diffusion coefficient maps, Radiomics, Radiomic features stability, Magnetic resonance imaging, Intra-class correlation coefficient

## Abstract

The objectives of the study are to develop a new way to assess stability and discrimination capacity of radiomic features without the need of test-retest or multiple delineations and to use information obtained to perform a preliminary feature selection. Apparent diffusion coefficient (ADC) maps were computed from diffusion-weighted magnetic resonance images (DW-MRI) of two groups of patients: 18 with soft tissue sarcomas (STS) and 18 with oropharyngeal cancers (OPC). Sixty-nine radiomic features were computed, using three different histogram discretizations (16, 32, and 64 bins). Geometrical transformations (translations) of increasing entity were applied to the regions of interest (ROIs), and the intra-class correlation coefficient (ICC) was used to compare the features computed on the original and modified ROIs. The distribution of ICC values for minimal and maximal entity translations (ICC_10_ and ICC_100_, respectively) was used to adjust thresholds of ICC (ICC_min_ and ICC_max_) used to discriminate between good, unstable (ICC_10_ < ICC_min_), and non-discriminative features (ICC_100_ > ICC_max_). Fifty-four and 59 radiomic features passed the stability-based selection for all the three histogram discretizations for the OPC and STS datasets, respectively. The excluded features were similar across the different histogram discretizations (Jaccard’s index 0.77 ± 0.13 and 0.9 ± 0.1 for OPC and STS, respectively) but different between datasets (Jaccard’s index 0.19 ± 0.02). The results suggest that the observed radiomic features are mainly stable and discriminative, but the stability depends on the region of the body under observation. The method provides a way to assess stability without the need of test-retest or multiple delineations.

**Electronic supplementary material:**

The online version of this article (10.1007/s10278-018-0092-9) contains supplementary material, which is available to authorized users.

## Introduction

Radiomics is an emerging field in quantitative imaging that uses image features to objectively and quantitatively describe tumor phenotypes [1]. The underlying hypothesis of radiomics is that such features could capture information not currently available using simple radiological analysis [2]. Radiomic features are non-invasively obtained on images that are part of the process of tumor evaluation and treatment, such as computed tomography (CT), magnetic resonance imaging (MRI), and positron emission tomography (PET). Thus, radiomic analysis could be performed without the need of further specific exams. Moreover, traditional histological analysis based on tissue samples, obtained through biopsies, cannot capture the heterogeneity of the whole tumor. On the other hand, radiomics, analyzing the entire tumor, can provide a complete and quantitative description of tumor heterogeneity, which may have profound implications for drug therapy in cancer [3]. All of the previous advantages make radiomics a technique of interest for tumor characterization. As a matter of fact, radiomics has already found a wide range of possible applications [4–14] such as prediction of clinical outcomes and response to treatment, tumor staging, discrimination of different types of tumor tissues, and assessment of cancer genetics.

The number of features used in radiomic studies may range from just a few [15] to several hundred [6]. However, not all the hundreds of extracted features bring information: some may be irrelevant or unreliable for the clinical question of interest. A process of feature selection is therefore necessary.

Stability analysis, assessing the robustness of the features, is a preliminary step in the process of feature selection [6, 12, 16]. Radiomic features stability can be investigated in several ways: (1) test-retest [6, 12, 16–23]; (2) multiple delineations of the region of interest (ROI) representing the tumor [6, 18, 21]; (3) change in image reconstruction and automatic segmentation parameters in PET or CT studies [20–22, 24, 25]; (4) change in image acquisition techniques [20, 24]; (5) inter-machine reproducibility [20, 26]. The most common techniques that are used for preliminary feature selection are typically the first two [6, 12, 16]. However, there are several problems concerning the different types of stability analysis. Different acquisitions are required to perform a proper test-retest analysis and the same thing can be said for analyzing stability to acquisition parameters and inter-machine reproducibility. Such requirements make the implementation of those types of analyses in the clinical routine. The analysis of stability to multiple delineations does not need multiple image acquisition, but drawing multiple ROIs on the same set of images can be very time-consuming. To solve the latter problem, alternative approaches may be considered. For example, in [12], stability is assessed through small geometrical transformations of the ROIs, which are used to mimic multiple manual delineations. In [27], the stability analysis is performed by comparing radiomic features computed on the entire ROI, and on a “digital biopsy,” i.e., a small portion of the ROI that is large enough to capture the heterogeneity of the tumor. Last, comparison of radiomic features obtained with multiple initialization of a semi-automatic segmentation algorithm or with different segmentation algorithms (like in [28]) could potentially be used for stability assessment. Although these approaches strongly reduce the amount of manual work necessary for a stability analysis of the radiomic features, they cannot be used to evaluate the discriminative power.

In the current study, we perform an analysis similar to the one presented in [12], so that stability of radiomic features could be evaluated starting with just one acquisition and one ROI. In addition to ROI transformations that are small and thus can mimic errors due to manual delineation, we apply also large geometrical transformations to evaluate features discrimination capacity. Our hypothesis is that features that do not change their values for large transformations are irrelevant and should therefore be excluded.

In this study, diffusion-weighted MRI (DW-MRI) of two different tumor types (oropharyngeal cancers and soft tissue sarcomas) are analyzed. DW-MRI have been chosen because they can be used to compute maps of apparent diffusion coefficient (ADC), which have been shown to be very useful for tumor detection and characterization [11, 29, 30], evaluation of treatment response [5, 31], and tumor staging [8, 32]. Also, unlike other types of MRI, ADC maps have been shown to be useful to assess tumor cellularity, even across different scanners [33], provided that the same range of *b* values and the same field strength are used [34, 35].

The aim of the present study is to provide a method to perform a preliminary feature selection based on features stability. An innovative characteristic of the method is that it does not require either multiple acquisitions or multiple manual delineations.

## Material and Methods

### Study Population

In this study, two different datasets were retrospectively analyzed: the first one contains DW-MRI images of soft tissue sarcomas (STS); the second one contains DW-MRI images of oropharyngeal cancer (OPC). The two datasets are provided by the Fondazione IRCCS Istituto Nazionale dei Tumori (Milan, Italy).

Both datasets consisted of 18 patients who underwent an MRI acquisition before starting the treatment. Both studies were approved by the ethical committee of Fondazione IRCCS Istituto Nazionale dei Tumori (Milan, Italy) and conducted in accordance with the Helsinki Declaration; all patients gave their written informed consent. All patients’ data were anonymized prior to the analysis.

### Image Acquisition

#### STS Dataset

DW-MRI images were acquired using Achieva 1.5 T system (Philips Medical system, Eindhoven, Netherlands)—5 patients—or a Magnetom Avanto 1.5 T system (Siemens Medical Solutions, Erlangen, Germany)—13 patients—both with a body-matrix coil and spine array coil for signal reception. The data were acquired axially by means of echo planar imaging. The sequences’ parameters (for both equipment) are reported in Table 1. Diffusion-weighted images (DWI) were acquired using four *b* values (50, 400, 800, and 1000 s/mm^2^).

**Table 1:** MRI sequence parameters by MRI scanners

Sequence parameter	STS database		OPC database
	Siemens Avanto (*n* = 13)	Philips Achieva (*n* = 5)	Siemens Avanto (*n* = 18)
Sequence name	ep2d	dwi_ssh	ep2d
Matrix (pixels)	192 × 192	255 × 255	132 × 132
Resolution (voxel/mm)	1.98 × 198	1.37 × 1.37	1.89 × 1.89
Field of view (mm)	380 × 380	350 × 350	250 × 250
Repetition time (msec)	5400	7410	3300
Echo time (msec)	78	63	64
Slice thickness (mm)	4 (no gap)	5 (no gap)	3 (gap 0.9)
Number of excitations	4	3	3

#### OPC Dataset

DWI were acquired using Magnetom Avanto 1.5 T system (Siemens Medical Solutions, Erlangen, Germany). The sequence parameters are reported in Table 1. DWI images were acquired using ten *b* values 0, 10, 20, 50, 70, 100, 150, 200, 500, and 1000 (s/mm^2^).

### Image Processing

For both the datasets, ADC maps were computed. The ADC was defined as the slope of the linear regression of the logarithm of the DWI exponential signal decay on the *b* values [36]. The calculation was performed pixel-wise using ITK 4.8 [3].

For the both datasets, the segmentation of the gross tumor volume (GTV) was performed by an expert radiologist on the DW-MRI computed with the lowest *b* value, where the tumor is the most visible. The preprocessing steps were performed using 3D Slicer [37].

### Radiomic Feature Extraction

In this study, 69 radiomic features were computed, pertaining to two main categories: (1) intensity-based and (2) texture-based. The complete list is reported in Table 2.

**Table 2:** Radiomic features analyzed in this study, divided by category

First-order statistics (FOS)		
-Signal energy -Signal kurtosis -Signal mean absolute deviation (MAD) -Signal maximum -Signal mean -Signal median -Signal minimum -Signal quantile 0.01 -Signal quantile 0.1 -Signal quantile 0.2 -Signal quantile 0.3 -Signal quantile 0.4 -Signal quantile 0.5 -Signal quantile 0.6 -Signal quantile 0.7	-Signal quantile 0.8 -Signal quantile 0.9 -Signal quantile 0.99 -Signal range -Signal root mean square (RMS) -Signal skewness -Signal standard deviation (SD) -Signal variance -Histogram entropy -Histogram kurtosis -Histogram mean absolute deviation (MAD) -Histogram maximum -Histogram mean	-Histogram median -Histogram minimum -Histogram range -Histogram root mean square (RMS) -Histogram skewness -Histogram standard deviation (SD) -Histogram variance -Histogram uniformity -Histogram total frequency
Gray-level co-occurrence matrix (GLCM)		
-Autocorrelation -Cluster prominence -Cluster shade -Cluster tendency -Contrast -Correlation -Difference entropy -Dissimilarity	-Energy -Entropy -Homogeneity -Homogeneity 2 -Information measure of correlation 1 (IMOC1) -Information measure of correlation 2 (IMOC2)	-Inverse difference moment -Inverse difference moment 2 -Inertia -Inverse variance -Max probability -Sum average -Sum entropy
Gray-level run length matrix (GLRLM)		
-Gray-level non-uniformity -High gray-level emphasis -Long run emphasis -Long run high gray-level emphasis	-Long run low gray-level emphasis -Low gray-level emphasis -Run length non-uniformity -Run percentage	-Short run emphasis -Short run high gray-level emphasis -Short run low gray-level emphasis

Features belonging to the intensity-based group (first-order statistics or FOS) included statistical information about the signal intensity and histogram distribution of the pixels in the ROI. The histogram was evaluated between 0 and 4000 *10^−6^ mm^2^/s using *N* bins. In this study, three values of *N* were tested (16, 32, and 64 bins) to evaluate whether the bin number affects the stability of the features.

Texture-based features were computed on the gray-level co-occurrence matrix (GLCM) [38] and the gray-level run length matrix (GLRLM) [39]. For a given direction *α*, the GLCM is a NxN matrix, whose (*i*, *j*) element is the counting of pixels of gray intensity level *i* which are adjacent (within a distance *ρ*) to pixels of the gray intensity level *j*. The GLRLM is an NxN matrix whose (*i*, *j*) element counts the number of runs of pixels of gray level *i* (run step 1) and run length *j* in a given direction. The same bin numbers (16, 32, and 64) used for FOS analysis were used for textural features computation. Range of ADC values for histogram creation was also the same (0–4000 *10^−6^ mm^2^/s). A distance *ρ* = 1 was used to create the GLCMs and GLRLMs.

For each patient, GLCMs and GLRLMs were created on 13 different directions. Textural features of Table 2 were computed on each matrix and the results averaged across all angles, thus obtaining two sets of features, one for the GLCM and one for the GLRLM. This average of the 13 different value is already been used in literature (see supplementary material of [6]) and it allows to deal with a lower dimensional features space (only one feature is considered instead of 13). All the algorithms were implemented in ITK 4.8 [3, 40].

Globally, 37 FOS, 21 GLCM-based, and 11 GLRLM-based features (69 in total) were considered for this analysis. Fifty-seven features out of 69 were bin-dependent and thus were computed three times, one for each histogram discretization.

### Stability and Discrimination Capacity Analysis

We developed a framework to assess features stability and discrimination capacity that is based on geometrical transformations (translations in particular) of the ROIs representing the GTV. The entire workflow was implemented in MATLAB 2016b (Mathworks, Natick, MA, USA).

**Fig. 1:**
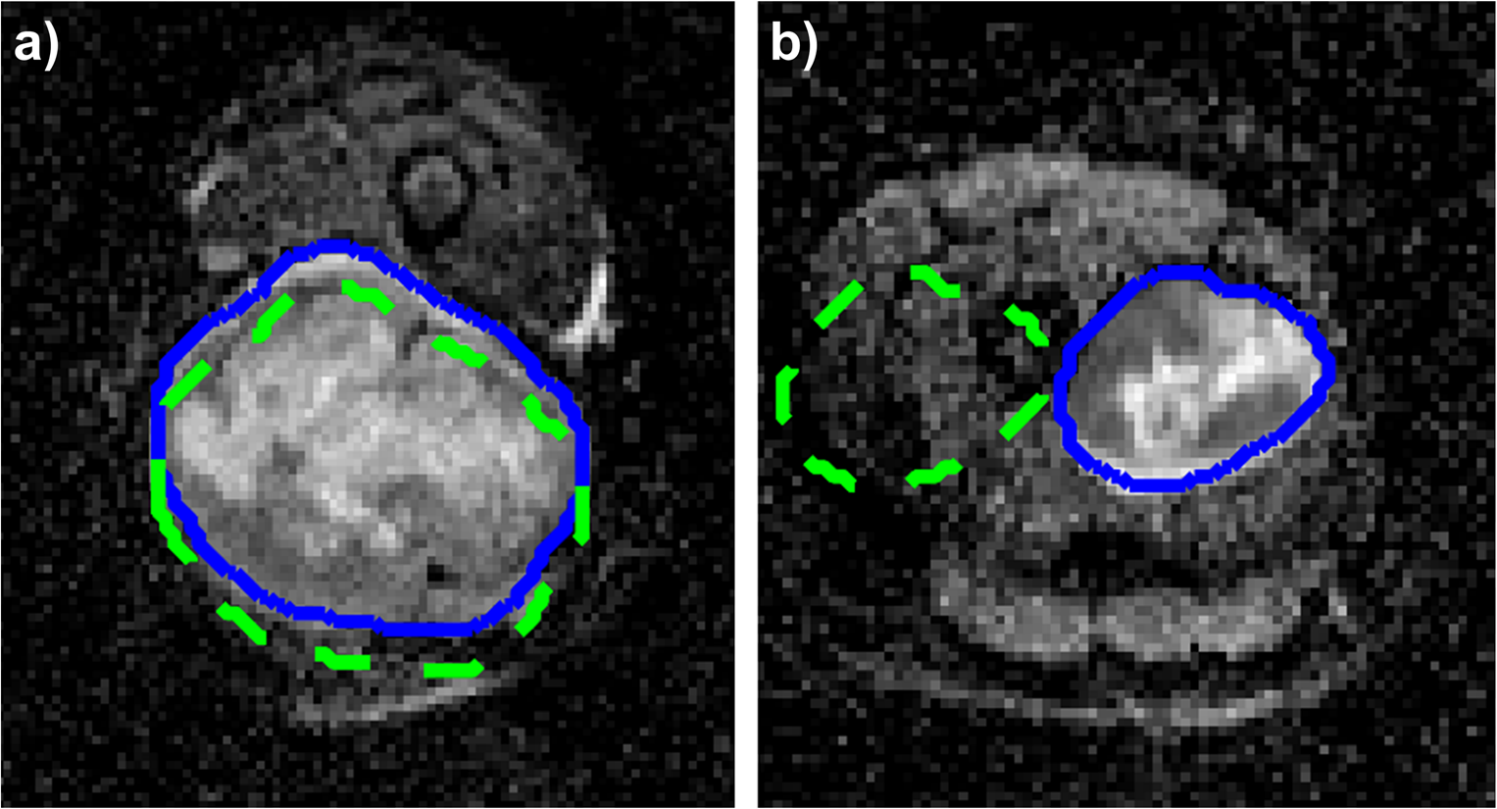
Example of translations applied to the regions of interest (ROIs). **a** Example of small entity translation in the *y* direction. **b** Example of maximal entity translation in the *x* direction. Continuous lines represent the contours of the original ROIs, while the dashed lines represent the contours of the modified ones

First, small entity translations were applied to the ROIs, along both the *x* (medial-lateral) and *y* (antero-posterior) directions. By small entity, we mean translations of ± 10% of the length of the bounding box surrounding the ROI in the direction of interest (Fig. 1a). We will also refer to this type of translation as minimal entity translation. We assume the variability due to such transformations to be comparable to the ones that could appear in a multiple delineations test. In total, for each ROI, four minimal entity translations were applied (one positive and one negative for both the *x* and *y* directions) and thus four transformed ROIs were obtained. The radiomic features were computed on the four transformed ROIs and compared to the ones obtained with the original one (the one segmented by the radiologist). Radiomic features were then compared using two similarity indexes: (1) percentage variation and (2) intra-class correlation coefficient (ICC).

For each comparison, the absolute percentage variation with respect to the reference was computed as follows:

1 $$ \mathrm{Diff}\%=\frac{\mid {F}_{\mathrm{Transf}}-{F}_{\mathrm{Original}}\mid }{\left|{F}_{\mathrm{Original}}\right|}\cdotp 100 $$

being ***F***_**Transf**_ and ***F***_**Original**_ the features computed on the transformed and original ROIs, respectively.

The ICC was computed as in [41, 42]: it measures the bivariate relation of variables representing different measurement classes and can be used to assess the agreement between data. The maximum value of ICC is 1, which indicates perfect agreement. The lower the ICC, the lower the similarity among the elements of the groups. In this study, a two-way mixed effect model was used (since the effect of the transformations is fixed and the variability for the different ROIs is random) [42].

For each feature, it is possible to compute 72 percentage variations (18 ROIs with 4 translations each) and 4 ICCs (one for each translation) and to compute the mean and standard deviation for both the distributions. Let us call the mean values obtained with such procedure ICC_mean_ and Diff%_mean_.

We repeat the above-described steps for increasing translation entities ranging from 10% (minimal entity translations) to 100% (maximal entity translations) with a step of 10%, and we computed the ICC_mean_ and Diff%_mean_ of the features for each translation, to evaluate how the similarity varies with the entity of the translations. In Fig. 1b, an example of maximal entity (± 100%) translation is represented. As it can be seen, this situation is far from the error range obtainable with multiple delineations. This type of transformation was used to evaluate discrimination capacity because, as previously stated, the underlying hypothesis is that if a feature remains constant independently on the entity of the translation, that feature is not going to be a good clinical descriptor.

ICC_mean_ was used to select the features with properties of stability and discrimination capacity. For this purpose, two ICC thresholds were used: a lower threshold for the ICC for the minimal entity translations (ICC_min_) and an upper ICC threshold for the maximal entity translations (ICC_max_). A feature is considered stable if the ICC_mean_ for the minimal entity translations (ICC_10_) is larger than ICC_min_ (ICC_10_ ***≥*** ICC_min_), and it is considered discriminative if the mean ICC_mean_ for the maximal entity translations (ICC_100_) is lower than ICC_max_ (ICC_100_ ***≤*** ICC_max_).

**Fig. 2:**
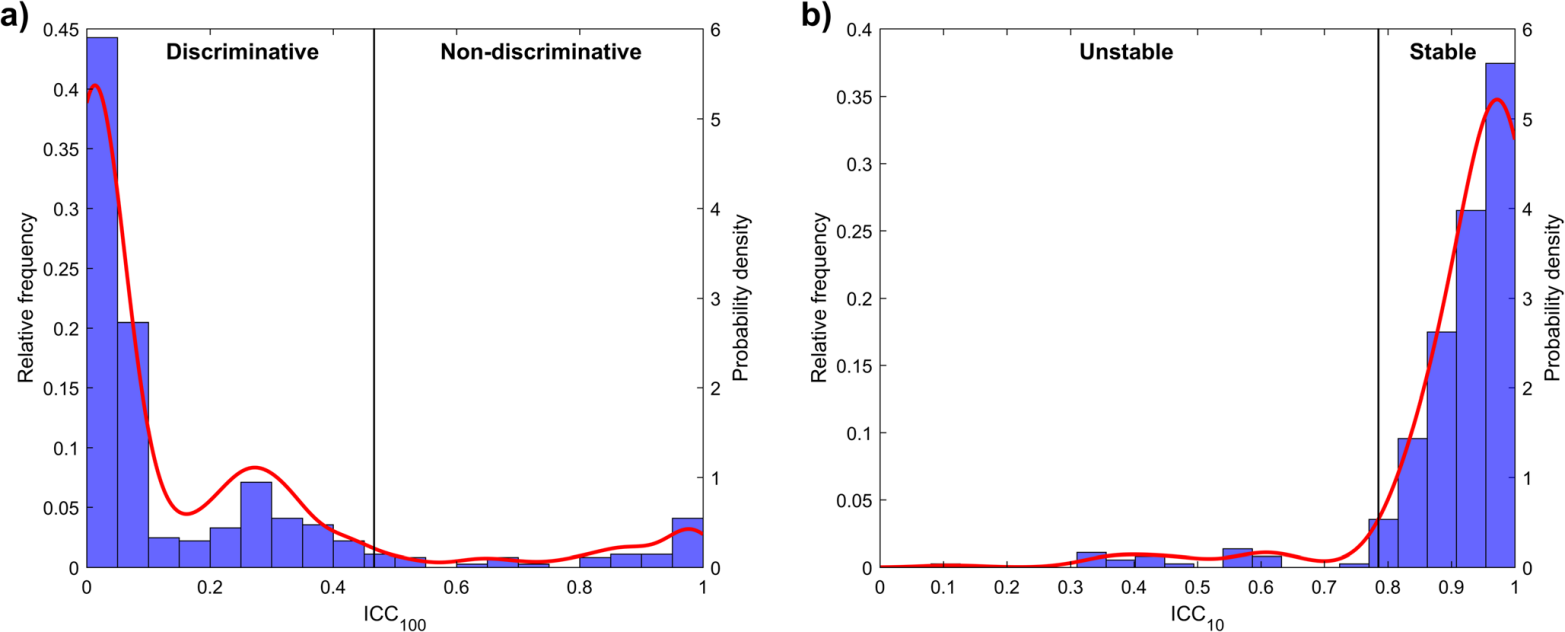
Continuous distribution fitted on the values of ICC_100_ (**a**) and ICC_10_ (**b**). In both cases, the reference quantile is marked with a line that divides the plot in two sections (discriminative/non-discriminative and stable/unstable respectively in **a** and **b**)

The two thresholds were set using information about the distributions of ICC_10_ and ICC_100_. The values of ICC_100_ for both the datasets and for all the bin discretizations are put together in the same histogram and, from this histogram, a continuous probability distribution is obtained (see Fig. 2). In particular, the probability distribution is a non-parametric kernel distribution fitted using MATLAB function *fitdist* (normal kernel, bandwidth 0.05). The value 0.05 was chosen as a good tradeoff to guarantee both smoothness of the curve and quality of the fitting (*p* > 0.05 for a *χ*^2^ test). ICC_max_ was defined as the quantile 0.9 of the continuous distribution previously defined. A similar procedure was used to define the ICC_min_ threshold starting from the histogram of all the ICC_10_, with the difference that the quantile used as a reference was 0.1.

The stability and discrimination capacity analysis is repeated 3 times, using 3 different bin numbers (16, 32, and 64 bins), to assess the effect of histogram discretization on the features. Jaccard’s index [43] was used to evaluate the similarity between the sets of excluded features for the different histogram discretizations, but also to compare excluded features in the two datasets.

## Results

The identified thresholds for ICC_min_ and ICC_max_ that were identified with the method explained in the previous section were 0.78 and 0.46, respectively.

**Fig. 3:**
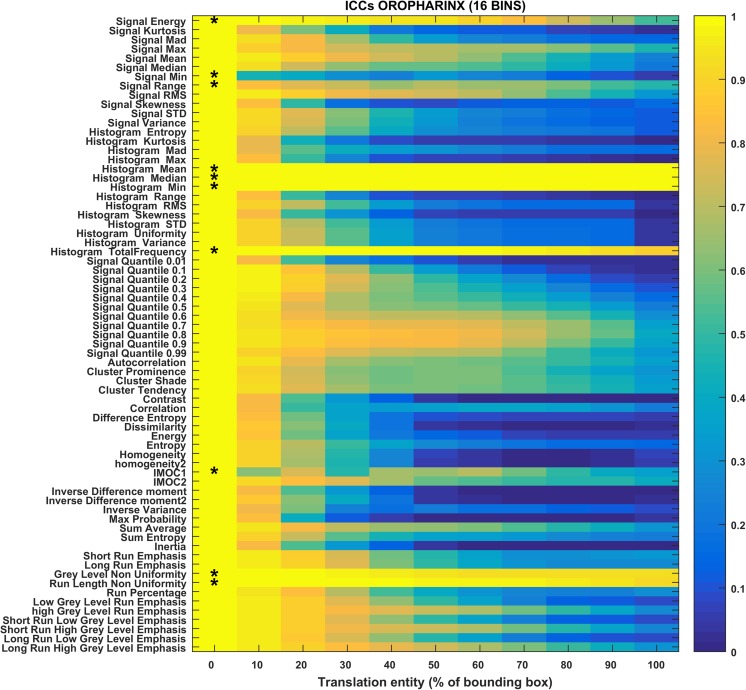
Heat map of the mean ICC_mean_ displayed according to features (rows) and entity of the translations (columns). The heat map refers to the oropharyngeal cancers (OPC) dataset and to the radiomic features computed with the 16-bin discretization. The features removed by the ICC-based feature selection technique are marked with an asterisk in the first column

**Fig. 4:**
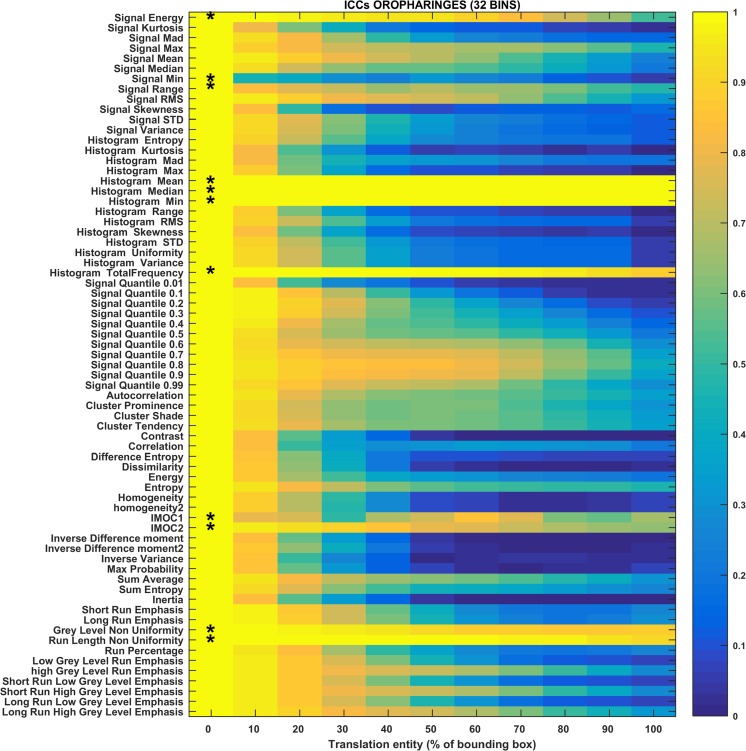
Heat map of the mean ICC_mean_ displayed according to features (rows) and entity of the translations (columns). The heat map refers to the oropharyngeal cancers (OPC) dataset and to the radiomic features computed with the 32-bin discretization. The features removed by the ICC-based feature selection technique are marked with an asterisk in the first column

**Fig. 5:**
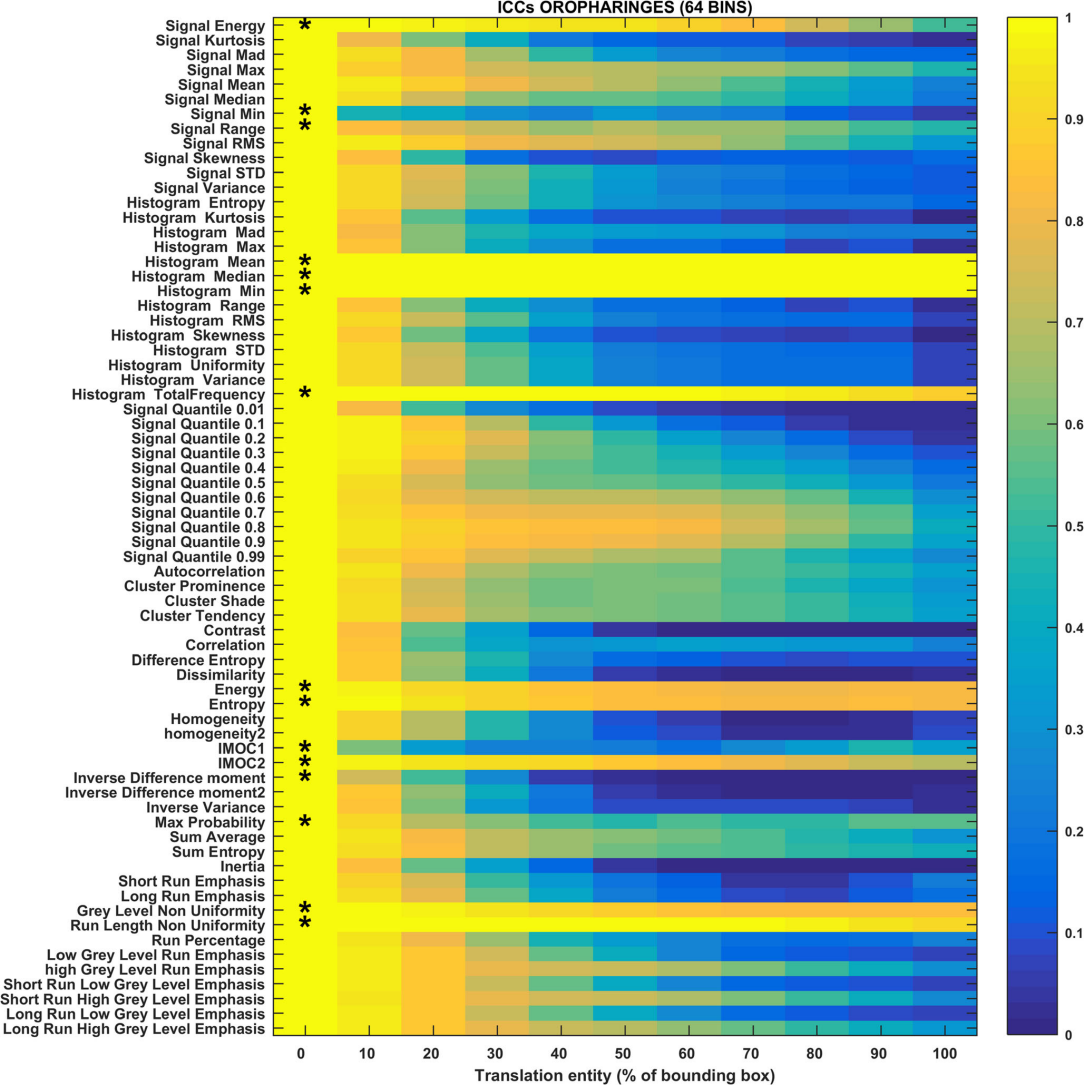
Heat map of the mean ICC_mean_ displayed according to features (rows) and entity of the translations (columns). The heat map refers to the oropharyngeal cancers (OPC) dataset and to the radiomic features computed with the 64-bin discretization. The features removed by the ICC-based feature selection technique are marked with an asterisk in the first column

**Fig. 6:**
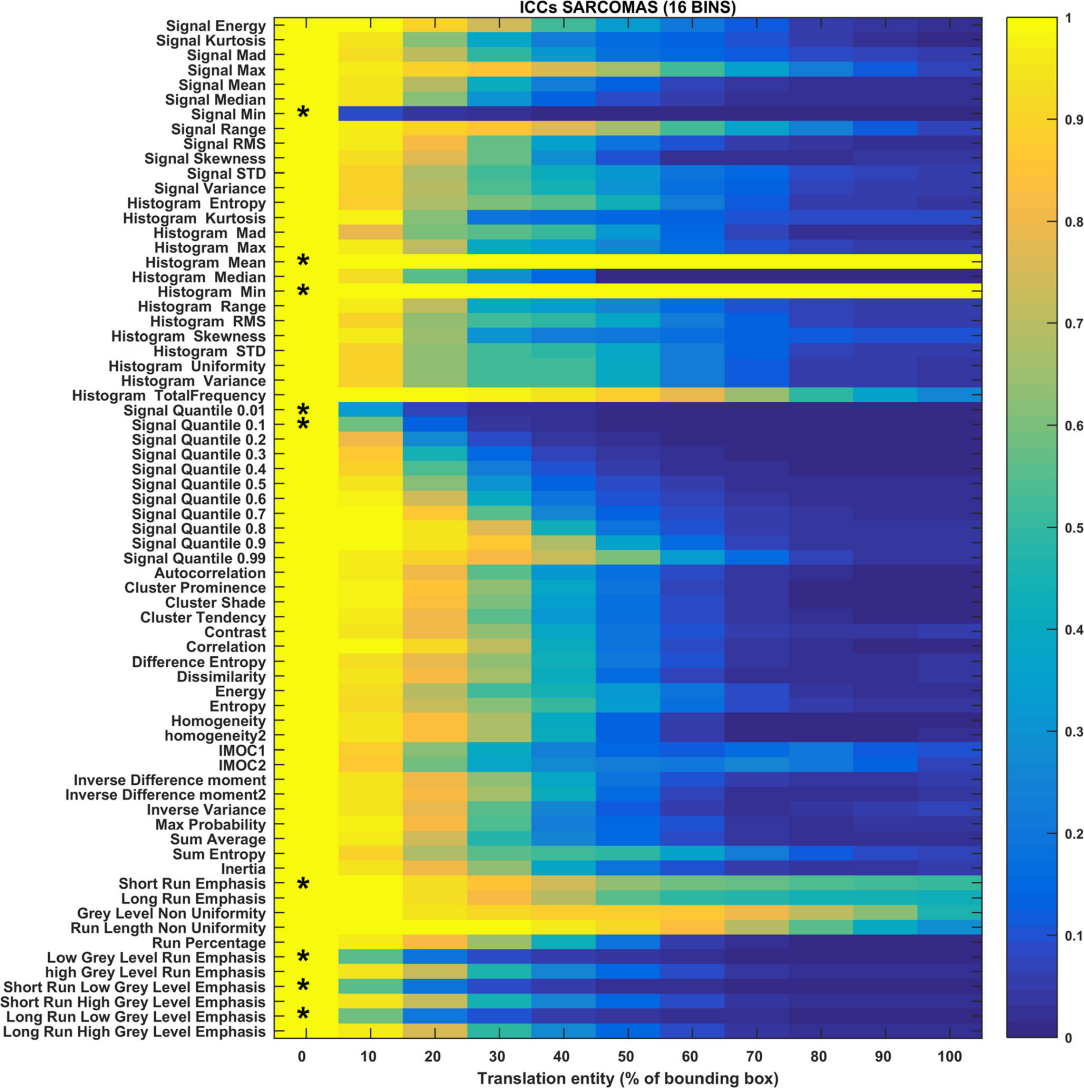
Heat map of the mean ICC_mean_ displayed according to features (rows) and entity of the translations (columns). The heat map refers to the soft tissue sarcoma (STS) dataset and to the radiomic features computed with the 16-bin discretization. The features removed by the ICC-based feature selection technique are marked with an asterisk in the first column

**Fig. 7:**
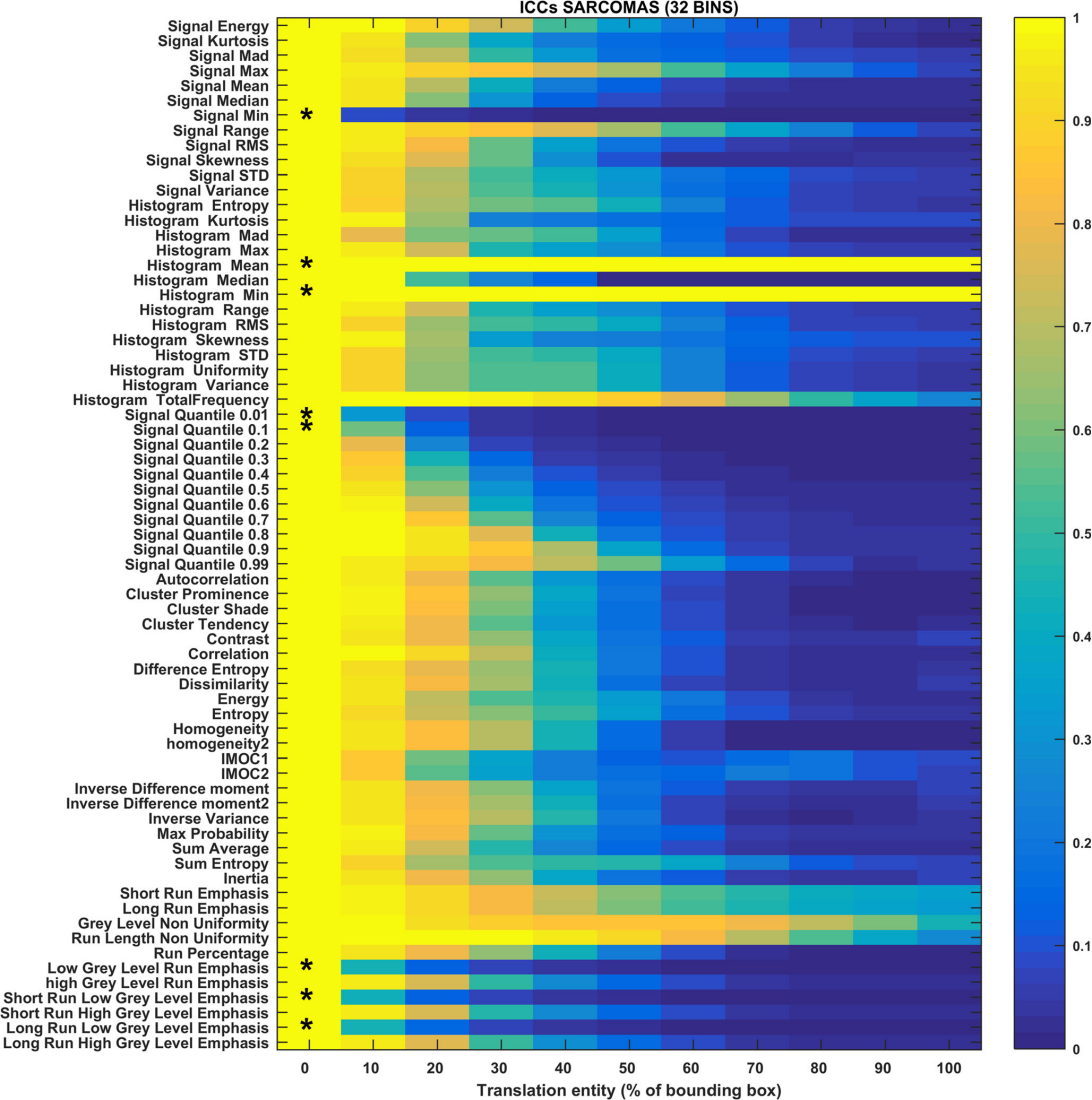
Heat map of the mean ICC_mean_ displayed according to features (rows) and entity of the translations (columns). The heat map refers to the soft tissue sarcoma (STS) dataset and to the radiomic features computed with the 32-bin discretization. The features removed by the ICC-based feature selection technique are marked with an asterisk in the first column

**Fig. 8:**
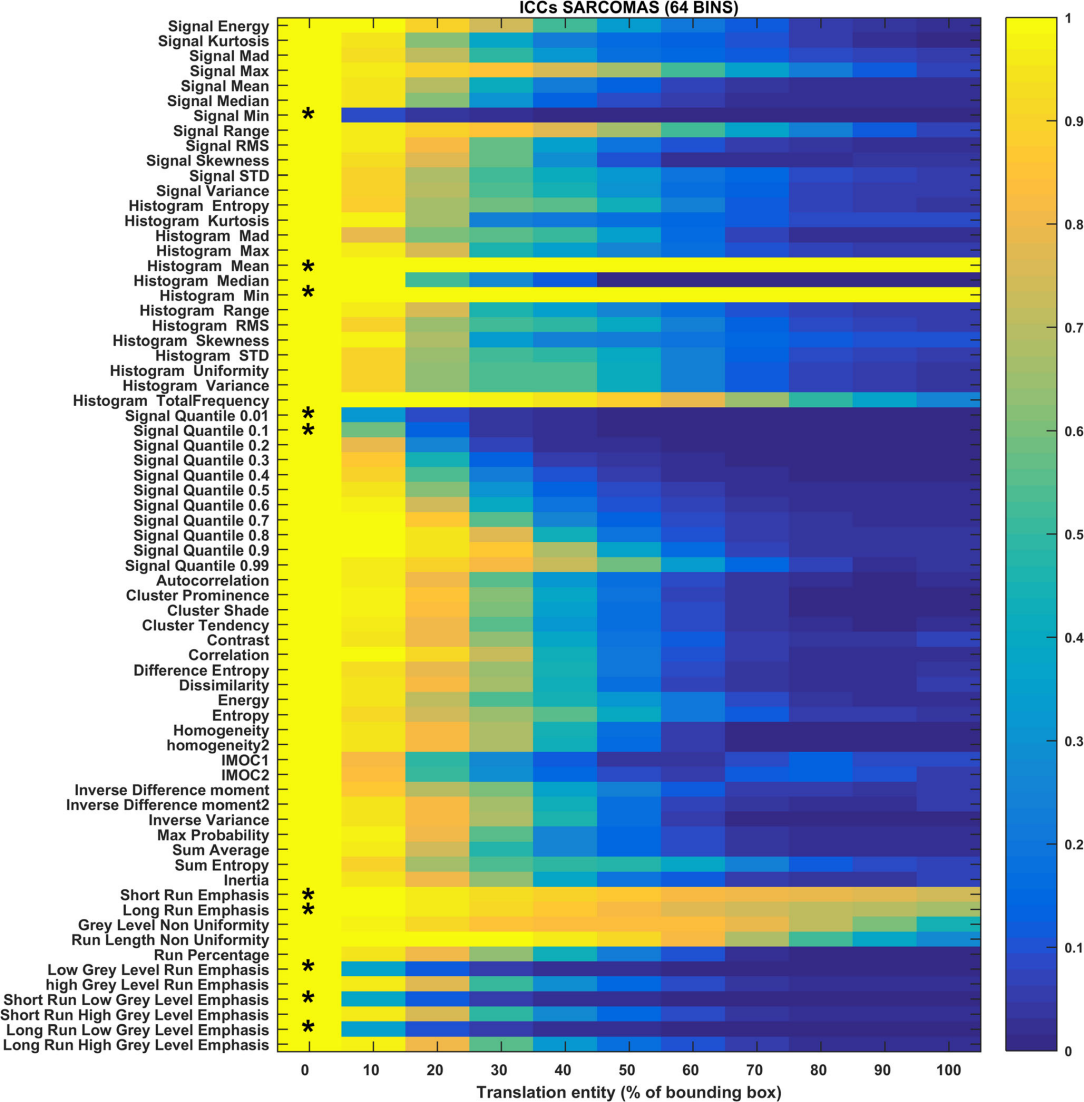
Heat map of the mean ICC_mean_ displayed according to features (rows) and entity of the translations (columns). The heat map refers to the soft tissue sarcoma (STS) dataset and to the radiomic features computed with the 64-bin discretization. The features removed by the ICC-based feature selection technique are marked with an asterisk in the first column

**Fig. 9:**
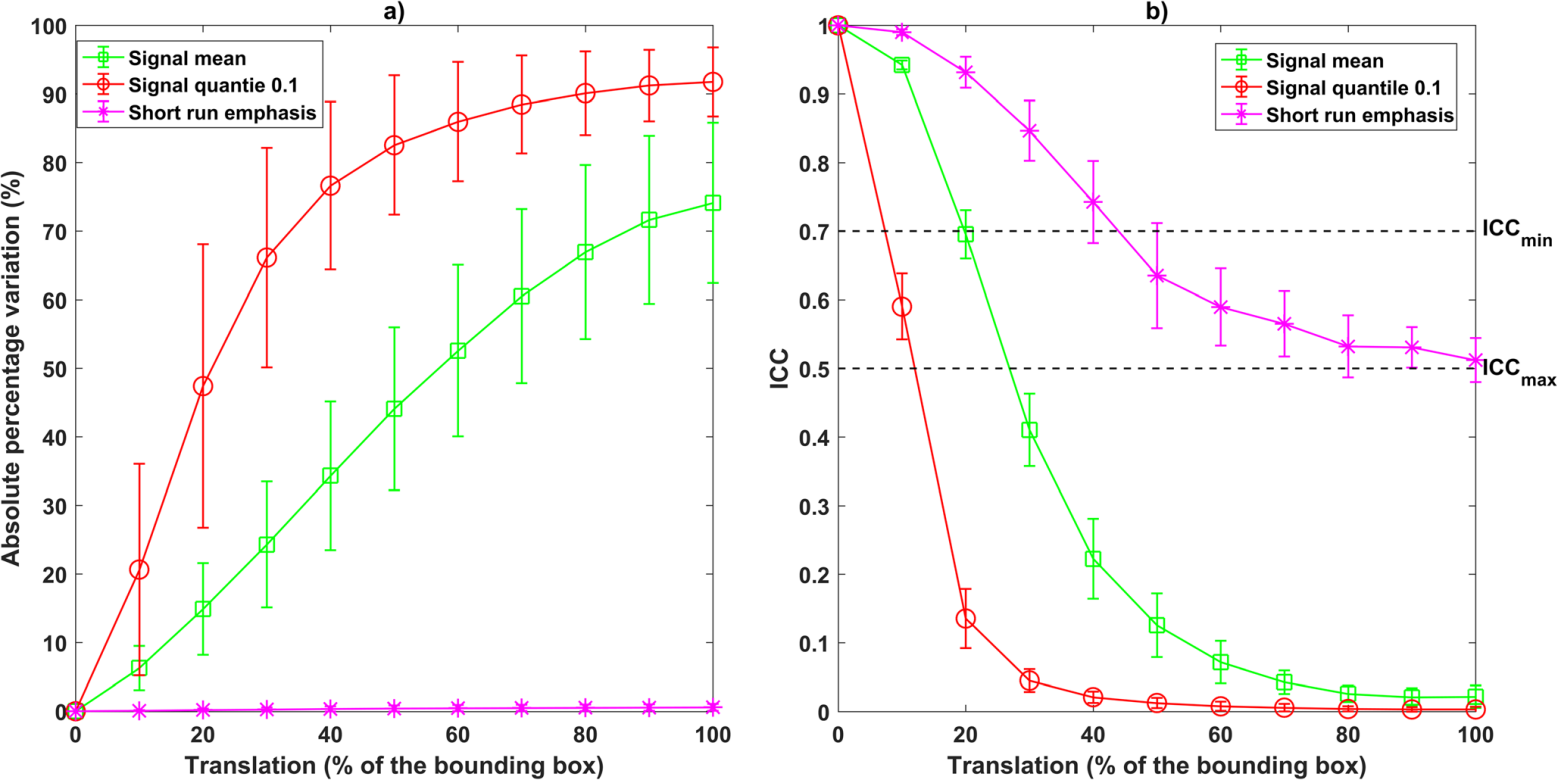
Plot representing the variation with respect to entity of translation for 3 different radiomic features measured on the soft tissue sarcoma (STS) dataset, with 16-bin discretization. **a** Absolute percentage variation plot. **b** ICC variation. One representative of each group of feature is represented: signal mean (squared markers) is both stable and discriminative; signal quantile 0.1 (circular markers) is unstable; short run emphasis (asterisks) is non-discriminative. Both mean values and 95% confidence interval are shown

The heat maps in Figs. 3, 4, 5, 6, 7, and 8 show how the level of ICC_mean_ varies with the entity of the translations in the two datasets. Figures 3, 4, and 5 show the ICC_mean_ maps related to the OPC dataset using the three different histogram subdivisions, while Figs. 6, 7, and 8 show the ICC_mean_ maps for the STS dataset. In Fig. 9a, examples of Diff%_mean_ plot (with 95% confidence interval) for an unstable feature (signal quantile 0.1), a non-discriminative feature (short run emphasis), and a feature that is selected by the algorithm (signal mean) in the STS dataset can be seen. In Fig. 9b, the plot of ICC_mean_ (with 95% confidence interval) for the same features can be seen. Since it is not possible to represent all the values of percentage variations and ICC, we refer to Tables 1–20 in the online resources, containing all the values of ICC_10_ and ICC_100_, together with the corresponding percentage variations.

**Fig. 10:**
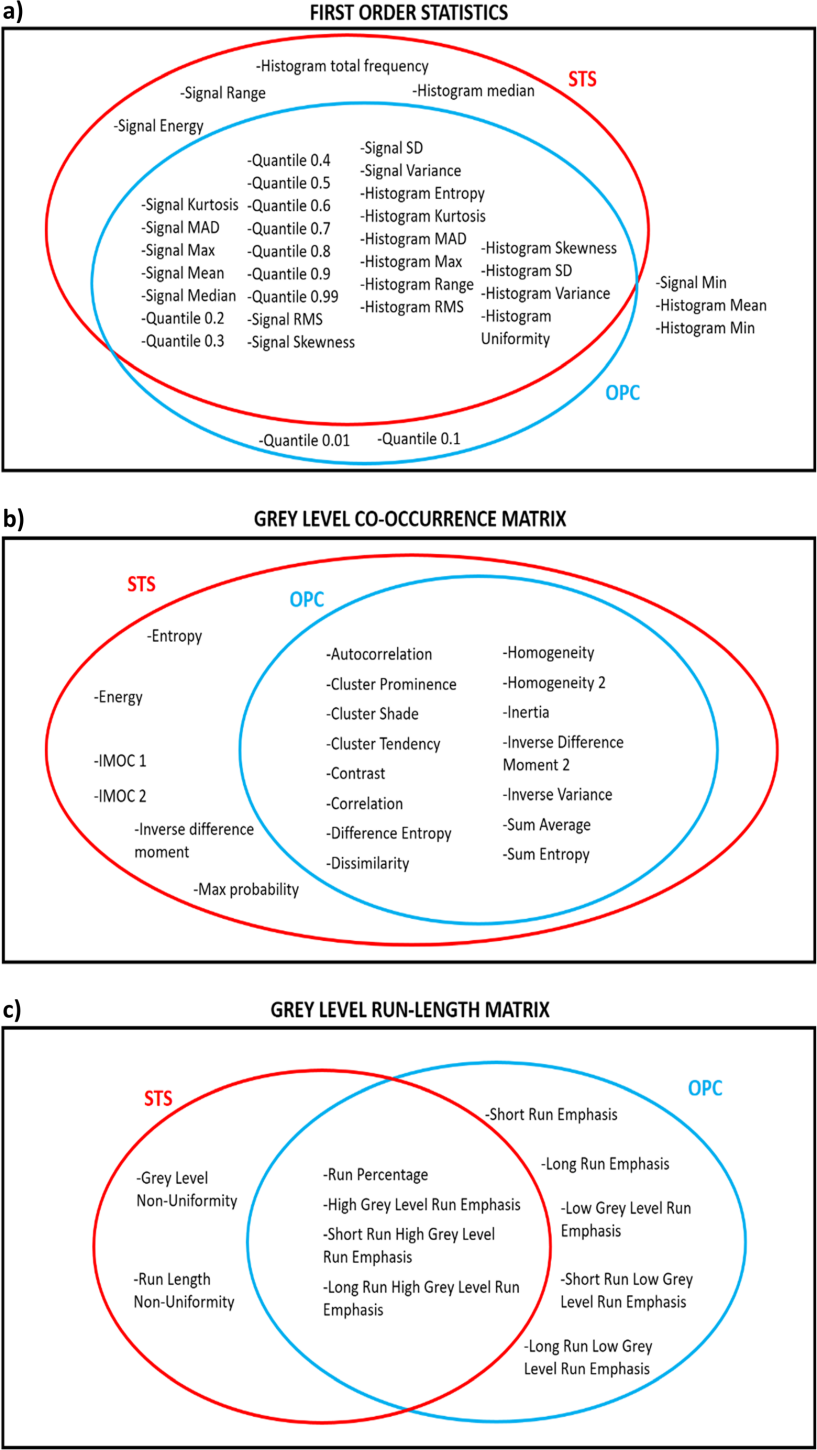
Euler-Venn diagram representing the accepted features divided by group. **a** First-order statistics. **b** Gray-level co-occurrence matrix. **c** Gray-level run length matrix. Selected features are grouped by dataset: the soft tissue sarcoma (STS) dataset and oropharyngeal cancer (OPC) dataset

Table 3 lists the features removed with our ICC-based feature selection method. The six boxes show the results in the two datasets with each of the three histogram discretizations. The ICC-based feature selection method removes 8–15 features. If we consider the features that are stable for all the three histogram discretizations, the method selects 54 features out of 69 for the OPC dataset and 59 features out of 69 for the STS dataset. Such features, divided by groups, are shown in the Euler-Venn diagrams in Fig. 10. If we take into account the three subsets of the excluded features for the three histogram subdivisions and we compute the Jaccard’s similarity index for the three possible combinations, we obtain a value of 0.77 ± 0.13 for the OPC dataset and 0.9 ± 0.1 for the STS dataset. If we compare the set of excluded features for the OPC and STS dataset for each of the three histogram discretizations, we get a Jaccard’s index of 0.17 ± 0.03.

**Table 3:** Features removed by the ICC-based feature selection algorithm

16 bins	32 bins	64 bins
OPC dataset		
-Signal energy -Signal minimum -Signal range -Histogram mean -Histogram median -Histogram minimum -Histogram total frequency -Information measure of correlation 1 (IMOC1) -Gray-level non-uniformity -Run length non-uniformity	-Signal energy -Signal minimum -Signal range -Histogram mean -Histogram median -Histogram minimum -Histogram total frequency -Information measure of correlation 1 (IMOC1) -Information measure of correlation 2 (IMOC2) -Gray-level non-uniformity -Run length non-uniformity	-Signal energy -Signal minimum -Signal range -Histogram mean -Histogram median -Histogram minimum -Histogram total frequency -Energy -Entropy -Information measure of correlation 1 (IMOC1) -Information measure of correlation 2 (IMOC2) -Inverse difference moment -Max probability -Gray-level non-uniformity -Run length non-uniformity
STS dataset		
-Signal minimum -Signal quantile 0.01 -Signal quantile 0.1 -Histogram mean -Histogram minimum -Short run emphasis -Low gray-level run emphasis -Short run low gray-level emphasis -Long run low gray-level emphasis	-Signal minimum -Signal quantile 0.01 -Signal quantile 0.1 -Histogram mean -Histogram minimum -Low gray-level run emphasis -Short run low gray-level emphasis -Long run low gray-level emphasis	-Signal minimum -Signal quantile 0.01 -Signal quantile 0.1 -Histogram mean -Histogram minimum -Short run emphasis -Long run emphasis -Low gray-level run emphasis -Short run low gray-level emphasis -Long run low gray-level emphasis

## Discussion

The assessment of features stability is an important preliminary step in any radiomic analysis. In this study, we developed a new method to assess the stability and the discrimination capacity of radiomic features computed from medical images (in this case DW-MRI images). In particular, we proposed a fast way to assess features stability and discrimination capacity without the need of multiple acquisitions or multiple delineations, thus performing a preliminary step of feature selection.

Both in STS and OPC datasets, features can be divided in three groups: (I) features whose ICC decreases gradually but constantly; (II) features whose ICC sharply decreases; (III) features that remain similar for all translations. These three groups can be approximately considered as (I) the stable and discriminative features, (II) unstable features, and (III) stable and non-discriminative features, respectively.

In the STS dataset, the ICC-based feature selection removes the features in group II (unstable features) and many of the ones of group III (non-discriminative features). However, there are some features for which ICC_100_ is slightly under the threshold that are therefore not considered as non-discriminant (histogram total frequency and some GLRLM-based features matrix). Some of these features are removed for some of the histogram discretizations (e.g., short and long run emphasis).

Something similar can be said for the features in the OPC dataset in Figs. 3, 4, and 5. There are features, like signal energy, gray-level non-uniformity, and run length non-uniformity, that are removed because they remain very similar inside and outside the tumor. There are also features, like signal minimum, that are too unstable and drastically change even for small translations. Some features, like the information measures of correlation, present an ICC that is very close to the threshold and therefore they are excluded just for some histogram discretizations. Two features (entropy and energy) strongly change their behavior according to histogram discretization. It can be seen that for 16-bin discretization, the ICC level for those features decreases quite gradually, and the features are accepted according to our method. Using the 64-bin discretization, their values of ICC remain almost constant and the features are considered non-discriminative. The increase in entropy with the number of bins is predictable: more bins means more gray levels and more disorder. However, the fact that the change in the measured ICC is so high, it is worth noting. The fact that both energy and entropy have high dependency on the histogram discretization is also reported in [44]. Max probability also changes its stability behavior for the 64-bin discretization, similarly to what happens for entropy. Last, ICC_10_ for inverse difference moment is close to the threshold of stability and the feature is labeled as unstable when the 64-bin discretization is used.

Although the behavior of some features, like energy and entropy, is highly dependent on the number of bins used, in general, the results of the ICC-based feature selection do not depend on histogram discretization. The type of tumor, instead, strongly affects the excluded features. There are only three common features between the datasets. Signal minimum is unstable as it can be expected since it is an extreme value of a distribution. Histogram mean is always constant throughout all the translation because it only depends on the number of bins. Histogram minimum is 0 when there is at least one empty bin in the histogram, which is very common; therefore, the feature is non-discriminative. This is true at least for the histogram subdivisions that were used in this study.

To our knowledge, this is the first time that both small and large translations of the ROI are used to evaluate fatures stability and discriminative power respectively. It is also the first time in which the thresholds of ICC used to distinguish the type of feature (stable, unstable, or non-discriminative) are not empirically set.

The values of ICC for small transformations computed for the radiomic features analyzed in this study are around 0.9 (median 0.94, quartiles 0.89 and 0.97). In [12], similar values of ICC are found for the stable features (median 0.97, quartiles 0.92 and 0.99). The Mann-Whitney test reveals no significant difference between the ICC values of the stable features identified in the current study and in [12] (*p* = 0.92). However, a smaller number of features is actually stable (18 out of 79). This could depend from the fact that in the present study and [12], the features set used is not the same.

Compared to a study in which features stability is assessed through multiple manual delineation, like [18], the values of ICC found for small translations are higher than the ones found for multiple delineations (median 0.94 vs median 0.89, Mann-Whitney test *p* < 0.01). The initial assumption that the low entity translations are equivalent to multiple delineations in terms of evaluating stability seems to be rejected, even though the differences in the ICC values could also depend on the different imaging technique (MRI vs PET) and in the different region of the body analyzed (lung vs limbs and head and neck). According to such findings, our method is potentially less restrictive for the assessment of stability, but for this reason, we can be sure that the features that we identify as unstable are indeed unstable. Moreover, if a more restrictive method is required, the translation considered for stability analysis could be increased to 15–20% of the bounding box.

In this paper, as opposed to [12], we presented only translations of the ROIs and we did not show the effect of rotation, dilatation, and shrinking. Those types of transformations were also applied in our investigation but their use did not influence the results of the ICC-based feature selection method, and therefore they were not reported (for further details, refer to the Tables 21–60 of the online resources).

The method presented in this study has some advantages over other methods of literature. Compared to [27], it does not need a digital biopsy, which requires a further segmentation step, although a digital biopsy takes less time to be segmented than a normal ROI. Compared to a method based on [28], it requires no segmentation algorithm, which can be difficult to design for oropharyngeal tumors. Last, the presented method allows to evaluate not only stability, but also the discriminative power of the features, which is something that, to the knowledge of the authors, was never considered before.

This study highlights the difference in stability of the radiomic features for tumors in different regions of the body, which is not typically done. As a matter of fact, the majority of the studies on stability of radiomic features focuses on tumors in a specific region of the body: esophagus [17], liver [19], brain [12], lung [22], or kidney [23]. A study analyzing multiple body regions exists [24], but even though the data come from multiple sources, they are analyzed all together and differentiation in the stability behavior for the different body regions is not explored. In this paper, we observed that radiomic features from tumors in the head and neck region (OPC dataset) present in general lower stability to small translations than tumors in the limbs (STS dataset). In fact, the values of ICCs for small translations are significantly higher in the STS dataset (Wilcoxon signed rank test *p* < 0.01; see also online resources, Tables 1–20). This result could come from the fact that sarcomas have larger volume and small translations have less effect on features that are computed on the entire ROI. The opposite happens when we consider the ICCs for large transformations (Wilcoxon signed rank test *p* < 0.01; see also online resources, Tables 1–20). This could depend from the fact that the contrast between tumoral and healthy tissue in ADC images is different for the two types of cancer. As a matter of fact, sarcomas have higher contrast and are much easier to distinguish, rather than head and neck tumors.

We think that the presented study could provide a better understanding of radiomic features stability for DW-MRI. It is worth underlining that this methodology should be used just as a preliminary feature selection. In fact, of the 69 radiomic features that were analyzed, only 8–15 are excluded by our algorithm, which is about 10–20% of the total number features. In order to further reduce the number of selected features, a possible approach could be to add a correlation-based (as shown in [16]) or a wrapper feature selection method after the ICC-based analysis. A limitation of this approach is that it cannot be used for geometrical features like shape and size or location (which are also used in [16]) since the shape and size of each ROI are kept constant throughout all the experiment, while the ROI location is changed. A possible solution to this could be to apply random combination of geometrical transformations to mimic the effects of random multiple delineations or ROI registration, and we plan to investigate this in further studies.

## Conclusion

In this study, a method to assess the stability and the discrimination capacity of the radiomic features has been developed, using small and large translations of the ROI. The method was applied to two independent datasets containing DW-MRI images of different tumors (oropharyngeal tumors and sarcomas). The proposed method excluded 10–20% of the original features set.

We think that the presented study could provide a better understanding of radiomic features stability and discrimination capacity for DW-MRI, providing a way to assess features stability without the need of multiple acquisitions or delineations.

## Electronic Supplementary Material

ESM 1 (DOCX 166 kb)
