# Supplementary material for: Assessment of Stability and Discrimination Capacity of Radiomic Features on Apparent Diffusion Coefficient Images
Source: J Digit Imaging. 2018 May 3;31(6):879–94. doi: 10.1007/s10278-018-0092-9 (PMC6261192; doi:10.1007/s10278-018-0092-9)
Supplement: Supplementary file 1 — (DOCX 166 kb) [file 10278_2018_92_MOESM1_ESM.docx]

**Assessment of Stability and Discrimination Capacity of Radiomic Features on apparent diffusion coefficient images (online resource)**

Marco Bologna^1*^, Valentina D. A. Corino^1^, Eros Montin^1^, Antonella Messina^2^, Giuseppina Calareso^2^, Francesca G. Greco^2^, Silvana Sdao^2^, Luca T. Mainardi^1^

^1^ *Department of Electronics, Information and Bioengineering, Politecnico di Milano, Milan, Italy*

^2^ *Dipartimento di Diagnostica per Immagini e Radioterapia, Fondazione IRCCS Istituto Nazionale dei Tumori, Milan, Italy*

* Corresponding author: Marco Bologna; Via Golgi 39, 20133 Milan, Italy; +39 0223993322; marco.bologna@polimi.it

1. **TABLES OF FEATURES (TRANSLATIONS)**

In this section the tables with the percentage variation of radiomic features for minimum and maximum entity translations, together with the values of ICC_10_ and ICC_100_. The values are shown in the following tables and divided by histogram discretization and by dataset.

- 1. **Oropharyngeal cancers dataset**

**Table 1** First-Order Statistics (FOS) features for the patients of the OroPharyngeal Cancers (OPC) dataset. In the table, the bin-independent features are presented. The first column shows the mean and standard deviations of the original radiomic features computed on all the patients. The mean and standard deviation of both percentage difference and Intra-class Correlation Coefficient (ICC) are shown for both minimal and maximal entity translations. Features excluded by our method are highlighted in red. Values are shown with scientific format only for the original features. The numbers are approximated to the second decimal significant digit.

| **FIRST-ORDER STATISTICS (BIN-INDEPENDENT)** | | | | | |
| --- | --- | --- | --- | --- | --- |
| **Feature name** | **Original value** | **Diff %**  **(min. entity)** | **Diff %**  **(max. entity)** | **ICC_10_** | **ICC_100_** |
| Signal Energy | 2.37e+09 ± 1.88e+09 | 6.84 ± 5.61 | 36.28 ± 29.15 | 0.99 ± 0.00 | 0.53 ± 0.25 |
| Signal Kurtosis | 3.24e+00 ± 7.00e-01 | 9.59 ± 7.71 | 29.54 ± 22.90 | 0.81 ± 0.06 | 0.03 ± 0.06 |
| Signal Mad | 3.01e+02 ± 5.65e+01 | 5.83 ± 4.81 | 33.19 ± 30.03 | 0.93 ± 0.02 | 0.15 ± 0.17 |
| Signal Max | 2.41e+03 ± 4.05e+02 | 4.25 ± 7.31 | 15.84 ± 12.01 | 0.89 ± 0.05 | 0.46 ± 0.05 |
| Signal Mean | 1.05e+03 ± 1.55e+02 | 3.57 ± 2.55 | 21.96 ± 17.30 | 0.96 ± 0.01 | 0.24 ± 0.22 |
| Signal Median | 9.83e+02 ± 1.46e+02 | 4.64 ± 4.60 | 26.73 ± 20.70 | 0.92 ± 0.05 | 0.21 ± 0.16 |
| Signal Min | 1.01e+02 ± 1.21e+02 | 265.07 ± 1408.66 | 226.12 ± 712.97 | 0.45 ± 0.20 | 0.05 ± 0.09 |
| Signal Range | 2.31e+03 ± 4.44e+02 | 7.46 ± 9.98 | 17.72 ± 14.02 | 0.83 ± 0.04 | 0.47 ± 0.09 |
| Signal RMS | 1.12e+03 ± 1.50e+02 | 3.31 ± 2.63 | 17.14 ± 14.32 | 0.96 ± 0.02 | 0.31 ± 0.24 |
| Signal Skewness | 5.10e-01 ± 3.50e-01 | 35.18 ± 35.64 | 139.48 ± 162.60 | 0.84 ± 0.04 | 0.16 ± 0.12 |
| Signal STD | 3.72e+02 ± 5.77e+01 | 5.38 ± 4.51 | 29.69 ± 27.05 | 0.92 ± 0.02 | 0.12 ± 0.19 |
| Signal Variance | 1.42e+05 ± 4.29e+04 | 10.99 ± 9.56 | 73.32 ± 82.15 | 0.91 ± 0.02 | 0.12 ± 0.15 |

**Table 2** First-Order Statistics (FOS) features for the patients of the OroPharyngeal Cancers (OPC) dataset. In the table, the bin-dependent features computed for 16-bins histogram discretization are presented. The first column shows the mean and standard deviations of the original radiomic features computed on all the patients. The mean and standard deviation of both percentage difference and Intra-class Correlation Coefficient (ICC) are shown for both minimal and maximal entity translations. Features excluded by our method are highlighted in red. Values are shown with scientific format only for the original features. The numbers are approximated to the second decimal significant digit.

| **FIRST-ORDER STATISTICS (16-BINS DISCRETIZATION)** | | | | | |
| --- | --- | --- | --- | --- | --- |
| **Feature name** | **Original value** | **Diff %**  **(min. entity)** | **Diff %**  **(max. entity)** | **ICC_10_** | **ICC_100_** |
| Histogram Entropy | 2.51e+00 ± 1.90e-01 | 3.05 ± 2.06 | 12.46 ± 10.63 | 0.89 ± 0.03 | 0.11 ± 0.15 |
| Histogram Kurtosis | 9.46e+00 ± 2.21e+00 | 11.38 ± 9.99 | 45.22 ± 37.14 | 0.79 ± 0.11 | 0.01 ± 0.03 |
| Histogram Mad | 5.00e-02 ± 0.00e+00 | 1.42 ± 1.05 | 4.86 ± 4.77 | 0.80 ± 0.07 | 0.16 ± 0.20 |
| Histogram Max | 3.20e-01 ± 5.00e-02 | 7.29 ± 5.68 | 28.57 ± 22.36 | 0.84 ± 0.08 | 0.00 ± 0.00 |
| Histogram Mean | 3.00e-02 ± 0.00e+00 | 0.00 ± 0.00 | 0.00 ± 0.00 | 1.00 ± 0.00 | 1.00 ± 0.00 |
| Histogram Median | 0.00e+00 ± 0.00e+00 | 0.00 ± 0.00 | 0.00 ± 0.00 | 1.00 ± 0.00 | 1.00 ± 0.00 |
| Histogram Min | 0.00e+00 ± 0.00e+00 | 0.00 ± 0.00 | 0.00 ± 0.00 | 1.00 ± 0.00 | 1.00 ± 0.00 |
| Histogram Range | 3.20e-01 ± 5.00e-02 | 7.29 ± 5.68 | 28.57 ± 22.36 | 0.84 ± 0.08 | 0.00 ± 0.00 |
| Histogram RMS | 8.00e-02 ± 1.00e-02 | 2.90 ± 2.07 | 12.97 ± 9.64 | 0.90 ± 0.03 | 0.04 ± 0.05 |
| Histogram Skewness | 2.67e+00 ± 3.60e-01 | 6.45 ± 5.70 | 26.54 ± 17.84 | 0.82 ± 0.10 | 0.00 ± 0.00 |
| Histogram STD | 8.00e-02 ± 1.00e-02 | 3.42 ± 2.45 | 15.35 ± 11.41 | 0.90 ± 0.03 | 0.04 ± 0.05 |
| Histogram Uniformity | 2.10e-01 ± 3.00e-02 | 5.71 ± 4.01 | 25.61 ± 21.03 | 0.90 ± 0.02 | 0.05 ± 0.05 |
| Histogram Variance | 1.00e-02 ± 0.00e+00 | 6.72 ± 4.72 | 30.20 ± 25.29 | 0.90 ± 0.02 | 0.05 ± 0.05 |
| Histogram TotalFrequency | 1.81e+03 ± 1.31e+03 | 1.19 ± 1.15 | 12.22 ± 14.41 | 1.00 ± 0.00 | 0.88 ± 0.07 |
| Signal Quantile 0.01 | 3.25e+02 ± 1.88e+02 | 27.68 ± 22.93 | 94.05 ± 145.67 | 0.82 ± 0.05 | 0.02 ± 0.04 |
| Signal Quantile 0.1 | 6.10e+02 ± 1.72e+02 | 5.91 ± 6.11 | 55.89 ± 29.72 | 0.97 ± 0.02 | 0.03 ± 0.05 |
| Signal Quantile 0.2 | 7.24e+02 ± 1.61e+02 | 4.16 ± 4.17 | 44.40 ± 26.74 | 0.98 ± 0.01 | 0.05 ± 0.08 |
| Signal Quantile 0.3 | 8.16e+02 ± 1.52e+02 | 3.65 ± 2.86 | 37.27 ± 24.36 | 0.98 ± 0.01 | 0.10 ± 0.09 |
| Signal Quantile 0.4 | 9.01e+02 ± 1.46e+02 | 3.74 ± 2.84 | 31.68 ± 22.62 | 0.97 ± 0.01 | 0.16 ± 0.12 |
| Signal Quantile 0.5 | 9.93e+02 ± 1.45e+02 | 4.15 ± 3.66 | 25.96 ± 20.34 | 0.94 ± 0.03 | 0.22 ± 0.17 |
| Signal Quantile 0.6 | 1.10e+03 ± 1.53e+02 | 4.21 ± 3.83 | 20.30 ± 18.39 | 0.92 ± 0.02 | 0.29 ± 0.21 |
| Signal Quantile 0.7 | 1.23e+03 ± 1.72e+02 | 3.92 ± 3.39 | 16.03 ± 16.02 | 0.94 ± 0.01 | 0.35 ± 0.23 |
| Signal Quantile 0.8 | 1.38e+03 ± 1.85e+02 | 3.48 ± 2.87 | 14.24 ± 13.08 | 0.95 ± 0.02 | 0.40 ± 0.20 |
| Signal Quantile 0.9 | 1.58e+03 ± 1.99e+02 | 3.41 ± 2.87 | 13.93 ± 12.03 | 0.95 ± 0.03 | 0.37 ± 0.16 |
| Signal Quantile 0.99 | 2.04e+03 ± 2.34e+02 | 3.59 ± 4.29 | 14.85 ± 13.38 | 0.90 ± 0.05 | 0.32 ± 0.14 |

**Table 3** First-Order Statistics (FOS) features for the patients of the OroPharyngeal Cancers (OPC) dataset. In the table, the bin-dependent features computed for 32-bins histogram discretization are presented. The first column shows the mean and standard deviations of the original radiomic features computed on all the patients. The mean and standard deviation of both percentage difference and Intra-class Correlation Coefficient (ICC) are shown for both minimal and maximal entity translations. Features excluded by our method are highlighted in red. Values are shown with scientific format only for the original features. The numbers are approximated to the second decimal significant digit.

| **FIRST-ORDER STATISTICS (32-BINS DISCRETIZATION)** | | | | | |
| --- | --- | --- | --- | --- | --- |
| **Feature name** | **Original value** | **Diff %**  **(min. entity)** | **Diff %**  **(max. entity)** | **ICC_10_** | **ICC_100_** |
| Histogram Entropy | 3.47e+00 ± 2.00e-01 | 2.29 ± 1.51 | 9.56 ± 7.76 | 0.90 ± 0.03 | 0.12 ± 0.16 |
| Histogram Kurtosis | 1.08e+01 ± 2.89e+00 | 11.78 ± 10.13 | 46.28 ± 35.31 | 0.82 ± 0.12 | 0.01 ± 0.01 |
| Histogram Mad | 2.00e-02 ± 0.00e+00 | 1.33 ± 1.11 | 4.85 ± 4.83 | 0.82 ± 0.05 | 0.20 ± 0.24 |
| Histogram Max | 1.80e-01 ± 4.00e-02 | 7.63 ± 5.71 | 32.20 ± 22.05 | 0.88 ± 0.06 | 0.00 ± 0.00 |
| Histogram Mean | 2.00e-02 ± 0.00e+00 | 0.00 ± 0.00 | 0.00 ± 0.00 | 1.00 ± 0.00 | 1.00 ± 0.00 |
| Histogram Median | 0.00e+00 ± 0.00e+00 | 0.00 ± 0.00 | 0.00 ± 0.00 | 1.00 ± 0.00 | 1.00 ± 0.00 |
| Histogram Min | 0.00e+00 ± 0.00e+00 | 0.00 ± 0.00 | 0.00 ± 0.00 | 1.00 ± 0.00 | 1.00 ± 0.00 |
| Histogram Range | 1.80e-01 ± 4.00e-02 | 7.63 ± 5.71 | 32.20 ± 22.05 | 0.88 ± 0.06 | 0.00 ± 0.00 |
| Histogram RMS | 4.00e-02 ± 0.00e+00 | 2.98 ± 2.23 | 13.73 ± 9.93 | 0.90 ± 0.03 | 0.05 ± 0.06 |
| Histogram Skewness | 2.83e+00 ± 4.20e-01 | 6.60 ± 5.69 | 27.33 ± 17.61 | 0.84 ± 0.10 | 0.00 ± 0.00 |
| Histogram STD | 4.00e-02 ± 0.00e+00 | 3.49 ± 2.61 | 16.15 ± 11.68 | 0.90 ± 0.03 | 0.05 ± 0.06 |
| Histogram Uniformity | 1.10e-01 ± 2.00e-02 | 5.86 ± 4.33 | 27.02 ± 21.98 | 0.91 ± 0.03 | 0.06 ± 0.07 |
| Histogram Variance | 0.00e+00 ± 0.00e+00 | 6.85 ± 5.05 | 31.65 ± 26.37 | 0.91 ± 0.03 | 0.06 ± 0.07 |
| Histogram TotalFrequency | 1.81e+03 ± 1.31e+03 | 1.19 ± 1.15 | 12.22 ± 14.41 | 1.00 ± 0.00 | 0.88 ± 0.07 |
| Signal Quantile 0.01 | 3.49e+02 ± 2.08e+02 | 26.22 ± 21.92 | 96.59 ± 170.90 | 0.84 ± 0.05 | 0.02 ± 0.04 |
| Signal Quantile 0.1 | 6.27e+02 ± 1.76e+02 | 5.75 ± 6.26 | 56.44 ± 29.84 | 0.97 ± 0.01 | 0.03 ± 0.05 |
| Signal Quantile 0.2 | 7.32e+02 ± 1.58e+02 | 3.88 ± 4.00 | 44.70 ± 26.89 | 0.98 ± 0.01 | 0.05 ± 0.07 |
| Signal Quantile 0.3 | 8.14e+02 ± 1.50e+02 | 3.32 ± 2.61 | 37.69 ± 24.65 | 0.98 ± 0.01 | 0.10 ± 0.10 |
| Signal Quantile 0.4 | 8.94e+02 ± 1.46e+02 | 3.75 ± 2.95 | 32.31 ± 22.52 | 0.96 ± 0.01 | 0.15 ± 0.12 |
| Signal Quantile 0.5 | 9.85e+02 ± 1.45e+02 | 4.47 ± 4.27 | 26.59 ± 20.57 | 0.93 ± 0.04 | 0.21 ± 0.16 |
| Signal Quantile 0.6 | 1.10e+03 ± 1.52e+02 | 4.37 ± 3.92 | 20.39 ± 18.60 | 0.92 ± 0.02 | 0.29 ± 0.22 |
| Signal Quantile 0.7 | 1.22e+03 ± 1.71e+02 | 3.93 ± 3.55 | 16.02 ± 16.26 | 0.93 ± 0.02 | 0.35 ± 0.23 |
| Signal Quantile 0.8 | 1.37e+03 ± 1.90e+02 | 3.54 ± 2.89 | 14.21 ± 13.13 | 0.95 ± 0.02 | 0.41 ± 0.20 |
| Signal Quantile 0.9 | 1.57e+03 ± 1.97e+02 | 3.43 ± 2.83 | 14.00 ± 12.05 | 0.95 ± 0.03 | 0.37 ± 0.16 |
| Signal Quantile 0.99 | 2.02e+03 ± 2.24e+02 | 3.37 ± 3.84 | 15.38 ± 13.50 | 0.91 ± 0.04 | 0.29 ± 0.14 |

**Table 4** First-Order Statistics (FOS) features for the patients of the OroPharyngeal Cancers (OPC) dataset. In the table, the bin-dependent features computed for 64-bins histogram discretization are presented. The first column shows the mean and standard deviations of the original radiomic features computed on all the patients. The mean and standard deviation of both percentage difference and Intra-class Correlation Coefficient (ICC) are shown for both minimal and maximal entity translations. Features excluded by our method are highlighted in red. Values are shown with scientific format only for the original features. The numbers are approximated to the second decimal significant digit.

| **FIRST-ORDER STATISTICS (64-BINS DISCRETIZATION)** | | | | | |
| --- | --- | --- | --- | --- | --- |
| **Feature name** | **Original value** | **Diff %**  **(min. entity)** | **Diff %**  **(max. entity)** | **ICC_10_** | **ICC_100_** |
| Histogram Entropy | 4.44e+00 ± 2.10e-01 | 1.73 ± 1.16 | 7.45 ± 5.97 | 0.91 ± 0.01 | 0.14 ± 0.19 |
| Histogram Kurtosis | 1.14e+01 ± 3.04e+00 | 11.24 ± 9.32 | 44.35 ± 33.34 | 0.85 ± 0.10 | 0.01 ± 0.02 |
| Histogram Mad | 1.00e-02 ± 0.00e+00 | 1.30 ± 1.13 | 4.77 ± 4.77 | 0.83 ± 0.04 | 0.22 ± 0.25 |
| Histogram Max | 1.00e-01 ± 2.00e-02 | 7.78 ± 6.32 | 30.60 ± 21.06 | 0.85 ± 0.09 | 0.02 ± 0.03 |
| Histogram Mean | 1.00e-02 ± 0.00e+00 | 0.00 ± 0.00 | 0.00 ± 0.00 | 1.00 ± 0.00 | 1.00 ± 0.00 |
| Histogram Median | 0.00e+00 ± 0.00e+00 | 0.00 ± 0.00 | 0.00 ± 0.00 | 1.00 ± 0.00 | 1.00 ± 0.00 |
| Histogram Min | 0.00e+00 ± 0.00e+00 | 0.00 ± 0.00 | 0.00 ± 0.00 | 1.00 ± 0.00 | 1.00 ± 0.00 |
| Histogram Range | 1.00e-01 ± 2.00e-02 | 7.78 ± 6.32 | 30.60 ± 21.06 | 0.85 ± 0.09 | 0.02 ± 0.03 |
| Histogram RMS | 2.00e-02 ± 0.00e+00 | 2.92 ± 2.09 | 13.69 ± 9.84 | 0.91 ± 0.02 | 0.07 ± 0.08 |
| Histogram Skewness | 2.91e+00 ± 4.40e-01 | 6.20 ± 5.32 | 26.30 ± 17.02 | 0.86 ± 0.08 | 0.00 ± 0.01 |
| Histogram STD | 2.00e-02 ± 0.00e+00 | 3.41 ± 2.45 | 16.04 ± 11.54 | 0.91 ± 0.02 | 0.07 ± 0.08 |
| Histogram Uniformity | 6.00e-02 ± 1.00e-02 | 5.76 ± 4.07 | 26.91 ± 21.82 | 0.92 ± 0.02 | 0.07 ± 0.09 |
| Histogram Variance | 0.00e+00 ± 0.00e+00 | 6.71 ± 4.74 | 31.41 ± 26.07 | 0.92 ± 0.02 | 0.07 ± 0.09 |
| Histogram TotalFrequency | 1.81e+03 ± 1.31e+03 | 1.19 ± 1.15 | 12.22 ± 14.41 | 1.00 ± 0.00 | 0.88 ± 0.07 |
| Signal Quantile 0.01 | 3.58e+02 ± 2.12e+02 | 26.36 ± 20.58 | 88.86 ± 113.48 | 0.82 ± 0.04 | 0.02 ± 0.04 |
| Signal Quantile 0.1 | 6.33e+02 ± 1.75e+02 | 5.74 ± 6.84 | 56.39 ± 30.07 | 0.97 ± 0.02 | 0.03 ± 0.05 |
| Signal Quantile 0.2 | 7.37e+02 ± 1.59e+02 | 3.84 ± 3.84 | 44.94 ± 26.95 | 0.98 ± 0.01 | 0.05 ± 0.07 |
| Signal Quantile 0.3 | 8.16e+02 ± 1.49e+02 | 3.30 ± 2.66 | 37.88 ± 24.77 | 0.98 ± 0.01 | 0.10 ± 0.09 |
| Signal Quantile 0.4 | 8.92e+02 ± 1.47e+02 | 3.73 ± 2.94 | 32.45 ± 22.60 | 0.97 ± 0.02 | 0.16 ± 0.12 |
| Signal Quantile 0.5 | 9.83e+02 ± 1.46e+02 | 4.56 ± 4.58 | 26.69 ± 20.65 | 0.92 ± 0.05 | 0.21 ± 0.16 |
| Signal Quantile 0.6 | 1.10e+03 ± 1.53e+02 | 4.34 ± 4.03 | 20.39 ± 18.65 | 0.92 ± 0.02 | 0.29 ± 0.22 |
| Signal Quantile 0.7 | 1.22e+03 ± 1.71e+02 | 4.00 ± 3.56 | 15.95 ± 16.22 | 0.93 ± 0.02 | 0.35 ± 0.23 |
| Signal Quantile 0.8 | 1.37e+03 ± 1.90e+02 | 3.60 ± 2.86 | 14.22 ± 13.12 | 0.95 ± 0.02 | 0.41 ± 0.20 |
| Signal Quantile 0.9 | 1.57e+03 ± 1.99e+02 | 3.41 ± 2.90 | 14.26 ± 11.91 | 0.95 ± 0.03 | 0.37 ± 0.15 |
| Signal Quantile 0.99 | 2.02e+03 ± 2.21e+02 | 3.48 ± 4.20 | 15.72 ± 13.20 | 0.90 ± 0.05 | 0.27 ± 0.15 |

**Table 5** Grey Level Co-occurrence Matrix (GLCM) features for the patients of the OroPharyngeal Cancers (OPC) dataset. In the table, the features computed for 16-bins histogram discretization are presented. The first column shows the mean and standard deviations of the original radiomic features computed on all the patients. The mean and standard deviation of both percentage difference and Intra-class Correlation Coefficient (ICC) are shown for both minimal and maximal entity translations. Features excluded by our method are highlighted in red. Values are shown with scientific format only for the original features. The numbers are approximated to the second decimal significant digit.

| **GREY LEVEL CO-OCCURRENCE MATRIX (16-BINS DISCRETIZATION)** | | | | | |
| --- | --- | --- | --- | --- | --- |
| **Feature name** | **Original value** | **Diff %**  **(min. entity)** | **Diff %**  **(max. entity)** | **ICC_10_** | **ICC_100_** |
| Autocorrelation | 1.40e+01 ± 4.43e+00 | 9.38 ± 7.40 | 37.23 ± 31.71 | 0.94 ± 0.01 | 0.35 ± 0.20 |
| Cluster Prominence | 5.14e+03 ± 2.72e+03 | 18.43 ± 17.27 | 72.27 ± 134.61 | 0.90 ± 0.02 | 0.30 ± 0.24 |
| Cluster Shade | 5.14e+02 ± 2.16e+02 | 13.88 ± 11.98 | 50.08 ± 62.84 | 0.92 ± 0.01 | 0.33 ± 0.21 |
| Cluster Tendency | 5.68e+01 ± 1.71e+01 | 9.29 ± 7.46 | 35.59 ± 30.96 | 0.93 ± 0.01 | 0.36 ± 0.18 |
| Contrast | 2.63e+00 ± 8.20e-01 | 15.34 ± 13.13 | 75.43 ± 70.35 | 0.82 ± 0.03 | 0.02 ± 0.03 |
| Correlation | 1.27e+03 ± 5.05e+02 | 20.51 ± 17.47 | 84.95 ± 206.82 | 0.82 ± 0.06 | 0.22 ± 0.14 |
| Difference Entropy | 1.97e+00 ± 1.80e-01 | 4.36 ± 3.52 | 16.24 ± 11.97 | 0.84 ± 0.04 | 0.05 ± 0.06 |
| Dissimilarity | 1.16e+00 ± 2.10e-01 | 7.94 ± 6.07 | 37.10 ± 29.94 | 0.87 ± 0.04 | 0.02 ± 0.03 |
| Energy | 7.00e-02 ± 2.00e-02 | 13.00 ± 9.25 | 52.34 ± 50.72 | 0.85 ± 0.09 | 0.06 ± 0.07 |
| Entropy | 4.53e+00 ± 4.50e-01 | 3.93 ± 2.93 | 15.80 ± 11.39 | 0.89 ± 0.05 | 0.14 ± 0.19 |
| Homogeneity | 5.90e-01 ± 5.00e-02 | 2.87 ± 2.18 | 12.54 ± 8.87 | 0.89 ± 0.05 | 0.07 ± 0.09 |
| Homogeneity2 | 5.50e-01 ± 6.00e-02 | 3.81 ± 2.81 | 16.58 ± 11.70 | 0.89 ± 0.05 | 0.06 ± 0.08 |
| IMOC1 | -1.40e-01 ± 1.10e-01 | 31.20 ± 35.66 | 74.63 ± 69.73 | 0.62 ± 0.42 | 0.35 ± 0.26 |
| IMOC2 | 6.10e-01 ± 1.30e-01 | 7.72 ± 7.29 | 22.94 ± 17.58 | 0.91 ± 0.02 | 0.42 ± 0.11 |
| Inertia | 2.63e+00 ± 8.20e-01 | 15.34 ± 13.13 | 75.43 ± 70.35 | 0.82 ± 0.03 | 0.02 ± 0.03 |
| Inverse Difference moment | 1.00e+00 ± 0.00e+00 | 0.00 ± 0.00 | 0.00 ± 0.00 | 0.82 ± 0.03 | 0.01 ± 0.03 |
| Inverse Difference moment2 | 1.00e+00 ± 0.00e+00 | 0.03 ± 0.03 | 0.16 ± 0.12 | 0.87 ± 0.04 | 0.02 ± 0.03 |
| Inverse Variance | 4.50e-01 ± 4.00e-02 | 3.56 ± 3.67 | 11.02 ± 8.00 | 0.81 ± 0.08 | 0.09 ± 0.13 |
| Max Probability | 1.70e-01 ± 5.00e-02 | 12.70 ± 9.82 | 49.49 ± 32.73 | 0.84 ± 0.10 | 0.03 ± 0.05 |
| Sum Average | 7.16e+00 ± 1.18e+00 | 4.62 ± 3.43 | 23.06 ± 17.37 | 0.95 ± 0.01 | 0.30 ± 0.18 |
| Sum Entropy | 3.11e+00 ± 2.50e-01 | 3.09 ± 2.64 | 12.45 ± 9.67 | 0.89 ± 0.04 | 0.22 ± 0.26 |

**Table 6** Grey Level Co-occurrence Matrix (GLCM) features for the patients of the OroPharyngeal Cancers (OPC) dataset. In the table, the features computed for 32-bins histogram discretization are presented. The first column shows the mean and standard deviations of the original radiomic features computed on all the patients. The mean and standard deviation of both percentage difference and Intra-class Correlation Coefficient (ICC) are shown for both minimal and maximal entity translations. Features excluded by our method are highlighted in red. Values are shown with scientific format only for the original features. The numbers are approximated to the second decimal significant digit.

| **GREY LEVEL CO-OCCURRENCE MATRIX (32-BINS DISCRETIZATION)** | | | | | |
| --- | --- | --- | --- | --- | --- |
| **Feature name** | **Original value** | **Diff %**  **(min. entity)** | **Diff %**  **(max. entity)** | **ICC_10_** | **ICC_100_** |
| Autocorrelation | 6.32e+01 ± 1.89e+01 | 8.76 ± 6.83 | 35.35 ± 29.38 | 0.94 ± 0.01 | 0.35 ± 0.20 |
| Cluster Prominence | 1.03e+05 ± 5.19e+04 | 17.15 ± 15.76 | 66.58 ± 114.58 | 0.91 ± 0.02 | 0.30 ± 0.24 |
| Cluster Shade | 4.95e+03 ± 1.97e+03 | 12.92 ± 10.95 | 47.10 ± 55.36 | 0.92 ± 0.01 | 0.34 ± 0.20 |
| Cluster Tendency | 2.61e+02 ± 7.40e+01 | 8.63 ± 6.83 | 33.70 ± 28.55 | 0.93 ± 0.01 | 0.35 ± 0.18 |
| Contrast | 1.01e+01 ± 3.37e+00 | 15.76 ± 13.80 | 80.70 ± 77.96 | 0.83 ± 0.04 | 0.01 ± 0.03 |
| Correlation | 2.23e+04 ± 8.97e+03 | 20.31 ± 17.24 | 85.26 ± 187.97 | 0.83 ± 0.06 | 0.21 ± 0.13 |
| Difference Entropy | 2.77e+00 ± 2.20e-01 | 3.55 ± 3.10 | 13.53 ± 9.85 | 0.84 ± 0.05 | 0.06 ± 0.08 |
| Dissimilarity | 2.33e+00 ± 4.30e-01 | 8.02 ± 6.42 | 37.12 ± 31.20 | 0.87 ± 0.04 | 0.02 ± 0.03 |
| Energy | 2.00e-02 ± 1.00e-02 | 12.89 ± 9.60 | 51.35 ± 47.68 | 0.87 ± 0.07 | 0.20 ± 0.15 |
| Entropy | 6.18e+00 ± 6.30e-01 | 2.80 ± 2.25 | 11.09 ± 7.59 | 0.95 ± 0.02 | 0.46 ± 0.17 |
| Homogeneity | 4.40e-01 ± 5.00e-02 | 3.82 ± 3.05 | 15.50 ± 11.04 | 0.89 ± 0.06 | 0.07 ± 0.09 |
| Homogeneity2 | 3.70e-01 ± 6.00e-02 | 5.51 ± 4.39 | 21.81 ± 15.32 | 0.88 ± 0.06 | 0.07 ± 0.10 |
| IMOC1 | -1.60e-01 ± 1.20e-01 | 21.49 ± 21.14 | 57.27 ± 52.48 | 0.79 ± 0.08 | 0.66 ± 0.05 |
| IMOC2 | 7.40e-01 ± 1.30e-01 | 4.28 ± 4.00 | 14.71 ± 11.67 | 0.96 ± 0.01 | 0.63 ± 0.09 |
| Inertia | 1.01e+01 ± 3.37e+00 | 15.76 ± 13.80 | 80.70 ± 77.96 | 0.83 ± 0.04 | 0.01 ± 0.03 |
| Inverse Difference moment | 1.00e+00 ± 0.00e+00 | 0.00 ± 0.00 | 0.00 ± 0.00 | 0.83 ± 0.04 | 0.01 ± 0.03 |
| Inverse Difference moment2 | 1.00e+00 ± 0.00e+00 | 0.02 ± 0.01 | 0.08 ± 0.06 | 0.87 ± 0.04 | 0.02 ± 0.03 |
| Inverse Variance | 3.60e-01 ± 5.00e-02 | 5.00 ± 4.30 | 20.12 ± 13.51 | 0.85 ± 0.07 | 0.02 ± 0.03 |
| Max Probability | 6.00e-02 ± 3.00e-02 | 14.06 ± 11.71 | 54.20 ± 34.32 | 0.85 ± 0.12 | 0.07 ± 0.07 |
| Sum Average | 1.53e+01 ± 2.36e+00 | 4.34 ± 3.17 | 21.69 ± 16.32 | 0.95 ± 0.01 | 0.30 ± 0.19 |
| Sum Entropy | 4.06e+00 ± 2.80e-01 | 2.36 ± 1.98 | 9.71 ± 7.41 | 0.91 ± 0.04 | 0.26 ± 0.27 |

**Table 7** Grey Level Co-occurrence Matrix (GLCM) features for the patients of the OroPharyngeal Cancers (OPC) dataset. In the table, the features computed for 64-bins histogram discretization are presented. The first column shows the mean and standard deviations of the original radiomic features computed on all the patients. The mean and standard deviation of both percentage difference and Intra-class Correlation Coefficient (ICC) are shown for both minimal and maximal entity translations. Features excluded by our method are highlighted in red. Values are shown with scientific format only for the original features. The numbers are approximated to the second decimal significant digit.

| **GREY LEVEL CO-OCCURRENCE MATRIX (64-BINS DISCRETIZATION)** | | | | | |
| --- | --- | --- | --- | --- | --- |
| **Feature name** | **Original value** | **Diff %**  **(min. entity)** | **Diff %**  **(max. entity)** | **ICC_10_** | **ICC_100_** |
| Autocorrelation | 2.68e+02 ± 7.81e+01 | 8.52 ± 6.60 | 34.39 ± 28.25 | 0.94 ± 0.01 | 0.35 ± 0.19 |
| Cluster Prominence | 1.82e+06 ± 9.01e+05 | 16.76 ± 15.41 | 64.13 ± 107.15 | 0.91 ± 0.02 | 0.31 ± 0.23 |
| Cluster Shade | 4.31e+04 ± 1.67e+04 | 12.57 ± 10.65 | 45.73 ± 52.36 | 0.92 ± 0.01 | 0.34 ± 0.20 |
| Cluster Tendency | 1.11e+03 ± 3.06e+02 | 8.38 ± 6.60 | 32.76 ± 27.50 | 0.93 ± 0.01 | 0.35 ± 0.18 |
| Contrast | 4.01e+01 ± 1.37e+01 | 15.94 ± 13.73 | 83.09 ± 81.37 | 0.83 ± 0.04 | 0.01 ± 0.03 |
| Correlation | 3.70e+05 ± 1.51e+05 | 19.03 ± 16.04 | 80.83 ± 170.48 | 0.85 ± 0.05 | 0.23 ± 0.13 |
| Difference Entropy | 3.65e+00 ± 2.50e-01 | 2.86 ± 2.30 | 11.12 ± 7.87 | 0.86 ± 0.05 | 0.10 ± 0.13 |
| Dissimilarity | 4.66e+00 ± 8.90e-01 | 7.93 ± 6.34 | 37.59 ± 31.97 | 0.87 ± 0.04 | 0.02 ± 0.03 |
| Energy | 1.00e-02 ± 1.00e-02 | 10.71 ± 8.23 | 42.20 ± 36.81 | 0.97 ± 0.01 | 0.82 ± 0.06 |
| Entropy | 7.62e+00 ± 9.50e-01 | 1.80 ± 1.35 | 6.85 ± 4.85 | 0.99 ± 0.01 | 0.81 ± 0.08 |
| Homogeneity | 3.10e-01 ± 4.00e-02 | 4.50 ± 3.39 | 18.25 ± 12.33 | 0.91 ± 0.04 | 0.08 ± 0.06 |
| Homogeneity2 | 2.20e-01 ± 5.00e-02 | 6.91 ± 5.13 | 26.59 ± 17.25 | 0.91 ± 0.03 | 0.08 ± 0.06 |
| IMOC1 | -1.60e-01 ± 1.10e-01 | 33.77 ± 86.13 | 75.10 ± 117.80 | 0.61 ± 0.21 | 0.36 ± 0.16 |
| IMOC2 | 8.70e-01 ± 1.00e-01 | 1.85 ± 2.08 | 7.18 ± 6.63 | 0.98 ± 0.00 | 0.70 ± 0.06 |
| Inertia | 4.01e+01 ± 1.37e+01 | 15.94 ± 13.73 | 83.09 ± 81.37 | 0.83 ± 0.04 | 0.01 ± 0.03 |
| Inverse Difference moment | 1.00e+00 ± 0.00e+00 | 0.00 ± 0.00 | 0.00 ± 0.00 | 0.74 ± 0.05 | 0.01 ± 0.02 |
| Inverse Difference moment2 | 1.00e+00 ± 0.00e+00 | 0.01 ± 0.01 | 0.04 ± 0.03 | 0.87 ± 0.04 | 0.02 ± 0.03 |
| Inverse Variance | 2.30e-01 ± 5.00e-02 | 7.34 ± 5.35 | 26.43 ± 17.10 | 0.86 ± 0.04 | 0.03 ± 0.02 |
| Max Probability | 3.00e-02 ± 2.00e-02 | 12.21 ± 9.77 | 45.73 ± 34.52 | 0.92 ± 0.04 | 0.55 ± 0.10 |
| Sum Average | 3.16e+01 ± 4.72e+00 | 4.21 ± 3.07 | 20.97 ± 15.79 | 0.95 ± 0.01 | 0.30 ± 0.19 |
| Sum Entropy | 4.99e+00 ± 3.40e-01 | 1.98 ± 1.71 | 7.73 ± 5.83 | 0.93 ± 0.03 | 0.43 ± 0.22 |

**Table 8** Grey Level Run Length Matrix (GLRLM) features for the patients of the OroPharyngeal Cancers (OPC) dataset. In the table, the features computed for 16-bins histogram discretization are presented. The first column shows the mean and standard deviations of the original radiomic features computed on all the patients. The mean and standard deviation of both percentage difference and Intra-class Correlation Coefficient (ICC) are shown for both minimal and maximal entity translations. Features excluded by our method are highlighted in red. Values are shown with scientific format only for the original features. The numbers are approximated to the second decimal significant digit.

| **GREY LEVEL RUN LENGTH MATRIX (16-BINS DISCRETIZATION)** | | | | | |
| --- | --- | --- | --- | --- | --- |
| **Feature name** | **Original value** | **Diff %**  **(min. entity)** | **Diff %**  **(max. entity)** | **ICC_10_** | **ICC_100_** |
| Short Run Emphasis | 9.80e-01 ± 1.00e-02 | 0.27 ± 0.35 | 1.31 ± 1.08 | 0.95 ± 0.03 | 0.32 ± 0.27 |
| Long Run Emphasis | 1.10e+00 ± 7.00e-02 | 1.07 ± 1.30 | 5.14 ± 4.20 | 0.96 ± 0.03 | 0.27 ± 0.24 |
| Grey Level Non-Uniformity | 2.55e+02 ± 1.82e+02 | 4.44 ± 3.66 | 19.73 ± 14.92 | 1.00 ± 0.00 | 0.93 ± 0.03 |
| Run Length Non-Uniformity | 1.31e+03 ± 9.33e+02 | 2.41 ± 1.86 | 13.38 ± 12.18 | 1.00 ± 0.00 | 0.91 ± 0.06 |
| Run Percentage | 7.90e-01 ± 6.00e-02 | 1.77 ± 1.36 | 8.46 ± 5.46 | 0.95 ± 0.01 | 0.29 ± 0.19 |
| Low Grey Level Run Emphasis | 8.00e-02 ± 5.00e-02 | 12.54 ± 10.59 | 232.36 ± 246.41 | 0.97 ± 0.01 | 0.09 ± 0.11 |
| high Grey Level Run Emphasis | 2.62e+01 ± 6.44e+00 | 6.05 ± 4.96 | 28.35 ± 22.97 | 0.96 ± 0.02 | 0.27 ± 0.24 |
| Short Run Low Grey Level Emphasis | 8.00e-02 ± 5.00e-02 | 12.90 ± 10.79 | 237.29 ± 250.54 | 0.97 ± 0.01 | 0.09 ± 0.12 |
| Short Run High Grey Level Emphasis | 2.58e+01 ± 6.23e+00 | 6.12 ± 5.00 | 28.27 ± 22.99 | 0.96 ± 0.02 | 0.26 ± 0.23 |
| Long Run Low Grey Level Emphasis | 9.00e-02 ± 5.00e-02 | 11.43 ± 9.98 | 214.20 ± 232.90 | 0.96 ± 0.01 | 0.08 ± 0.10 |
| Long Run High Grey Level Emphasis | 2.82e+01 ± 7.60e+00 | 6.10 ± 4.79 | 29.45 ± 22.52 | 0.97 ± 0.01 | 0.31 ± 0.24 |

**Table 9** Grey Level Run Length Matrix (GLRLM) features for the patients of the OroPharyngeal Cancers (OPC) dataset. In the table, the features computed for 32-bins histogram discretization are presented. The first column shows the mean and standard deviations of the original radiomic features computed on all the patients. The mean and standard deviation of both percentage difference and Intra-class Correlation Coefficient (ICC) are shown for both minimal and maximal entity translations. Features excluded by our method are highlighted in red. Values are shown with scientific format only for the original features. The numbers are approximated to the second decimal significant digit.

| **GREY LEVEL RUN LENGTH MATRIX (32-BINS DISCRETIZATION)** | | | | | |
| --- | --- | --- | --- | --- | --- |
| **Feature name** | **Original value** | **Diff %**  **(min. entity)** | **Diff %**  **(max. entity)** | **ICC_10_** | **ICC_100_** |
| Short Run Emphasis | 9.80e-01 ± 1.00e-02 | 0.14 ± 0.15 | 1.19 ± 0.86 | 0.98 ± 0.01 | 0.25 ± 0.17 |
| Long Run Emphasis | 1.11e+00 ± 6.00e-02 | 0.77 ± 0.81 | 5.41 ± 3.96 | 0.98 ± 0.01 | 0.29 ± 0.22 |
| Grey Level Non-Uniformity | 1.59e+02 ± 1.15e+02 | 5.31 ± 4.08 | 24.04 ± 17.72 | 1.00 ± 0.00 | 0.88 ± 0.04 |
| Run Length Non-Uniformity | 1.49e+03 ± 1.06e+03 | 1.62 ± 1.41 | 11.80 ± 12.54 | 1.00 ± 0.00 | 0.91 ± 0.06 |
| Run Percentage | 8.90e-01 ± 3.00e-02 | 1.02 ± 0.76 | 4.58 ± 2.76 | 0.94 ± 0.02 | 0.26 ± 0.12 |
| Low Grey Level Run Emphasis | 3.00e-02 ± 3.00e-02 | 18.44 ± 17.43 | 457.88 ± 542.11 | 0.96 ± 0.01 | 0.08 ± 0.10 |
| High Grey Level Run Emphasis | 9.26e+01 ± 2.31e+01 | 6.24 ± 5.11 | 29.50 ± 24.07 | 0.96 ± 0.02 | 0.28 ± 0.23 |
| Short Run Low Grey Level Emphasis | 3.00e-02 ± 3.00e-02 | 18.81 ± 17.83 | 464.23 ± 546.99 | 0.96 ± 0.01 | 0.08 ± 0.10 |
| Short Run High Grey Level Emphasis | 9.12e+01 ± 2.24e+01 | 6.28 ± 5.12 | 29.35 ± 24.19 | 0.95 ± 0.02 | 0.27 ± 0.23 |
| Long Run Low Grey Level Emphasis | 3.00e-02 ± 3.00e-02 | 16.66 ± 15.88 | 427.34 ± 517.39 | 0.96 ± 0.01 | 0.07 ± 0.09 |
| Long Run High Grey Level Emphasis | 9.98e+01 ± 2.70e+01 | 6.13 ± 5.09 | 30.69 ± 23.56 | 0.96 ± 0.01 | 0.31 ± 0.22 |

**Table 10** Grey Level Run Length Matrix (GLRLM) features for the patients of the OroPharyngeal Cancers (OPC) dataset. In the table, the features computed for 64-bins histogram discretization are presented. The first column shows the mean and standard deviations of the original radiomic features computed on all the patients. The mean and standard deviation of both percentage difference and Intra-class Correlation Coefficient (ICC) are shown for both minimal and maximal entity translations. Features excluded by our method are highlighted in red. Values are shown with scientific format only for the original features. The numbers are approximated to the second decimal significant digit.

| **GREY LEVEL RUN LENGTH MATRIX (64-BINS DISCRETIZATION)** | | | | | |
| --- | --- | --- | --- | --- | --- |
| **Feature name** | **Original value** | **Diff %**  **(min. entity)** | **Diff %**  **(max. entity)** | **ICC_10_** | **ICC_100_** |
| Short Run Emphasis | 9.40e-01 ± 1.00e-02 | 0.47 ± 0.44 | 1.81 ± 1.18 | 0.89 ± 0.05 | 0.22 ± 0.24 |
| Long Run Emphasis | 1.30e+00 ± 8.00e-02 | 1.68 ± 1.72 | 7.88 ± 5.10 | 0.94 ± 0.03 | 0.18 ± 0.20 |
| Grey Level Non-Uniformity | 8.93e+01 ± 6.57e+01 | 5.54 ± 4.19 | 26.36 ± 19.10 | 0.99 ± 0.00 | 0.84 ± 0.05 |
| Run Length Non-Uniformity | 1.43e+03 ± 1.02e+03 | 1.87 ± 1.80 | 11.68 ± 12.56 | 1.00 ± 0.00 | 0.91 ± 0.06 |
| Run Percentage | 9.40e-01 ± 2.00e-02 | 0.56 ± 0.43 | 2.37 ± 1.46 | 0.94 ± 0.02 | 0.25 ± 0.19 |
| Low Grey Level Run Emphasis | 1.00e-02 ± 1.00e-02 | 30.04 ± 31.71 | 847.13 ± 1075.13 | 0.96 ± 0.00 | 0.07 ± 0.09 |
| high Grey Level Run Emphasis | 3.47e+02 ± 8.74e+01 | 6.49 ± 5.25 | 30.28 ± 24.62 | 0.95 ± 0.02 | 0.29 ± 0.22 |
| Short Run Low Grey Level Emphasis | 1.00e-02 ± 1.00e-02 | 31.31 ± 33.03 | 860.99 ± 1084.31 | 0.96 ± 0.00 | 0.07 ± 0.09 |
| Short Run High Grey Level Emphasis | 3.30e+02 ± 8.17e+01 | 6.66 ± 5.33 | 30.09 ± 24.77 | 0.95 ± 0.02 | 0.28 ± 0.21 |
| Long Run Low Grey Level Emphasis | 1.00e-02 ± 2.00e-02 | 25.61 ± 26.97 | 782.58 ± 1017.10 | 0.96 ± 0.00 | 0.06 ± 0.08 |
| Long Run High Grey Level Emphasis | 4.30e+02 ± 1.21e+02 | 6.41 ± 4.96 | 32.33 ± 24.04 | 0.96 ± 0.01 | 0.32 ± 0.23 |

- 1. **Soft-tissues sarcoma dataset**

**Table 11** First-Order Statistics (FOS) features for the patients of the Soft-tissues Sarcomas (STS) dataset. In the table, the bin-independent features are presented. The first column shows the mean and standard deviations of the original radiomic features computed on all the patients. The mean and standard deviation of both percentage difference and Intra-class Correlation Coefficient (ICC) are shown for both minimal and maximal entity translations. Features excluded by our method are highlighted in red. Values are shown with scientific format only for the original features (and for the errors of signal minimum). The numbers are approximated to the second decimal significant digit.

| **FIRST-ORDER STATISTICS (BIN-INDEPENDENT)** | | | | | |
| --- | --- | --- | --- | --- | --- |
| **Feature name** | **Original value** | **Diff %**  **(min. entity)** | **Diff %**  **(max. entity)** | **ICC_10_** | **ICC_100_** |
| Signal Energy | 1.34e+11 ± 1.08e+11 | 10.41 ± 5.58 | 93.68 ± 8.39 | 0.98 ± 0.00 | 0.03 ± 0.04 |
| Signal Kurtosis | 3.17e+00 ± 1.03e+00 | 6.91 ± 7.34 | 131.84 ± 252.96 | 0.94 ± 0.02 | 0.01 ± 0.02 |
| Signal Mad | 4.37e+02 ± 1.23e+02 | 8.88 ± 8.89 | 51.09 ± 21.67 | 0.93 ± 0.01 | 0.06 ± 0.04 |
| Signal Max | 3.41e+03 ± 3.72e+02 | 1.46 ± 2.74 | 39.40 ± 19.08 | 0.96 ± 0.03 | 0.08 ± 0.05 |
| Signal Mean | 1.48e+03 ± 3.13e+02 | 6.27 ± 3.24 | 74.11 ± 11.77 | 0.94 ± 0.01 | 0.02 ± 0.02 |
| Signal Median | 1.46e+03 ± 3.97e+02 | 6.07 ± 4.15 | 78.20 ± 10.94 | 0.95 ± 0.01 | 0.02 ± 0.02 |
| Signal Min | 6.93e+00 ± 1.76e+01 | 3.02e+11± 1.80e+12 | 2.41e+11 ± 1.48e+12 | 0.09 ± 0.03 | 0.00 ± 0.00 |
| Signal Range | 3.40e+03 ± 3.79e+02 | 1.62 ± 2.70 | 39.27 ± 19.11 | 0.96 ± 0.03 | 0.08 ± 0.05 |
| Signal RMS | 1.58e+03 ± 3.23e+02 | 4.62 ± 2.39 | 68.87 ± 14.13 | 0.97 ± 0.00 | 0.03 ± 0.01 |
| Signal Skewness | 2.50e-01 ± 5.90e-01 | 508.78 ± 1220.18 | 4592.69 ± 13171.08 | 0.93 ± 0.02 | 0.04 ± 0.03 |
| Signal STD | 5.35e+02 ± 1.23e+02 | 9.14 ± 8.41 | 48.88 ± 21.68 | 0.90 ± 0.02 | 0.06 ± 0.04 |
| Signal Variance | 3.01e+05 ± 1.47e+05 | 19.81 ± 19.23 | 75.44 ± 47.70 | 0.90 ± 0.02 | 0.05 ± 0.05 |

**Table 12** First-Order Statistics (FOS) features for the patients of the Soft-tissues Sarcomas (STS) dataset. In the table, the bin-dependent features computed for 16-bins histogram discretization are presented. The first column shows the mean and standard deviations of the original radiomic features computed on all the patients. The mean and standard deviation of both percentage difference and Intra-class Correlation Coefficient (ICC) are shown for both minimal and maximal entity translations. Features excluded by our method are highlighted in red. Values are shown with scientific format only for the original features. The numbers are approximated to the second decimal significant digit.

| **FIRST-ORDER STATISTICS (16-BINS DISCRETIZATION)** | | | | | |
| --- | --- | --- | --- | --- | --- |
| **Feature name** | **Original value** | **Diff %**  **(min. entity)** | **Diff %**  **(max. entity)** | **ICC_10_** | **ICC_100_** |
| Histogram Entropy | 2.99e+00 ± 2.60e-01 | 3.75 ± 2.65 | 39.86 ± 18.33 | 0.88 ± 0.02 | 0.05 ± 0.03 |
| Histogram Kurtosis | 6.74e+00 ± 3.73e+00 | 7.90 ± 5.35 | 189.56 ± 175.94 | 0.98 ± 0.01 | 0.08 ± 0.03 |
| Histogram Mad | 4.00e-02 ± 0.00e+00 | 3.57 ± 1.94 | 20.68 ± 9.12 | 0.79 ± 0.03 | 0.02 ± 0.02 |
| Histogram Max | 2.30e-01 ± 7.00e-02 | 6.33 ± 4.74 | 125.78 ± 98.01 | 0.95 ± 0.01 | 0.06 ± 0.05 |
| Histogram Mean | 3.00e-02 ± 0.00e+00 | 0.00 ± 0.00 | 0.00 ± 0.00 | 1.00 ± 0.00 | 1.00 ± 0.00 |
| Histogram Median | 0.00e+00 ± 0.00e+00 | 0.00 ± 0.00 | 0.00 ± 0.00 | 0.93 ± 0.13 | 0.00 ± 0.00 |
| Histogram Min | 0.00e+00 ± 0.00e+00 | 0.00 ± 0.00 | 0.00 ± 0.00 | 1.00 ± 0.00 | 1.00 ± 0.00 |
| Histogram Range | 2.30e-01 ± 7.00e-02 | 6.33 ± 4.74 | 125.78 ± 98.01 | 0.95 ± 0.01 | 0.06 ± 0.05 |
| Histogram RMS | 7.00e-02 ± 1.00e-02 | 4.01 ± 2.91 | 54.60 ± 30.91 | 0.90 ± 0.02 | 0.05 ± 0.04 |
| Histogram Skewness | 2.10e+00 ± 6.60e-01 | 6.08 ± 4.48 | 86.06 ± 66.64 | 0.97 ± 0.01 | 0.10 ± 0.04 |
| Histogram STD | 6.00e-02 ± 1.00e-02 | 5.09 ± 3.63 | 66.56 ± 38.13 | 0.90 ± 0.02 | 0.06 ± 0.04 |
| Histogram Uniformity | 1.50e-01 ± 3.00e-02 | 7.78 ± 5.53 | 148.38 ± 101.28 | 0.89 ± 0.02 | 0.04 ± 0.03 |
| Histogram Variance | 0.00e+00 ± 0.00e+00 | 9.79 ± 6.82 | 191.68 ± 137.39 | 0.89 ± 0.02 | 0.04 ± 0.03 |
| Histogram TotalFrequency | 4.90e+04 ± 3.61e+04 | 1.69 ± 1.77 | 52.25 ± 24.51 | 1.00 ± 0.00 | 0.26 ± 0.26 |
| Signal Quantile 0.01 | 3.39e+02 ± 1.48e+02 | 59.16 ± 17.90 | 97.79 ± 1.39 | 0.34 ± 0.04 | 0.00 ± 0.00 |
| Signal Quantile 0.1 | 8.17e+02 ± 2.02e+02 | 20.63 ± 15.54 | 91.74 ± 5.07 | 0.59 ± 0.05 | 0.00 ± 0.00 |
| Signal Quantile 0.2 | 1.00e+03 ± 2.36e+02 | 10.82 ± 7.72 | 86.81 ± 7.46 | 0.80 ± 0.04 | 0.01 ± 0.01 |
| Signal Quantile 0.3 | 1.15e+03 ± 2.75e+02 | 8.42 ± 5.78 | 83.13 ± 8.83 | 0.87 ± 0.03 | 0.01 ± 0.01 |
| Signal Quantile 0.4 | 1.30e+03 ± 3.30e+02 | 7.18 ± 4.98 | 80.20 ± 9.83 | 0.90 ± 0.02 | 0.01 ± 0.01 |
| Signal Quantile 0.5 | 1.46e+03 ± 3.93e+02 | 6.06 ± 4.09 | 77.65 ± 10.75 | 0.95 ± 0.01 | 0.02 ± 0.02 |
| Signal Quantile 0.6 | 1.61e+03 ± 4.22e+02 | 4.76 ± 3.13 | 74.92 ± 11.76 | 0.97 ± 0.00 | 0.03 ± 0.02 |
| Signal Quantile 0.7 | 1.77e+03 ± 4.43e+02 | 3.40 ± 1.94 | 71.75 ± 12.98 | 0.99 ± 0.00 | 0.03 ± 0.02 |
| Signal Quantile 0.8 | 1.95e+03 ± 4.31e+02 | 3.06 ± 2.32 | 68.01 ± 14.45 | 0.99 ± 0.00 | 0.03 ± 0.02 |
| Signal Quantile 0.9 | 2.20e+03 ± 4.03e+02 | 2.44 ± 2.73 | 63.06 ± 16.52 | 0.99 ± 0.00 | 0.03 ± 0.02 |
| Signal Quantile 0.99 | 2.74e+03 ± 3.27e+02 | 1.82 ± 3.55 | 52.75 ± 17.07 | 0.96 ± 0.03 | 0.04 ± 0.03 |

**Table 13** First-Order Statistics (FOS) features for the patients of the Soft-tissues Sarcomas (STS) dataset. In the table, the bin-dependent features computed for 32-bins histogram discretization are presented. The first column shows the mean and standard deviations of the original radiomic features computed on all the patients. The mean and standard deviation of both percentage difference and Intra-class Correlation Coefficient (ICC) are shown for both minimal and maximal entity translations. Features excluded by our method are highlighted in red. Values are shown with scientific format only for the original features. The numbers are approximated to the second decimal significant digit.

| **FIRST-ORDER STATISTICS (32-BINS DISCRETIZATION)** | | | | | |
| --- | --- | --- | --- | --- | --- |
| **Feature name** | **Original value** | **Diff %**  **(min. entity)** | **Diff %**  **(max. entity)** | **ICC_10_** | **ICC_100_** |
| Histogram Entropy | 3.97e+00 ± 2.60e-01 | 2.93 ± 1.96 | 30.08 ± 14.08 | 0.88 ± 0.02 | 0.05 ± 0.03 |
| Histogram Kurtosis | 7.13e+00 ± 4.00e+00 | 8.00 ± 5.86 | 182.78 ± 174.32 | 0.98 ± 0.01 | 0.09 ± 0.05 |
| Histogram Mad | 2.00e-02 ± 0.00e+00 | 3.78 ± 1.86 | 20.32 ± 9.08 | 0.79 ± 0.03 | 0.02 ± 0.02 |
| Histogram Max | 1.30e-01 ± 4.00e-02 | 6.84 ± 5.27 | 125.47 ± 106.14 | 0.96 ± 0.01 | 0.06 ± 0.06 |
| Histogram Mean | 2.00e-02 ± 0.00e+00 | 0.00 ± 0.00 | 0.00 ± 0.00 | 1.00 ± 0.00 | 1.00 ± 0.00 |
| Histogram Median | 0.00e+00 ± 0.00e+00 | 0.00 ± 0.00 | 0.00 ± 0.00 | 1.00 ± 0.00 | 0.00 ± 0.00 |
| Histogram Min | 0.00e+00 ± 0.00e+00 | 0.00 ± 0.00 | 0.00 ± 0.00 | 1.00 ± 0.00 | 1.00 ± 0.00 |
| Histogram Range | 1.30e-01 ± 4.00e-02 | 6.84 ± 5.27 | 125.47 ± 106.14 | 0.96 ± 0.01 | 0.06 ± 0.06 |
| Histogram RMS | 3.00e-02 ± 0.00e+00 | 4.13 ± 2.93 | 54.42 ± 31.49 | 0.90 ± 0.02 | 0.06 ± 0.04 |
| Histogram Skewness | 2.16e+00 ± 6.80e-01 | 6.08 ± 4.53 | 82.78 ± 65.95 | 0.97 ± 0.01 | 0.10 ± 0.05 |
| Histogram STD | 3.00e-02 ± 0.00e+00 | 5.21 ± 3.64 | 66.06 ± 38.72 | 0.90 ± 0.02 | 0.06 ± 0.04 |
| Histogram Uniformity | 8.00e-02 ± 2.00e-02 | 8.00 ± 5.56 | 148.17 ± 103.50 | 0.90 ± 0.02 | 0.05 ± 0.03 |
| Histogram Variance | 0.00e+00 ± 0.00e+00 | 10.02 ± 6.82 | 190.46 ± 139.79 | 0.90 ± 0.02 | 0.05 ± 0.03 |
| Histogram TotalFrequency | 4.90e+04 ± 3.61e+04 | 1.69 ± 1.77 | 52.25 ± 24.51 | 1.00 ± 0.00 | 0.26 ± 0.26 |
| Signal Quantile 0.01 | 3.59e+02 ± 1.58e+02 | 58.83 ± 17.13 | 97.84 ± 1.59 | 0.33 ± 0.03 | 0.00 ± 0.00 |
| Signal Quantile 0.1 | 8.33e+02 ± 1.92e+02 | 20.41 ± 15.84 | 91.81 ± 5.50 | 0.58 ± 0.06 | 0.00 ± 0.00 |
| Signal Quantile 0.2 | 1.01e+03 ± 2.35e+02 | 10.72 ± 8.02 | 87.02 ± 7.74 | 0.80 ± 0.04 | 0.01 ± 0.01 |
| Signal Quantile 0.3 | 1.15e+03 ± 2.76e+02 | 8.08 ± 5.68 | 83.41 ± 9.07 | 0.87 ± 0.03 | 0.01 ± 0.01 |
| Signal Quantile 0.4 | 1.30e+03 ± 3.31e+02 | 7.11 ± 5.01 | 80.46 ± 10.06 | 0.90 ± 0.02 | 0.01 ± 0.01 |
| Signal Quantile 0.5 | 1.46e+03 ± 3.95e+02 | 6.04 ± 4.10 | 77.96 ± 10.82 | 0.95 ± 0.01 | 0.02 ± 0.02 |
| Signal Quantile 0.6 | 1.61e+03 ± 4.28e+02 | 4.75 ± 3.15 | 75.37 ± 11.82 | 0.97 ± 0.00 | 0.03 ± 0.02 |
| Signal Quantile 0.7 | 1.77e+03 ± 4.48e+02 | 3.56 ± 2.04 | 72.42 ± 13.08 | 0.99 ± 0.00 | 0.03 ± 0.02 |
| Signal Quantile 0.8 | 1.94e+03 ± 4.37e+02 | 2.90 ± 2.13 | 68.62 ± 14.81 | 0.99 ± 0.00 | 0.03 ± 0.02 |
| Signal Quantile 0.9 | 2.19e+03 ± 4.02e+02 | 2.34 ± 2.56 | 63.73 ± 16.85 | 0.99 ± 0.00 | 0.03 ± 0.02 |
| Signal Quantile 0.99 | 2.71e+03 ± 3.19e+02 | 1.83 ± 3.49 | 53.14 ± 17.45 | 0.96 ± 0.03 | 0.04 ± 0.03 |

**Table 14** First-Order Statistics (FOS) features for the patients of the Soft-tissues Sarcomas (STS) dataset. In the table, the bin-dependent features computed for 64-bins histogram discretization are presented. The first column shows the mean and standard deviations of the original radiomic features computed on all the patients. The mean and standard deviation of both percentage difference and Intra-class Correlation Coefficient (ICC) are shown for both minimal and maximal entity translations. Features excluded by our method are highlighted in red. Values are shown with scientific format only for the original features. The numbers are approximated to the second decimal significant digit.

| **FIRST-ORDER STATISTICS (64-BINS DISCRETIZATION)** | | | | | |
| --- | --- | --- | --- | --- | --- |
| **Feature name** | **Original value** | **Diff %**  **(min. entity)** | **Diff %**  **(max. entity)** | **ICC_10_** | **ICC_100_** |
| Histogram Entropy | 4.96e+00 ± 2.60e-01 | 2.36 ± 1.56 | 24.14 ± 11.34 | 0.88 ± 0.02 | 0.05 ± 0.03 |
| Histogram Kurtosis | 7.30e+00 ± 4.24e+00 | 7.92 ± 5.89 | 186.52 ± 177.02 | 0.98 ± 0.01 | 0.08 ± 0.04 |
| Histogram Mad | 1.00e-02 ± 0.00e+00 | 3.76 ± 1.85 | 20.33 ± 9.07 | 0.79 ± 0.03 | 0.02 ± 0.02 |
| Histogram Max | 6.00e-02 ± 2.00e-02 | 6.77 ± 5.23 | 130.86 ± 106.87 | 0.96 ± 0.01 | 0.05 ± 0.06 |
| Histogram Mean | 1.00e-02 ± 0.00e+00 | 0.00 ± 0.00 | 0.00 ± 0.00 | 1.00 ± 0.00 | 1.00 ± 0.00 |
| Histogram Median | 0.00e+00 ± 0.00e+00 | 0.00 ± 0.00 | 0.00 ± 0.00 | 1.00 ± 0.00 | 0.00 ± 0.00 |
| Histogram Min | 0.00e+00 ± 0.00e+00 | 0.00 ± 0.00 | 0.00 ± 0.00 | 1.00 ± 0.00 | 1.00 ± 0.00 |
| Histogram Range | 6.00e-02 ± 2.00e-02 | 6.77 ± 5.23 | 130.86 ± 106.87 | 0.96 ± 0.01 | 0.05 ± 0.06 |
| Histogram RMS | 2.00e-02 ± 0.00e+00 | 4.15 ± 2.93 | 54.70 ± 31.77 | 0.90 ± 0.02 | 0.06 ± 0.04 |
| Histogram Skewness | 2.19e+00 ± 7.00e-01 | 6.03 ± 4.49 | 83.41 ± 66.45 | 0.97 ± 0.01 | 0.10 ± 0.05 |
| Histogram STD | 2.00e-02 ± 0.00e+00 | 5.23 ± 3.63 | 66.32 ± 39.02 | 0.90 ± 0.02 | 0.06 ± 0.04 |
| Histogram Uniformity | 4.00e-02 ± 1.00e-02 | 8.05 ± 5.57 | 149.23 ± 104.70 | 0.90 ± 0.02 | 0.04 ± 0.04 |
| Histogram Variance | 0.00e+00 ± 0.00e+00 | 10.06 ± 6.82 | 191.55 ± 141.25 | 0.90 ± 0.02 | 0.04 ± 0.04 |
| Histogram TotalFrequency | 4.90e+04 ± 3.61e+04 | 1.69 ± 1.77 | 52.25 ± 24.51 | 1.00 ± 0.00 | 0.26 ± 0.26 |
| Signal Quantile 0.01 | 3.62e+02 ± 1.59e+02 | 58.43 ± 17.06 | 97.70 ± 1.82 | 0.33 ± 0.03 | 0.00 ± 0.00 |
| Signal Quantile 0.1 | 8.35e+02 ± 1.92e+02 | 20.29 ± 15.96 | 91.61 ± 5.47 | 0.58 ± 0.06 | 0.00 ± 0.00 |
| Signal Quantile 0.2 | 1.01e+03 ± 2.34e+02 | 10.61 ± 8.07 | 86.94 ± 7.74 | 0.79 ± 0.04 | 0.01 ± 0.01 |
| Signal Quantile 0.3 | 1.15e+03 ± 2.76e+02 | 8.09 ± 5.69 | 83.43 ± 9.11 | 0.87 ± 0.03 | 0.01 ± 0.01 |
| Signal Quantile 0.4 | 1.30e+03 ± 3.32e+02 | 7.08 ± 5.01 | 80.62 ± 10.09 | 0.90 ± 0.01 | 0.01 ± 0.01 |
| Signal Quantile 0.5 | 1.46e+03 ± 3.96e+02 | 6.05 ± 4.12 | 78.14 ± 10.88 | 0.95 ± 0.01 | 0.02 ± 0.02 |
| Signal Quantile 0.6 | 1.61e+03 ± 4.29e+02 | 4.74 ± 3.17 | 75.55 ± 11.90 | 0.97 ± 0.00 | 0.03 ± 0.02 |
| Signal Quantile 0.7 | 1.76e+03 ± 4.49e+02 | 3.54 ± 2.05 | 72.57 ± 13.14 | 0.99 ± 0.00 | 0.03 ± 0.02 |
| Signal Quantile 0.8 | 1.94e+03 ± 4.37e+02 | 2.89 ± 2.15 | 68.81 ± 14.88 | 0.99 ± 0.00 | 0.03 ± 0.02 |
| Signal Quantile 0.9 | 2.19e+03 ± 4.02e+02 | 2.36 ± 2.56 | 63.95 ± 16.90 | 0.99 ± 0.00 | 0.03 ± 0.02 |
| Signal Quantile 0.99 | 2.71e+03 ± 3.19e+02 | 1.84 ± 3.46 | 53.30 ± 17.55 | 0.96 ± 0.03 | 0.04 ± 0.03 |

**Table 15** Grey Level Co-occurrence Matrix (GLCM) features for the patients of the Soft-Tissues Sarcomas (STS) dataset. In the table, the features computed for 16-bins histogram discretization are presented. The first column shows the mean and standard deviations of the original radiomic features computed on all the patients. The mean and standard deviation of both percentage difference and Intra-class Correlation Coefficient (ICC) are shown for both minimal and maximal entity translations. Values are shown with scientific format only for the original features. The numbers are approximated to the second decimal significant digit.

| **GREY LEVEL CO-OCCURRENCE MATRIX (16-BINS DISCRETIZATION)** | | | | | |
| --- | --- | --- | --- | --- | --- |
| **Feature name** | **Original value** | **Diff %**  **(min. entity)** | **Diff %**  **(max. entity)** | **ICC_10_** | **ICC_100_** |
| Autocorrelation | 3.50e+01 ± 1.68e+01 | 9.00 ± 5.09 | 91.04 ± 11.29 | 0.97 ± 0.00 | 0.01 ± 0.01 |
| Cluster Prominence | 3.38e+04 ± 3.10e+04 | 12.28 ± 9.78 | 95.70 ± 9.07 | 0.98 ± 0.00 | 0.01 ± 0.01 |
| Cluster Shade | 2.08e+03 ± 1.46e+03 | 10.70 ± 6.87 | 93.22 ± 13.95 | 0.97 ± 0.00 | 0.01 ± 0.01 |
| Cluster Tendency | 1.41e+02 ± 6.70e+01 | 8.77 ± 4.96 | 90.07 ± 12.13 | 0.97 ± 0.00 | 0.02 ± 0.01 |
| Contrast | 3.65e+00 ± 1.18e+00 | 8.94 ± 7.93 | 63.27 ± 42.92 | 0.94 ± 0.02 | 0.06 ± 0.10 |
| Correlation | 6.98e+03 ± 5.99e+03 | 8.83 ± 9.30 | 93.99 ± 12.63 | 0.99 ± 0.00 | 0.01 ± 0.02 |
| Difference Entropy | 2.17e+00 ± 1.70e-01 | 2.43 ± 2.01 | 25.34 ± 13.05 | 0.93 ± 0.02 | 0.04 ± 0.05 |
| Dissimilarity | 1.34e+00 ± 2.20e-01 | 4.57 ± 3.50 | 39.96 ± 20.98 | 0.94 ± 0.02 | 0.04 ± 0.05 |
| Energy | 4.00e-02 ± 2.00e-02 | 10.51 ± 6.67 | 391.44 ± 451.13 | 0.94 ± 0.02 | 0.03 ± 0.03 |
| Entropy | 5.54e+00 ± 4.60e-01 | 3.11 ± 2.18 | 35.37 ± 18.36 | 0.91 ± 0.03 | 0.04 ± 0.03 |
| Homogeneity | 5.70e-01 ± 4.00e-02 | 1.65 ± 1.15 | 20.41 ± 14.86 | 0.95 ± 0.01 | 0.02 ± 0.02 |
| Homogeneity2 | 5.20e-01 ± 4.00e-02 | 2.23 ± 1.54 | 26.98 ± 19.70 | 0.95 ± 0.01 | 0.02 ± 0.02 |
| IMOC1 | -1.20e-01 ± 5.00e-02 | 20.66 ± 22.32 | 69.85 ± 60.99 | 0.89 ± 0.01 | 0.10 ± 0.20 |
| IMOC2 | 6.80e-01 ± 1.20e-01 | 7.55 ± 8.07 | 43.31 ± 28.55 | 0.87 ± 0.02 | 0.08 ± 0.11 |
| Inertia | 3.65e+00 ± 1.18e+00 | 8.94 ± 7.93 | 63.27 ± 42.92 | 0.94 ± 0.02 | 0.06 ± 0.10 |
| Inverse Difference moment | 1.00e+00 ± 0.00e+00 | 0.00 ± 0.00 | 0.00 ± 0.00 | 0.94 ± 0.02 | 0.06 ± 0.10 |
| Inverse Difference moment2 | 9.90e-01 ± 0.00e+00 | 0.02 ± 0.02 | 0.21 ± 0.12 | 0.94 ± 0.02 | 0.04 ± 0.05 |
| Inverse Variance | 4.40e-01 ± 3.00e-02 | 1.42 ± 1.27 | 10.16 ± 9.08 | 0.95 ± 0.02 | 0.07 ± 0.10 |
| Max Probability | 1.00e-01 ± 5.00e-02 | 7.56 ± 5.26 | 282.61 ± 348.73 | 0.98 ± 0.01 | 0.03 ± 0.04 |
| Sum Average | 1.10e+01 ± 2.66e+00 | 5.89 ± 3.24 | 78.62 ± 13.68 | 0.96 ± 0.00 | 0.02 ± 0.01 |
| Sum Entropy | 3.76e+00 ± 3.20e-01 | 3.49 ± 2.57 | 32.53 ± 17.34 | 0.89 ± 0.03 | 0.07 ± 0.04 |

**Table 16** Grey Level Co-occurrence Matrix (GLCM) features for the patients of the Soft-Tissues Sarcomas (STS) dataset. In the table, the features computed for 32-bins histogram discretization are presented. The first column shows the mean and standard deviations of the original radiomic features computed on all the patients. The mean and standard deviation of both percentage difference and Intra-class Correlation Coefficient (ICC) are shown for both minimal and maximal entity translations. Values are shown with scientific format only for the original features. The numbers are approximated to the second decimal significant digit.

| **GREY LEVEL CO-OCCURRENCE MATRIX (32-BINS DISCRETIZATION)** | | | | | |
| --- | --- | --- | --- | --- | --- |
| **Feature name** | **Original value** | **Diff %**  **(min. entity)** | **Diff %**  **(max. entity)** | **ICC_10_** | **ICC_100_** |
| Autocorrelation | 1.51e+02 ± 6.97e+01 | 8.74 ± 4.90 | 89.98 ± 11.40 | 0.97 ± 0.00 | 0.02 ± 0.01 |
| Cluster Prominence | 6.23e+05 ± 5.53e+05 | 12.00 ± 9.19 | 95.30 ± 9.53 | 0.98 ± 0.00 | 0.01 ± 0.01 |
| Cluster Shade | 1.87e+04 ± 1.26e+04 | 10.42 ± 6.53 | 92.71 ± 14.13 | 0.97 ± 0.00 | 0.01 ± 0.01 |
| Cluster Tendency | 6.15e+02 ± 2.80e+02 | 8.49 ± 4.76 | 88.97 ± 12.18 | 0.97 ± 0.00 | 0.02 ± 0.01 |
| Contrast | 1.41e+01 ± 4.70e+00 | 9.38 ± 8.38 | 62.90 ± 45.08 | 0.94 ± 0.02 | 0.06 ± 0.11 |
| Correlation | 1.21e+05 ± 9.97e+04 | 9.06 ± 9.45 | 93.04 ± 13.49 | 0.99 ± 0.00 | 0.02 ± 0.02 |
| Difference Entropy | 3.00e+00 ± 1.90e-01 | 1.99 ± 1.60 | 19.56 ± 10.39 | 0.93 ± 0.02 | 0.04 ± 0.05 |
| Dissimilarity | 2.68e+00 ± 4.30e-01 | 4.61 ± 3.56 | 36.02 ± 19.49 | 0.94 ± 0.02 | 0.05 ± 0.06 |
| Energy | 1.00e-02 ± 0.00e+00 | 11.11 ± 6.89 | 381.51 ± 464.57 | 0.94 ± 0.02 | 0.03 ± 0.03 |
| Entropy | 7.46e+00 ± 4.70e-01 | 2.41 ± 1.61 | 26.17 ± 13.75 | 0.91 ± 0.02 | 0.04 ± 0.03 |
| Homogeneity | 4.20e-01 ± 3.00e-02 | 2.05 ± 1.49 | 21.87 ± 17.10 | 0.95 ± 0.01 | 0.01 ± 0.02 |
| Homogeneity2 | 3.40e-01 ± 4.00e-02 | 2.89 ± 2.11 | 32.45 ± 26.87 | 0.95 ± 0.01 | 0.01 ± 0.01 |
| IMOC1 | -1.00e-01 ± 4.00e-02 | 20.98 ± 23.04 | 66.23 ± 55.41 | 0.87 ± 0.01 | 0.09 ± 0.19 |
| IMOC2 | 7.00e-01 ± 1.10e-01 | 6.72 ± 6.98 | 38.44 ± 25.73 | 0.86 ± 0.02 | 0.07 ± 0.11 |
| Inertia | 1.41e+01 ± 4.70e+00 | 9.38 ± 8.38 | 62.90 ± 45.08 | 0.94 ± 0.02 | 0.06 ± 0.11 |
| Inverse Difference moment | 1.00e+00 ± 0.00e+00 | 0.00 ± 0.00 | 0.00 ± 0.00 | 0.94 ± 0.03 | 0.07 ± 0.11 |
| Inverse Difference moment2 | 1.00e+00 ± 0.00e+00 | 0.01 ± 0.01 | 0.10 ± 0.06 | 0.94 ± 0.02 | 0.05 ± 0.06 |
| Inverse Variance | 3.30e-01 ± 3.00e-02 | 2.58 ± 1.74 | 23.00 ± 16.24 | 0.95 ± 0.01 | 0.05 ± 0.04 |
| Max Probability | 3.00e-02 ± 2.00e-02 | 8.42 ± 6.30 | 273.88 ± 398.52 | 0.97 ± 0.01 | 0.04 ± 0.05 |
| Sum Average | 2.30e+01 ± 5.33e+00 | 5.64 ± 3.11 | 75.53 ± 13.35 | 0.95 ± 0.00 | 0.02 ± 0.01 |
| Sum Entropy | 4.74e+00 ± 3.30e-01 | 2.84 ± 2.02 | 24.45 ± 13.05 | 0.89 ± 0.03 | 0.07 ± 0.04 |

**Table 17** Grey Level Co-occurrence Matrix (GLCM) features for the patients of the Soft-Tissues Sarcomas (STS) dataset. In the table, the features computed for 64-bins histogram discretization are presented. The first column shows the mean and standard deviations of the original radiomic features computed on all the patients. The mean and standard deviation of both percentage difference and Intra-class Correlation Coefficient (ICC) are shown for both minimal and maximal entity translations. Values are shown with scientific format only for the original features. The numbers are approximated to the second decimal significant digit.

| **GREY LEVEL CO-OCCURRENCE MATRIX (64-BINS DISCRETIZATION)** | | | | | |
| --- | --- | --- | --- | --- | --- |
| **Feature name** | **Original value** | **Diff %**  **(min. entity)** | **Diff %**  **(max. entity)** | **ICC_10_** | **ICC_100_** |
| Autocorrelation | 6.27e+02 ± 2.84e+02 | 8.61 ± 4.82 | 89.37 ± 11.46 | 0.97 ± 0.00 | 0.02 ± 0.01 |
| Cluster Prominence | 1.06e+07 ± 9.30e+06 | 11.87 ± 8.95 | 95.10 ± 9.78 | 0.98 ± 0.00 | 0.01 ± 0.01 |
| Cluster Shade | 1.58e+05 ± 1.05e+05 | 10.30 ± 6.39 | 92.48 ± 13.96 | 0.97 ± 0.00 | 0.01 ± 0.01 |
| Cluster Tendency | 2.56e+03 ± 1.14e+03 | 8.37 ± 4.68 | 88.38 ± 12.20 | 0.97 ± 0.00 | 0.02 ± 0.01 |
| Contrast | 5.61e+01 ± 1.88e+01 | 9.53 ± 8.53 | 62.84 ± 46.03 | 0.94 ± 0.02 | 0.06 ± 0.11 |
| Correlation | 2.01e+06 ± 1.64e+06 | 9.07 ± 9.44 | 92.67 ± 13.80 | 0.99 ± 0.00 | 0.02 ± 0.02 |
| Difference Entropy | 3.92e+00 ± 2.00e-01 | 1.62 ± 1.28 | 15.66 ± 8.62 | 0.93 ± 0.02 | 0.04 ± 0.05 |
| Dissimilarity | 5.36e+00 ± 8.60e-01 | 4.64 ± 3.58 | 35.04 ± 19.32 | 0.94 ± 0.02 | 0.05 ± 0.07 |
| Energy | 0.00e+00 ± 0.00e+00 | 11.04 ± 6.85 | 379.67 ± 464.42 | 0.94 ± 0.02 | 0.03 ± 0.03 |
| Entropy | 9.39e+00 ± 4.80e-01 | 1.87 ± 1.22 | 20.82 ± 10.89 | 0.92 ± 0.02 | 0.04 ± 0.04 |
| Homogeneity | 2.90e-01 ± 3.00e-02 | 2.40 ± 1.77 | 24.74 ± 20.20 | 0.95 ± 0.01 | 0.01 ± 0.01 |
| Homogeneity2 | 2.00e-01 ± 3.00e-02 | 3.37 ± 2.56 | 37.75 ± 33.88 | 0.95 ± 0.01 | 0.00 ± 0.00 |
| IMOC1 | -9.00e-02 ± 3.00e-02 | 18.40 ± 19.08 | 58.13 ± 47.99 | 0.82 ± 0.03 | 0.08 ± 0.16 |
| IMOC2 | 7.40e-01 ± 9.00e-02 | 5.52 ± 5.32 | 31.13 ± 21.87 | 0.84 ± 0.03 | 0.05 ± 0.11 |
| Inertia | 5.61e+01 ± 1.88e+01 | 9.53 ± 8.53 | 62.84 ± 46.03 | 0.94 ± 0.02 | 0.06 ± 0.11 |
| Inverse Difference moment | 1.00e+00 ± 0.00e+00 | 0.00 ± 0.00 | 0.00 ± 0.00 | 0.86 ± 0.05 | 0.06 ± 0.11 |
| Inverse Difference moment2 | 1.00e+00 ± 0.00e+00 | 0.01 ± 0.00 | 0.05 ± 0.03 | 0.94 ± 0.02 | 0.05 ± 0.07 |
| Inverse Variance | 2.10e-01 ± 3.00e-02 | 3.24 ± 2.34 | 34.33 ± 28.70 | 0.95 ± 0.01 | 0.01 ± 0.01 |
| Max Probability | 1.00e-02 ± 0.00e+00 | 8.63 ± 6.50 | 253.46 ± 334.99 | 0.97 ± 0.01 | 0.03 ± 0.04 |
| Sum Average | 4.70e+01 ± 1.07e+01 | 5.52 ± 3.05 | 73.97 ± 13.16 | 0.95 ± 0.00 | 0.02 ± 0.01 |
| Sum Entropy | 5.74e+00 ± 3.30e-01 | 2.36 ± 1.65 | 19.90 ± 10.64 | 0.89 ± 0.03 | 0.07 ± 0.04 |

**Table 18** Grey Level Run Length Matrix (GLRLM) features for the patients of the Soft-Tissues Sarcomas (STS) dataset. In the table, the features computed for 16-bins histogram discretization are presented. The first column shows the mean and standard deviations of the original radiomic features computed on all the patients. The mean and standard deviation of both percentage difference and Intra-class Correlation Coefficient (ICC) are shown for both minimal and maximal entity translations. Features excluded by our method are highlighted in red. Values are shown with scientific format only for the original features. The numbers are approximated to the second decimal significant digit.

| **GREY LEVEL RUN LENGTH MATRIX (16-BINS DISCRETIZATION)** | | | | | |
| --- | --- | --- | --- | --- | --- |
| **Feature name** | **Original value** | **Diff %**  **(min. entity)** | **Diff %**  **(max. entity)** | **ICC_10_** | **ICC_100_** |
| Short Run Emphasis | 9.90e-01 ± 1.00e-02 | 0.07 ± 0.08 | 0.57 ± 0.50 | 0.99 ± 0.00 | 0.51 ± 0.03 |
| Long Run Emphasis | 1.04e+00 ± 3.00e-02 | 0.30 ± 0.34 | 2.50 ± 2.36 | 0.99 ± 0.01 | 0.44 ± 0.05 |
| Grey Level Non-Uniformity | 4.82e+03 ± 3.45e+03 | 8.11 ± 5.79 | 48.40 ± 28.07 | 0.99 ± 0.00 | 0.45 ± 0.22 |
| Run Length Non-Uniformity | 3.49e+04 ± 2.44e+04 | 1.76 ± 1.81 | 52.23 ± 23.85 | 1.00 ± 0.00 | 0.28 ± 0.25 |
| Run Percentage | 7.50e-01 ± 4.00e-02 | 1.18 ± 0.93 | 6.81 ± 5.60 | 0.96 ± 0.01 | 0.00 ± 0.00 |
| Low Grey Level Run Emphasis | 5.00e-02 ± 2.00e-02 | 57.86 ± 34.28 | 1104.49 ± 498.52 | 0.56 ± 0.04 | 0.01 ± 0.01 |
| high Grey Level Run Emphasis | 4.75e+01 ± 1.62e+01 | 9.14 ± 4.26 | 84.90 ± 11.57 | 0.95 ± 0.01 | 0.02 ± 0.01 |
| Short Run Low Grey Level Emphasis | 5.00e-02 ± 2.00e-02 | 57.99 ± 34.07 | 1100.21 ± 495.56 | 0.56 ± 0.04 | 0.01 ± 0.01 |
| Short Run High Grey Level Emphasis | 4.70e+01 ± 1.58e+01 | 9.18 ± 4.29 | 84.85 ± 11.61 | 0.95 ± 0.01 | 0.02 ± 0.01 |
| Long Run Low Grey Level Emphasis | 5.00e-02 ± 2.00e-02 | 57.24 ± 35.14 | 1121.17 ± 512.56 | 0.58 ± 0.04 | 0.01 ± 0.01 |
| Long Run High Grey Level Emphasis | 4.97e+01 ± 1.89e+01 | 8.99 ± 4.16 | 85.12 ± 11.44 | 0.96 ± 0.01 | 0.02 ± 0.01 |

**Table 19** Grey Level Run Length Matrix (GLRLM) features for the patients of the Soft-Tissues Sarcomas (STS) dataset. In the table, the features computed for 32-bins histogram discretization are presented. The first column shows the mean and standard deviations of the original radiomic features computed on all the patients. The mean and standard deviation of both percentage difference and Intra-class Correlation Coefficient (ICC) are shown for both minimal and maximal entity translations. Features excluded by our method are highlighted in red. Values are shown with scientific format only for the original features. The numbers are approximated to the second decimal significant digit.

| **GREY LEVEL RUN LENGTH MATRIX (32-BINS DISCRETIZATION)** | | | | | |
| --- | --- | --- | --- | --- | --- |
| **Feature name** | **Original value** | **Diff %**  **(min. entity)** | **Diff %**  **(max. entity)** | **ICC_10_** | **ICC_100_** |
| Short Run Emphasis | 9.80e-01 ± 1.00e-02 | 0.10 ± 0.10 | 0.66 ± 0.57 | 0.98 ± 0.01 | 0.35 ± 0.03 |
| Long Run Emphasis | 1.08e+00 ± 4.00e-02 | 0.43 ± 0.43 | 2.87 ± 2.36 | 0.98 ± 0.01 | 0.34 ± 0.04 |
| Grey Level Non-Uniformity | 3.01e+03 ± 2.18e+03 | 8.86 ± 6.10 | 52.35 ± 30.87 | 0.99 ± 0.00 | 0.44 ± 0.21 |
| Run Length Non-Uniformity | 3.97e+04 ± 2.79e+04 | 1.59 ± 1.84 | 51.79 ± 24.29 | 1.00 ± 0.00 | 0.28 ± 0.26 |
| Run Percentage | 8.60e-01 ± 2.00e-02 | 0.63 ± 0.51 | 3.39 ± 2.82 | 0.95 ± 0.01 | 0.00 ± 0.00 |
| Low Grey Level Run Emphasis | 2.00e-02 ± 1.00e-02 | 94.21 ± 60.58 | 2217.57 ± 1131.95 | 0.44 ± 0.05 | 0.01 ± 0.01 |
| high Grey Level Run Emphasis | 1.78e+02 ± 6.65e+01 | 9.09 ± 4.39 | 86.85 ± 11.34 | 0.96 ± 0.00 | 0.02 ± 0.01 |
| Short Run Low Grey Level Emphasis | 2.00e-02 ± 1.00e-02 | 94.46 ± 59.96 | 2203.33 ± 1119.37 | 0.44 ± 0.05 | 0.01 ± 0.01 |
| Short Run High Grey Level Emphasis | 1.75e+02 ± 6.42e+01 | 9.13 ± 4.41 | 86.76 ± 11.44 | 0.96 ± 0.00 | 0.02 ± 0.01 |
| Long Run Low Grey Level Emphasis | 2.00e-02 ± 1.00e-02 | 93.21 ± 63.79 | 2275.88 ± 1190.06 | 0.44 ± 0.05 | 0.01 ± 0.01 |
| Long Run High Grey Level Emphasis | 1.92e+02 ± 7.78e+01 | 8.92 ± 4.27 | 87.20 ± 10.97 | 0.96 ± 0.00 | 0.02 ± 0.01 |

**Table 20** Grey Level Run Length Matrix (GLRLM) features for the patients of the Soft-Tissues Sarcomas (STS) dataset. In the table, the features computed for 64-bins histogram discretization are presented. The first column shows the mean and standard deviations of the original radiomic features computed on all the patients. The mean and standard deviation of both percentage difference and Intra-class Correlation Coefficient (ICC) are shown for both minimal and maximal entity translations. Features excluded by our method are highlighted in red. Values are shown with scientific format only for the original features. The numbers are approximated to the second decimal significant digit.

| **GREY LEVEL RUN LENGTH MATRIX (64-BINS DISCRETIZATION)** | | | | | |
| --- | --- | --- | --- | --- | --- |
| **Feature name** | **Original value** | **Diff %**  **(min. entity)** | **Diff %**  **(max. entity)** | **ICC_10_** | **ICC_100_** |
| Short Run Emphasis | 9.50e-01 ± 2.00e-02 | 0.19 ± 0.17 | 1.06 ± 0.93 | 0.99 ± 0.00 | 0.75 ± 0.02 |
| Long Run Emphasis | 1.24e+00 ± 9.00e-02 | 0.71 ± 0.67 | 4.31 ± 3.60 | 0.99 ± 0.00 | 0.67 ± 0.02 |
| Grey Level Non-Uniformity | 1.69e+03 ± 1.24e+03 | 9.25 ± 6.19 | 53.69 ± 32.94 | 0.98 ± 0.00 | 0.44 ± 0.21 |
| Run Length Non-Uniformity | 3.91e+04 ± 2.74e+04 | 1.62 ± 1.93 | 51.76 ± 24.46 | 1.00 ± 0.00 | 0.27 ± 0.25 |
| Run Percentage | 9.30e-01 ± 1.00e-02 | 0.33 ± 0.26 | 1.68 ± 1.30 | 0.95 ± 0.01 | 0.00 ± 0.00 |
| Low Grey Level Run Emphasis | 1.00e-02 ± 0.00e+00 | 142.38 ± 95.74 | 3854.75 ± 2197.69 | 0.37 ± 0.05 | 0.00 ± 0.01 |
| high Grey Level Run Emphasis | 6.86e+02 ± 2.69e+02 | 9.04 ± 4.44 | 87.64 ± 11.27 | 0.96 ± 0.00 | 0.02 ± 0.01 |
| Short Run Low Grey Level Emphasis | 1.00e-02 ± 0.00e+00 | 141.74 ± 93.01 | 3784.17 ± 2151.95 | 0.38 ± 0.04 | 0.01 ± 0.01 |
| Short Run High Grey Level Emphasis | 6.51e+02 ± 2.49e+02 | 9.13 ± 4.49 | 87.46 ± 11.50 | 0.96 ± 0.01 | 0.02 ± 0.01 |
| Long Run Low Grey Level Emphasis | 1.00e-02 ± 0.00e+00 | 143.94 ± 107.17 | 4114.62 ± 2392.79 | 0.35 ± 0.05 | 0.01 ± 0.00 |
| Long Run High Grey Level Emphasis | 8.48e+02 ± 3.77e+02 | 8.73 ± 4.27 | 88.30 ± 10.52 | 0.97 ± 0.00 | 0.02 ± 0.01 |

1. **TABLES OF FEATURES (ROTATIONS)**

In this section the tables with the percentage variation of radiomic features and ICC after the application of rotation along z (vertical axes) is presented. The stability analysis was performed only considering the minimum entity rotation corresponding to ± 18°. No feature relevance analysis like the one performed for the translation could be made, because even for large rotations (between 90° and 180° degree) the majority of the original ROI will overlap with the transformed one, and so the resulting ICC is expected to be high. If the ICC_MEAN_ of more rotation is below 0.78 (threshold taken from the main paper), we consider the feature unstable.

- 1. **Oropharyngeal cancers dataset**

**Table 21** First-Order Statistics (FOS) features for the patients of the OroPharyngeal Cancers (OPC) dataset. In the table, the bin-independent features are presented. The first column shows the mean and standard deviations of the original radiomic features computed on all the patients. The mean and standard deviation of both percentage difference and Intra-class Correlation Coefficient (ICC) are shown for small entity rotations. Values are shown with scientific format only for the original features. The numbers are approximated to the second decimal significant digit. Features for which mean ICC_SMALL_ is less than 0.78 (unstable features) are highlighted in red.

| **FIRST-ORDER STATISTICS (BIN-INDEPENDENT)** | | | |
| --- | --- | --- | --- |
| **Feature name** | **Original value** | **Diff %** | **ICC_SMALL_** |
| Signal Energy | 2.37e+09 ± 1.92e+09 | 9.38 ± 5.23 | 0.99 ± 0.00 |
| Signal Kurtosis | 3.24e+00 ± 7.17e-01 | 5.74 ± 6.63 | 0.93 ± 0.03 |
| Signal Mad | 3.01e+02 ± 5.77e+01 | 2.88 ± 3.32 | 0.98 ± 0.00 |
| Signal Max | 2.41e+03 ± 4.14e+02 | 1.48 ± 3.27 | 0.98 ± 0.01 |
| Signal Mean | 1.05e+03 ± 1.59e+02 | 1.38 ± 1.08 | 0.99 ± 0.00 |
| Signal Median | 9.83e+02 ± 1.49e+02 | 1.56 ± 1.20 | 0.99 ± 0.00 |
| Signal Min | 1.01e+02 ± 1.24e+02 | 35.87 ± 50.33 | 0.53 ± 0.16 |
| Signal Range | 2.31e+03 ± 4.54e+02 | 3.86 ± 7.40 | 0.93 ± 0.03 |
| Signal RMS | 1.12e+03 ± 1.53e+02 | 1.32 ± 1.11 | 0.99 ± 0.00 |
| Signal Skewness | 5.13e-01 ± 3.61e-01 | 17.06 ± 23.97 | 0.98 ± 0.01 |
| Signal STD | 3.72e+02 ± 5.90e+01 | 2.56 ± 3.07 | 0.98 ± 0.00 |
| Signal Variance | 1.42e+05 ± 4.39e+04 | 5.05 ± 5.90 | 0.98 ± 0.00 |

**Table 22** First-Order Statistics (FOS) features for the patients of the OroPharyngeal Cancers (OPC) dataset. In the table, the bin-dependent features calculated with a 16-bins discretization are presented. The first column shows the mean and standard deviations of the original radiomic features computed on all the patients. The mean and standard deviation of both percentage difference and Intra-class Correlation Coefficient (ICC) are shown for small entity rotations. Values are shown with scientific format only for the original features. The numbers are approximated to the second decimal significant digit.

| **FIRST-ORDER STATISTICS (16-BINS DISCRETIZATION)** | | | |
| --- | --- | --- | --- |
| **Feature name** | **Original value** | **Diff %** | **ICC_SMALL_** |
| Histogram Entropy | 2.51e+00 ± 1.91e-01 | 1.60 ± 1.84 | 0.96 ± 0.01 |
| Histogram Kurtosis | 9.46e+00 ± 2.26e+00 | 5.29 ± 4.05 | 0.97 ± 0.01 |
| Histogram Mad | 4.86e-02 ± 1.24e-03 | 0.77 ± 0.85 | 0.90 ± 0.07 |
| Histogram Max | 3.17e-01 ± 5.13e-02 | 3.75 ± 3.15 | 0.96 ± 0.01 |
| Histogram Mean | 3.13e-02 ± 0.00e+00 | 0.00 ± 0.00 | 1.00 ± 0.00 |
| Histogram Median | 0.00e+00 ± 0.00e+00 | 0.00 ± 0.00 | 1.00 ± 0.00 |
| Histogram Min | 0.00e+00 ± 0.00e+00 | 0.00 ± 0.00 | 1.00 ± 0.00 |
| Histogram Range | 3.17e-01 ± 5.13e-02 | 3.75 ± 3.15 | 0.96 ± 0.01 |
| Histogram RMS | 8.12e-02 ± 6.28e-03 | 1.44 ± 1.64 | 0.96 ± 0.02 |
| Histogram Skewness | 2.67e+00 ± 3.72e-01 | 2.94 ± 2.38 | 0.97 ± 0.01 |
| Histogram STD | 7.61e-02 ± 6.90e-03 | 1.69 ± 1.90 | 0.96 ± 0.02 |
| Histogram Uniformity | 2.12e-01 ± 3.35e-02 | 2.90 ± 3.41 | 0.96 ± 0.02 |
| Histogram Variance | 5.83e-03 ± 1.08e-03 | 3.41 ± 3.97 | 0.96 ± 0.02 |
| Histogram TotalFrequency | 1.81e+03 ± 1.34e+03 | 8.26 ± 3.74 | 0.99 ± 0.00 |
| Signal Quantile 0.01 | 3.25e+02 ± 1.92e+02 | 11.70 ± 14.31 | 0.97 ± 0.01 |
| Signal Quantile 0.1 | 6.10e+02 ± 1.76e+02 | 2.51 ± 3.16 | 0.99 ± 0.00 |
| Signal Quantile 0.2 | 7.24e+02 ± 1.64e+02 | 1.71 ± 1.69 | 1.00 ± 0.00 |
| Signal Quantile 0.3 | 8.16e+02 ± 1.55e+02 | 1.43 ± 1.04 | 1.00 ± 0.00 |
| Signal Quantile 0.4 | 9.01e+02 ± 1.49e+02 | 1.31 ± 0.96 | 1.00 ± 0.00 |
| Signal Quantile 0.5 | 9.93e+02 ± 1.48e+02 | 1.42 ± 0.95 | 0.99 ± 0.00 |
| Signal Quantile 0.6 | 1.10e+03 ± 1.56e+02 | 1.71 ± 1.49 | 0.99 ± 0.00 |
| Signal Quantile 0.7 | 1.23e+03 ± 1.75e+02 | 1.71 ± 1.49 | 0.99 ± 0.00 |
| Signal Quantile 0.8 | 1.38e+03 ± 1.89e+02 | 1.66 ± 1.71 | 0.99 ± 0.00 |
| Signal Quantile 0.9 | 1.58e+03 ± 2.04e+02 | 1.49 ± 1.86 | 0.99 ± 0.01 |
| Signal Quantile 0.99 | 2.04e+03 ± 2.39e+02 | 1.51 ± 1.45 | 0.99 ± 0.00 |

**Table 23** First-Order Statistics (FOS) features for the patients of the OroPharyngeal Cancers (OPC) dataset. In the table, the bin-dependent features calculated with a 32-bins discretization are presented. The first column shows the mean and standard deviations of the original radiomic features computed on all the patients. The mean and standard deviation of both percentage difference and Intra-class Correlation Coefficient (ICC) are shown for small entity rotations. Values are shown with scientific format only for the original features. The numbers are approximated to the second decimal significant digit.

| **FIRST-ORDER STATISTICS (32-BINS DISCRETIZATION)** | | | |
| --- | --- | --- | --- |
| **Feature name** | **Original value** | **Diff %** | **ICC_SMALL_** |
| Histogram Entropy | 3.47e+00 ± 2.05e-01 | 1.11 ± 1.28 | 0.96 ± 0.01 |
| Histogram Kurtosis | 1.08e+01 ± 2.95e+00 | 5.40 ± 3.65 | 0.97 ± 0.01 |
| Histogram Mad | 2.44e-02 ± 6.61e-04 | 0.77 ± 0.70 | 0.93 ± 0.02 |
| Histogram Max | 1.84e-01 ± 3.69e-02 | 3.69 ± 2.99 | 0.98 ± 0.00 |
| Histogram Mean | 1.56e-02 ± 0.00e+00 | 0.00 ± 0.00 | 1.00 ± 0.00 |
| Histogram Median | 0.00e+00 ± 0.00e+00 | 0.00 ± 0.00 | 1.00 ± 0.00 |
| Histogram Min | 0.00e+00 ± 0.00e+00 | 0.00 ± 0.00 | 1.00 ± 0.00 |
| Histogram Range | 1.84e-01 ± 3.69e-02 | 3.69 ± 2.99 | 0.98 ± 0.00 |
| Histogram RMS | 4.14e-02 ± 3.43e-03 | 1.46 ± 1.31 | 0.97 ± 0.01 |
| Histogram Skewness | 2.83e+00 ± 4.32e-01 | 2.86 ± 1.89 | 0.97 ± 0.01 |
| Histogram STD | 3.86e-02 ± 3.73e-03 | 1.69 ± 1.50 | 0.97 ± 0.01 |
| Histogram Uniformity | 1.11e-01 ± 1.86e-02 | 2.94 ± 2.68 | 0.97 ± 0.01 |
| Histogram Variance | 1.51e-03 ± 2.95e-04 | 3.42 ± 3.08 | 0.97 ± 0.01 |
| Histogram TotalFrequency | 1.81e+03 ± 1.34e+03 | 8.26 ± 3.74 | 0.99 ± 0.00 |
| Signal Quantile 0.01 | 3.49e+02 ± 2.12e+02 | 12.43 ± 15.56 | 0.97 ± 0.01 |
| Signal Quantile 0.1 | 6.27e+02 ± 1.80e+02 | 2.42 ± 3.02 | 0.99 ± 0.00 |
| Signal Quantile 0.2 | 7.32e+02 ± 1.62e+02 | 1.62 ± 1.60 | 1.00 ± 0.00 |
| Signal Quantile 0.3 | 8.14e+02 ± 1.54e+02 | 1.34 ± 1.05 | 1.00 ± 0.00 |
| Signal Quantile 0.4 | 8.94e+02 ± 1.49e+02 | 1.33 ± 0.96 | 1.00 ± 0.00 |
| Signal Quantile 0.5 | 9.85e+02 ± 1.48e+02 | 1.62 ± 1.31 | 0.99 ± 0.00 |
| Signal Quantile 0.6 | 1.10e+03 ± 1.56e+02 | 1.86 ± 1.57 | 0.98 ± 0.00 |
| Signal Quantile 0.7 | 1.22e+03 ± 1.74e+02 | 1.72 ± 1.46 | 0.99 ± 0.00 |
| Signal Quantile 0.8 | 1.37e+03 ± 1.94e+02 | 1.72 ± 1.61 | 0.99 ± 0.00 |
| Signal Quantile 0.9 | 1.57e+03 ± 2.02e+02 | 1.51 ± 2.01 | 0.98 ± 0.01 |
| Signal Quantile 0.99 | 2.02e+03 ± 2.29e+02 | 1.49 ± 1.72 | 0.98 ± 0.01 |

**Table 24** First-Order Statistics (FOS) features for the patients of the OroPharyngeal Cancers (OPC) dataset. In the table, the bin-dependent features calculated with a 64-bins discretization are presented. The first column shows the mean and standard deviations of the original radiomic features computed on all the patients. The mean and standard deviation of both percentage difference and Intra-class Correlation Coefficient (ICC) are shown for small entity rotations. Values are shown with scientific format only for the original features. The numbers are approximated to the second decimal significant digit.

| **FIRST-ORDER STATISTICS (64-BINS DISCRETIZATION)** | | | |
| --- | --- | --- | --- |
| **Feature name** | **Original value** | **Diff %** | **ICC_SMALL_** |
| Histogram Entropy | 4.44e+00 ± 2.12e-01 | 0.97 ± 1.10 | 0.96 ± 0.01 |
| Histogram Kurtosis | 1.14e+01 ± 3.10e+00 | 6.23 ± 5.29 | 0.96 ± 0.01 |
| Histogram Mad | 1.23e-02 ± 3.37e-04 | 0.70 ± 0.59 | 0.95 ± 0.00 |
| Histogram Max | 9.88e-02 ± 1.76e-02 | 5.20 ± 4.46 | 0.93 ± 0.00 |
| Histogram Mean | 7.81e-03 ± 0.00e+00 | 0.00 ± 0.00 | 1.00 ± 0.00 |
| Histogram Median | 0.00e+00 ± 0.00e+00 | 0.00 ± 0.00 | 1.00 ± 0.00 |
| Histogram Min | 0.00e+00 ± 0.00e+00 | 0.00 ± 0.00 | 1.00 ± 0.00 |
| Histogram Range | 9.88e-02 ± 1.76e-02 | 5.20 ± 4.46 | 0.93 ± 0.00 |
| Histogram RMS | 2.10e-02 ± 1.77e-03 | 1.64 ± 1.68 | 0.96 ± 0.01 |
| Histogram Skewness | 2.91e+00 ± 4.47e-01 | 3.35 ± 2.44 | 0.97 ± 0.00 |
| Histogram STD | 1.95e-02 ± 1.91e-03 | 1.89 ± 1.91 | 0.96 ± 0.01 |
| Histogram Uniformity | 5.67e-02 ± 9.73e-03 | 3.32 ± 3.48 | 0.96 ± 0.01 |
| Histogram Variance | 3.85e-04 ± 7.66e-05 | 3.83 ± 3.97 | 0.96 ± 0.01 |
| Histogram TotalFrequency | 1.81e+03 ± 1.34e+03 | 8.26 ± 3.74 | 0.99 ± 0.00 |
| Signal Quantile 0.01 | 3.58e+02 ± 2.16e+02 | 13.42 ± 15.68 | 0.97 ± 0.02 |
| Signal Quantile 0.1 | 6.33e+02 ± 1.78e+02 | 1.98 ± 2.21 | 1.00 ± 0.00 |
| Signal Quantile 0.2 | 7.37e+02 ± 1.62e+02 | 1.56 ± 1.62 | 1.00 ± 0.00 |
| Signal Quantile 0.3 | 8.16e+02 ± 1.52e+02 | 1.39 ± 1.24 | 1.00 ± 0.00 |
| Signal Quantile 0.4 | 8.92e+02 ± 1.50e+02 | 1.33 ± 1.08 | 0.99 ± 0.00 |
| Signal Quantile 0.5 | 9.83e+02 ± 1.49e+02 | 1.55 ± 1.17 | 0.99 ± 0.00 |
| Signal Quantile 0.6 | 1.10e+03 ± 1.56e+02 | 1.84 ± 1.63 | 0.98 ± 0.00 |
| Signal Quantile 0.7 | 1.22e+03 ± 1.75e+02 | 1.81 ± 1.43 | 0.99 ± 0.00 |
| Signal Quantile 0.8 | 1.37e+03 ± 1.95e+02 | 1.68 ± 1.55 | 0.99 ± 0.00 |
| Signal Quantile 0.9 | 1.57e+03 ± 2.04e+02 | 1.50 ± 1.96 | 0.98 ± 0.01 |
| Signal Quantile 0.99 | 2.02e+03 ± 2.26e+02 | 1.78 ± 2.42 | 0.97 ± 0.01 |

**Table 25** Grey Level Co-occurrence Matrix (GLCM) features for the patients of the OroPharyngeal Cancers (OPC) dataset. In the table, the features computed for 16-bins histogram discretization are presented. The first column shows the mean and standard deviations of the original radiomic features computed on all the patients. The mean and standard deviation of both percentage difference and Intra-class Correlation Coefficient (ICC) are shown for small entity rotations. Values are shown with scientific format only for the original features. The numbers are approximated to the second decimal significant digit. Features for which mean ICC_SMALL_ is less than 0.78 (unstable features) are highlighted in red.

| **GREY LEVEL CO-OCCURRENCE MATRIX (16-BINS DISCRETIZATION)** | | | |
| --- | --- | --- | --- |
| **Feature name** | **Original value** | **Diff %** | **ICC_SMALL_** |
| Autocorrelation | 1.40e+01 ± 4.53e+00 | 3.44 ± 2.74 | 0.99 ± 0.00 |
| Cluster Prominence | 5.14e+03 ± 2.78e+03 | 6.95 ± 5.90 | 0.99 ± 0.00 |
| Cluster Shade | 5.14e+02 ± 2.20e+02 | 5.23 ± 4.32 | 0.99 ± 0.00 |
| Cluster Tendency | 5.68e+01 ± 1.75e+01 | 3.43 ± 2.82 | 0.99 ± 0.00 |
| Contrast | 2.63e+00 ± 8.39e-01 | 8.24 ± 7.78 | 0.95 ± 0.00 |
| Correlation | 1.27e+03 ± 5.16e+02 | 8.11 ± 6.38 | 0.97 ± 0.01 |
| Difference Entropy | 1.97e+00 ± 1.87e-01 | 2.43 ± 2.48 | 0.94 ± 0.00 |
| Dissimilarity | 1.16e+00 ± 2.15e-01 | 4.03 ± 4.39 | 0.96 ± 0.01 |
| Energy | 7.10e-02 ± 2.28e-02 | 6.38 ± 5.01 | 0.96 ± 0.01 |
| Entropy | 4.53e+00 ± 4.62e-01 | 2.11 ± 2.04 | 0.97 ± 0.01 |
| Homogeneity | 5.94e-01 ± 4.75e-02 | 1.62 ± 1.73 | 0.95 ± 0.01 |
| Homogeneity2 | 5.54e-01 ± 5.82e-02 | 2.14 ± 2.26 | 0.95 ± 0.01 |
| IMOC1 | -1.39e-01 ± 1.08e-01 | 19.17 ± 16.97 | 0.64 ± 0.43 |
| IMOC2 | 6.14e-01 ± 1.32e-01 | 4.24 ± 3.36 | 0.97 ± 0.01 |
| Inertia | 1.00e+00 ± 1.28e-05 | 0.00 ± 0.00 | 0.95 ± 0.00 |
| Inverse Difference moment | 9.96e-01 ± 8.27e-04 | 0.02 ± 0.02 | 0.96 ± 0.01 |
| Inverse Difference moment2 | 4.55e-01 ± 3.79e-02 | 2.19 ± 1.92 | 0.94 ± 0.00 |
| Inverse Variance | 1.66e-01 ± 4.91e-02 | 6.84 ± 4.42 | 0.97 ± 0.00 |
| Max Probability | 7.16e+00 ± 1.21e+00 | 1.65 ± 1.36 | 0.99 ± 0.00 |
| Sum Average | 3.11e+00 ± 2.60e-01 | 1.52 ± 1.49 | 0.97 ± 0.01 |
| Sum Entropy | 2.63e+00 ± 8.39e-01 | 8.24 ± 7.78 | 0.95 ± 0.00 |

**Table 26** Grey Level Co-occurrence Matrix (GLCM) features for the patients of the OroPharyngeal Cancers (OPC) dataset. In the table, the features computed for 32-bins histogram discretization are presented. The first column shows the mean and standard deviations of the original radiomic features computed on all the patients. The mean and standard deviation of both percentage difference and Intra-class Correlation Coefficient (ICC) are shown for small entity rotations. Values are shown with scientific format only for the original features. The numbers are approximated to the second decimal significant digit. Features for which mean ICC_SMALL_ is less than 0.7 (unstable features) are highlighted in red.

| **GREY LEVEL CO-OCCURRENCE MATRIX (32-BINS DISCRETIZATION)** | | | |
| --- | --- | --- | --- |
| **Feature name** | **Original value** | **Diff %** | **ICC_SMALL_** |
| Autocorrelation | 6.32e+01 ± 1.93e+01 | 3.29 ± 2.71 | 0.99 ± 0.00 |
| Cluster Prominence | 1.03e+05 ± 5.31e+04 | 6.71 ± 5.75 | 0.99 ± 0.00 |
| Cluster Shade | 4.95e+03 ± 2.02e+03 | 4.97 ± 4.24 | 0.99 ± 0.00 |
| Cluster Tendency | 2.61e+02 ± 7.56e+01 | 3.21 ± 2.76 | 0.99 ± 0.00 |
| Contrast | 1.01e+01 ± 3.44e+00 | 8.06 ± 8.18 | 0.96 ± 0.00 |
| Correlation | 2.23e+04 ± 9.16e+03 | 7.95 ± 6.16 | 0.97 ± 0.00 |
| Difference Entropy | 2.77e+00 ± 2.26e-01 | 1.81 ± 2.39 | 0.94 ± 0.03 |
| Dissimilarity | 2.33e+00 ± 4.43e-01 | 3.97 ± 4.47 | 0.96 ± 0.01 |
| Energy | 2.32e-02 ± 1.04e-02 | 6.43 ± 5.76 | 0.97 ± 0.01 |
| Entropy | 6.18e+00 ± 6.42e-01 | 1.65 ± 1.61 | 0.98 ± 0.00 |
| Homogeneity | 4.43e-01 ± 4.81e-02 | 2.13 ± 2.11 | 0.96 ± 0.02 |
| Homogeneity2 | 3.71e-01 ± 5.89e-02 | 3.16 ± 3.14 | 0.96 ± 0.02 |
| IMOC1 | -1.58e-01 ± 1.27e-01 | 17.74 ± 25.04 | 0.57 ± 0.09 |
| IMOC2 | 7.38e-01 ± 1.31e-01 | 2.30 ± 2.17 | 0.99 ± 0.00 |
| Inertia | 1.00e+00 ± 3.35e-06 | 0.00 ± 0.00 | 0.95 ± 0.00 |
| Inverse Difference moment | 9.98e-01 ± 4.29e-04 | 0.01 ± 0.01 | 0.96 ± 0.01 |
| Inverse Difference moment2 | 3.59e-01 ± 4.71e-02 | 2.82 ± 3.48 | 0.92 ± 0.07 |
| Inverse Variance | 6.46e-02 ± 2.83e-02 | 8.70 ± 9.08 | 0.97 ± 0.01 |
| Max Probability | 1.53e+01 ± 2.41e+00 | 1.58 ± 1.29 | 0.99 ± 0.00 |
| Sum Average | 4.06e+00 ± 2.86e-01 | 1.29 ± 1.37 | 0.97 ± 0.01 |
| Sum Entropy | 1.01e+01 ± 3.44e+00 | 8.06 ± 8.18 | 0.96 ± 0.00 |

**Table 27** Grey Level Co-occurrence Matrix (GLCM) features for the patients of the OroPharyngeal Cancers (OPC) dataset. In the table, the features computed for 64-bins histogram discretization are presented. The first column shows the mean and standard deviations of the original radiomic features computed on all the patients. The mean and standard deviation of both percentage difference and Intra-class Correlation Coefficient (ICC) are shown for small entity rotations. Values are shown with scientific format only for the original features. The numbers are approximated to the second decimal significant digit. Features for which mean ICC_SMALL_ is less than 0.7 (unstable features) are highlighted in red.

| **GREY LEVEL CO-OCCURRENCE MATRIX (64-BINS DISCRETIZATION)** | | | |
| --- | --- | --- | --- |
| **Feature name** | **Original value** | **Diff %** | **ICC_SMALL_** |
| Autocorrelation | 2.68e+02 ± 7.98e+01 | 3.19 ± 2.63 | 0.99 ± 0.00 |
| Cluster Prominence | 1.82e+06 ± 9.20e+05 | 6.52 ± 5.70 | 0.99 ± 0.00 |
| Cluster Shade | 4.31e+04 ± 1.71e+04 | 4.80 ± 4.16 | 0.99 ± 0.00 |
| Cluster Tendency | 1.11e+03 ± 3.13e+02 | 3.11 ± 2.66 | 0.99 ± 0.00 |
| Contrast | 4.01e+01 ± 1.39e+01 | 8.19 ± 8.87 | 0.96 ± 0.00 |
| Correlation | 3.70e+05 ± 1.54e+05 | 8.27 ± 7.11 | 0.98 ± 0.00 |
| Difference Entropy | 3.65e+00 ± 2.54e-01 | 1.43 ± 1.38 | 0.97 ± 0.01 |
| Dissimilarity | 4.66e+00 ± 9.05e-01 | 3.98 ± 4.68 | 0.96 ± 0.01 |
| Energy | 8.55e-03 ± 6.12e-03 | 8.16 ± 8.64 | 0.96 ± 0.05 |
| Entropy | 7.62e+00 ± 9.68e-01 | 1.57 ± 1.80 | 0.99 ± 0.01 |
| Homogeneity | 3.10e-01 ± 4.29e-02 | 2.45 ± 2.31 | 0.97 ± 0.00 |
| Homogeneity2 | 2.22e-01 ± 4.75e-02 | 3.94 ± 3.86 | 0.97 ± 0.01 |
| IMOC1 | -1.64e-01 ± 1.10e-01 | 36.76 ± 124.64 | 0.37 ± 0.53 |
| IMOC2 | 8.66e-01 ± 9.76e-02 | 1.31 ± 1.33 | 0.99 ± 0.01 |
| Inertia | 1.00e+00 ± 8.26e-07 | 0.00 ± 0.00 | 0.89 ± 0.04 |
| Inverse Difference moment | 9.99e-01 ± 2.20e-04 | 0.00 ± 0.00 | 0.96 ± 0.01 |
| Inverse Difference moment2 | 2.30e-01 ± 4.85e-02 | 5.03 ± 6.12 | 0.91 ± 0.08 |
| Inverse Variance | 2.54e-02 ± 1.57e-02 | 10.76 ± 11.10 | 0.94 ± 0.07 |
| Max Probability | 3.16e+01 ± 4.83e+00 | 1.53 ± 1.22 | 0.99 ± 0.00 |
| Sum Average | 4.99e+00 ± 3.44e-01 | 1.12 ± 1.14 | 0.98 ± 0.00 |
| Sum Entropy | 4.01e+01 ± 1.39e+01 | 8.19 ± 8.87 | 0.96 ± 0.00 |

**Table 28** Grey Level Run Length Matrix (GLRLM) features for the patients of the OroPharyngeal Cancers (OPC) dataset. In the table, the features computed for 16-bins histogram discretization are presented. The first column shows the mean and standard deviations of the original radiomic features computed on all the patients. The mean and standard deviation of both percentage difference and Intra-class Correlation Coefficient (ICC) are shown for small rotations. Values are shown with scientific format only for the original features. The numbers are approximated to the second decimal significant digit.

| **GREY LEVEL RUN LENGTH MATRIX (16-BINS DISCRETIZATION)** | | | |
| --- | --- | --- | --- |
| **Feature name** | **Original value** | **Diff %** | **ICC_SMALL_** |
| Short Run Emphasis | 9.78e-01 ± 1.43e-02 | 0.20 ± 0.21 | 0.98 ± 0.01 |
| Long Run Emphasis | 1.10e+00 ± 7.03e-02 | 0.81 ± 0.86 | 0.98 ± 0.01 |
| Grey Level Non-Uniformity | 2.55e+02 ± 1.86e+02 | 7.21 ± 3.44 | 0.99 ± 0.00 |
| Run Length Non-Uniformity | 1.31e+03 ± 9.53e+02 | 7.96 ± 3.42 | 0.99 ± 0.00 |
| Run Percentage | 7.91e-01 ± 5.71e-02 | 1.11 ± 1.01 | 0.98 ± 0.01 |
| Low Grey Level Run Emphasis | 7.91e-02 ± 5.02e-02 | 5.19 ± 4.18 | 0.99 ± 0.00 |
| high Grey Level Run Emphasis | 2.62e+01 ± 6.58e+00 | 2.34 ± 2.13 | 0.99 ± 0.00 |
| Short Run Low Grey Level Emphasis | 7.77e-02 ± 5.06e-02 | 5.20 ± 4.16 | 1.00 ± 0.00 |
| Short Run High Grey Level Emphasis | 2.58e+01 ± 6.37e+00 | 2.41 ± 2.08 | 0.99 ± 0.00 |
| Long Run Low Grey Level Emphasis | 8.52e-02 ± 4.86e-02 | 5.20 ± 4.78 | 0.99 ± 0.00 |
| Long Run High Grey Level Emphasis | 2.82e+01 ± 7.77e+00 | 2.30 ± 2.40 | 0.99 ± 0.00 |

**Table 29** Grey Level Run Length Matrix (GLRLM) features for the patients of the OroPharyngeal Cancers (OPC) dataset. In the table, the features computed for 32-bins histogram discretization are presented. The first column shows the mean and standard deviations of the original radiomic features computed on all the patients. The mean and standard deviation of both percentage difference and Intra-class Correlation Coefficient (ICC) are shown for small rotations. Values are shown with scientific format only for the original features. The numbers are approximated to the second decimal significant digit.

| **GREY LEVEL RUN LENGTH MATRIX (32-BINS DISCRETIZATION)** | | | |
| --- | --- | --- | --- |
| **Feature name** | **Original value** | **Diff %** | **ICC_SMALL_** |
| Short Run Emphasis | 9.79e-01 ± 1.10e-02 | 0.13 ± 0.15 | 0.98 ± 0.01 |
| Long Run Emphasis | 1.11e+00 ± 6.23e-02 | 0.78 ± 1.09 | 0.97 ± 0.02 |
| Grey Level Non-Uniformity | 1.59e+02 ± 1.18e+02 | 6.83 ± 3.28 | 0.99 ± 0.00 |
| Run Length Non-Uniformity | 1.49e+03 ± 1.08e+03 | 8.24 ± 3.59 | 0.99 ± 0.00 |
| Run Percentage | 8.88e-01 ± 3.43e-02 | 0.61 ± 0.53 | 0.98 ± 0.01 |
| Low Grey Level Run Emphasis | 2.89e-02 ± 2.63e-02 | 8.48 ± 6.86 | 0.99 ± 0.00 |
| high Grey Level Run Emphasis | 9.26e+01 ± 2.36e+01 | 2.53 ± 2.33 | 0.99 ± 0.00 |
| Short Run Low Grey Level Emphasis | 2.84e-02 ± 2.61e-02 | 8.55 ± 6.93 | 0.99 ± 0.00 |
| Short Run High Grey Level Emphasis | 9.12e+01 ± 2.29e+01 | 2.50 ± 2.38 | 0.99 ± 0.00 |
| Long Run Low Grey Level Emphasis | 3.16e-02 ± 2.72e-02 | 8.08 ± 7.04 | 0.99 ± 0.00 |
| Long Run High Grey Level Emphasis | 9.98e+01 ± 2.75e+01 | 2.73 ± 2.45 | 0.99 ± 0.00 |

**Table 30** Grey Level Run Length Matrix (GLRLM) features for the patients of the OroPharyngeal Cancers (OPC) dataset. In the table, the features computed for 64-bins histogram discretization are presented. The first column shows the mean and standard deviations of the original radiomic features computed on all the patients. The mean and standard deviation of both percentage difference and Intra-class Correlation Coefficient (ICC) are shown for small rotations. Values are shown with scientific format only for the original features. The numbers are approximated to the second decimal significant digit.

| **GREY LEVEL RUN LENGTH MATRIX (64-BINS DISCRETIZATION)** | | | |
| --- | --- | --- | --- |
| **Feature name** | **Original value** | **Diff %** | **ICC_SMALL_** |
| Short Run Emphasis | 9.41e-01 ± 1.25e-02 | 0.40 ± 0.40 | 0.92 ± 0.01 |
| Long Run Emphasis | 1.30e+00 ± 8.36e-02 | 1.48 ± 1.23 | 0.96 ± 0.00 |
| Grey Level Non-Uniformity | 8.94e+01 ± 6.71e+01 | 6.49 ± 3.48 | 1.00 ± 0.00 |
| Run Length Non-Uniformity | 1.43e+03 ± 1.05e+03 | 8.16 ± 4.08 | 0.99 ± 0.00 |
| Run Percentage | 9.42e-01 ± 1.96e-02 | 0.29 ± 0.31 | 0.98 ± 0.01 |
| Low Grey Level Run Emphasis | 1.08e-02 ± 1.35e-02 | 14.27 ± 16.17 | 0.99 ± 0.00 |
| high Grey Level Run Emphasis | 3.47e+02 ± 8.93e+01 | 2.59 ± 2.24 | 0.99 ± 0.00 |
| Short Run Low Grey Level Emphasis | 1.02e-02 ± 1.30e-02 | 14.32 ± 16.57 | 0.99 ± 0.01 |
| Short Run High Grey Level Emphasis | 3.30e+02 ± 8.35e+01 | 2.73 ± 2.50 | 0.99 ± 0.00 |
| Long Run Low Grey Level Emphasis | 1.34e-02 ± 1.57e-02 | 13.81 ± 15.46 | 0.98 ± 0.01 |
| Long Run High Grey Level Emphasis | 4.29e+02 ± 1.24e+02 | 2.66 ± 2.21 | 0.99 ± 0.00 |

- 1. **Soft-tissues sarcoma dataset**

**Table 31** First-Order Statistics (FOS) features for the patients of the Soft-Tissues Sarcomas (STS) dataset. In the table, the bin-independent features are presented. The first column shows the mean and standard deviations of the original radiomic features computed on all the patients. The mean and standard deviation of both percentage difference and Intra-class Correlation Coefficient (ICC) are shown for small entity rotations.

| **FIRST-ORDER STATISTICS (BIN-INDEPENDENT)** | | | |
| --- | --- | --- | --- |
| **Feature name** | **Original value** | **Diff %** | **ICC_SMALL_** |
| Signal Energy | 1.34e+11 ± 1.11e+11 | 11.73 ± 4.89 | 0.98 ± 0.00 |
| Signal Kurtosis | 3.17e+00 ± 1.05e+00 | 3.62 ± 2.91 | 0.98 ± 0.01 |
| Signal Mad | 4.37e+02 ± 1.26e+02 | 4.52 ± 3.93 | 0.98 ± 0.00 |
| Signal Max | 3.41e+03 ± 3.80e+02 | 1.34 ± 3.55 | 0.95 ± 0.01 |
| Signal Mean | 1.48e+03 ± 3.20e+02 | 2.92 ± 2.30 | 0.99 ± 0.00 |
| Signal Median | 1.46e+03 ± 4.06e+02 | 2.82 ± 3.13 | 0.99 ± 0.00 |
| Signal Min | 6.93e+00 ± 1.80e+01 | 3.03e+11 ± 1.82e+12 | 0.86 ± 0.14 |
| Signal Range | 3.40e+03 ± 3.87e+02 | 1.43 ± 3.52 | 0.95 ± 0.01 |
| Signal RMS | 1.58e+03 ± 3.30e+02 | 2.18 ± 1.95 | 0.99 ± 0.00 |
| Signal Skewness | 2.47e-01 ± 6.00e-01 | 432.43 ± 1058.90 | 0.96 ± 0.00 |
| Signal STD | 5.35e+02 ± 1.26e+02 | 4.68 ± 4.24 | 0.97 ± 0.00 |
| Signal Variance | 3.01e+05 ± 1.50e+05 | 9.71 ± 9.06 | 0.97 ± 0.00 |

**Table 32** First-Order Statistics (FOS) features for the patients of the Soft-Tissues Sarcomas (STS) dataset. In the table, the bin-dependent features calculated with a 16-bins discretization are presented. The first column shows the mean and standard deviations of the original radiomic features computed on all the patients. The mean and standard deviation of both percentage difference and Intra-class Correlation Coefficient (ICC) are shown for small entity rotations. Features for which mean ICC_SMALL_ is less than 0.78 (unstable features) are highlighted in red.

| **FIRST-ORDER STATISTICS (16-BINS DISCRETIZATION)** | | | |
| --- | --- | --- | --- |
| **Feature name** | **Original value** | **Diff %** | **ICC_SMALL_** |
| Histogram Entropy | 2.99e+00 ± 2.61e-01 | 1.78 ± 1.33 | 0.97 ± 0.00 |
| Histogram Kurtosis | 6.74e+00 ± 3.81e+00 | 4.70 ± 2.71 | 1.00 ± 0.00 |
| Histogram Mad | 4.39e-02 ± 2.56e-03 | 2.13 ± 1.43 | 0.92 ± 0.01 |
| Histogram Max | 2.34e-01 ± 6.81e-02 | 3.52 ± 2.62 | 0.99 ± 0.00 |
| Histogram Mean | 3.13e-02 ± 0.00e+00 | 0.00 ± 0.00 | 1.00 ± 0.00 |
| Histogram Median | 2.07e-05 ± 6.82e-05 | 0.00 ± 0.00 | 0.97 ± 0.03 |
| Histogram Min | 0.00e+00 ± 0.00e+00 | 0.00 ± 0.00 | 1.00 ± 0.00 |
| Histogram Range | 2.34e-01 ± 6.81e-02 | 3.52 ± 2.62 | 0.99 ± 0.00 |
| Histogram RMS | 6.89e-02 ± 7.77e-03 | 1.71 ± 1.24 | 0.98 ± 0.00 |
| Histogram Skewness | 2.10e+00 ± 6.69e-01 | 2.97 ± 1.92 | 0.99 ± 0.00 |
| Histogram STD | 6.23e-02 ± 8.91e-03 | 2.21 ± 1.60 | 0.98 ± 0.00 |
| Histogram Uniformity | 1.54e-01 ± 3.44e-02 | 3.39 ± 2.43 | 0.98 ± 0.00 |
| Histogram Variance | 3.96e-03 ± 1.11e-03 | 4.35 ± 3.12 | 0.98 ± 0.00 |
| Histogram TotalFrequency | 4.90e+04 ± 3.69e+04 | 8.06 ± 2.55 | 0.99 ± 0.00 |
| Signal Quantile 0.01 | 3.39e+02 ± 1.51e+02 | 40.13 ± 19.99 | 0.48 ± 0.06 |
| Signal Quantile 0.1 | 8.17e+02 ± 2.06e+02 | 9.52 ± 8.33 | 0.88 ± 0.00 |
| Signal Quantile 0.2 | 1.00e+03 ± 2.41e+02 | 4.63 ± 4.04 | 0.96 ± 0.00 |
| Signal Quantile 0.3 | 1.15e+03 ± 2.81e+02 | 3.74 ± 3.51 | 0.98 ± 0.01 |
| Signal Quantile 0.4 | 1.30e+03 ± 3.37e+02 | 3.18 ± 3.31 | 0.98 ± 0.01 |
| Signal Quantile 0.5 | 1.46e+03 ± 4.02e+02 | 2.80 ± 3.14 | 0.99 ± 0.00 |
| Signal Quantile 0.6 | 1.61e+03 ± 4.32e+02 | 2.33 ± 2.22 | 0.99 ± 0.00 |
| Signal Quantile 0.7 | 1.77e+03 ± 4.52e+02 | 1.69 ± 1.45 | 1.00 ± 0.00 |
| Signal Quantile 0.8 | 1.95e+03 ± 4.41e+02 | 1.63 ± 1.83 | 1.00 ± 0.00 |
| Signal Quantile 0.9 | 2.20e+03 ± 4.12e+02 | 1.37 ± 2.16 | 1.00 ± 0.00 |
| Signal Quantile 0.99 | 2.74e+03 ± 3.34e+02 | 1.20 ± 2.11 | 0.99 ± 0.01 |

**Table 33** First-Order Statistics (FOS) features for the patients of the Soft-Tissues Sarcomas (STS) dataset. In the table, the bin-dependent features calculated with a 32-bins discretization are presented. The first column shows the mean and standard deviations of the original radiomic features computed on all the patients. The mean and standard deviation of both percentage difference and Intra-class Correlation Coefficient (ICC) are shown for small entity rotations. Features for which mean ICC_SMALL_ is less than 0.7 (unstable features) are highlighted in red.

| **FIRST-ORDER STATISTICS (32-BINS DISCRETIZATION)** | | | |
| --- | --- | --- | --- |
| **Feature name** | **Original value** | **Diff %** | **ICC_SMALL_** |
| Histogram Entropy | 3.97e+00 ± 2.64e-01 | 1.37 ± 1.01 | 0.97 ± 0.00 |
| Histogram Kurtosis | 7.13e+00 ± 4.09e+00 | 4.99 ± 3.36 | 1.00 ± 0.00 |
| Histogram Mad | 2.21e-02 ± 1.30e-03 | 2.15 ± 1.50 | 0.92 ± 0.01 |
| Histogram Max | 1.26e-01 ± 4.04e-02 | 3.91 ± 2.92 | 0.99 ± 0.00 |
| Histogram Mean | 1.56e-02 ± 0.00e+00 | 0.00 ± 0.00 | 1.00 ± 0.00 |
| Histogram Median | 6.08e-06 ± 2.23e-05 | 0.00 ± 0.00 | 1.00 ± 0.00 |
| Histogram Min | 0.00e+00 ± 0.00e+00 | 0.00 ± 0.00 | 1.00 ± 0.00 |
| Histogram Range | 1.26e-01 ± 4.04e-02 | 3.91 ± 2.92 | 0.99 ± 0.00 |
| Histogram RMS | 3.49e-02 ± 4.06e-03 | 1.75 ± 1.28 | 0.98 ± 0.00 |
| Histogram Skewness | 2.16e+00 ± 6.99e-01 | 3.09 ± 2.03 | 0.99 ± 0.00 |
| Histogram STD | 3.14e-02 ± 4.59e-03 | 2.24 ± 1.65 | 0.98 ± 0.00 |
| Histogram Uniformity | 7.89e-02 ± 1.83e-02 | 3.46 ± 2.51 | 0.98 ± 0.00 |
| Histogram Variance | 1.00e-03 ± 2.90e-04 | 4.42 ± 3.20 | 0.98 ± 0.00 |
| Histogram TotalFrequency | 4.90e+04 ± 3.69e+04 | 8.06 ± 2.55 | 0.99 ± 0.00 |
| Signal Quantile 0.01 | 3.59e+02 ± 1.62e+02 | 39.59 ± 20.10 | 0.47 ± 0.04 |
| Signal Quantile 0.1 | 8.33e+02 ± 1.97e+02 | 8.58 ± 8.02 | 0.89 ± 0.01 |
| Signal Quantile 0.2 | 1.01e+03 ± 2.40e+02 | 4.54 ± 4.14 | 0.96 ± 0.00 |
| Signal Quantile 0.3 | 1.15e+03 ± 2.82e+02 | 3.64 ± 3.45 | 0.98 ± 0.01 |
| Signal Quantile 0.4 | 1.30e+03 ± 3.38e+02 | 3.18 ± 3.41 | 0.98 ± 0.01 |
| Signal Quantile 0.5 | 1.46e+03 ± 4.04e+02 | 2.80 ± 3.11 | 0.99 ± 0.00 |
| Signal Quantile 0.6 | 1.61e+03 ± 4.37e+02 | 2.35 ± 2.27 | 0.99 ± 0.00 |
| Signal Quantile 0.7 | 1.77e+03 ± 4.58e+02 | 1.80 ± 1.53 | 1.00 ± 0.00 |
| Signal Quantile 0.8 | 1.94e+03 ± 4.46e+02 | 1.60 ± 1.74 | 1.00 ± 0.00 |
| Signal Quantile 0.9 | 2.19e+03 ± 4.11e+02 | 1.34 ± 2.02 | 1.00 ± 0.00 |
| Signal Quantile 0.99 | 2.71e+03 ± 3.26e+02 | 1.25 ± 2.15 | 0.99 ± 0.01 |

**Table 34** First-Order Statistics (FOS) features for the patients of the Soft-Tissues Sarcomas (STS) dataset. In the table, the bin-dependent features calculated with a 64-bins discretization are presented. The first column shows the mean and standard deviations of the original radiomic features computed on all the patients. The mean and standard deviation of both percentage difference and Intra-class Correlation Coefficient (ICC) are shown for small entity rotation. Features for which mean ICC_SMALL_ is less than 0.7 (unstable features) are highlighted in red.

| **FIRST-ORDER STATISTICS (64-BINS DISCRETIZATION)** | | | |
| --- | --- | --- | --- |
| **Feature name** | **Original value** | **Diff %** | **ICC_SMALL_** |
| Histogram Entropy | 4.96e+00 ± 2.65e-01 | 1.11 ± 0.80 | 0.97 ± 0.00 |
| Histogram Kurtosis | 7.30e+00 ± 4.33e+00 | 5.05 ± 3.53 | 1.00 ± 0.00 |
| Histogram Mad | 1.10e-02 ± 6.48e-04 | 2.13 ± 1.49 | 0.92 ± 0.01 |
| Histogram Max | 6.49e-02 ± 2.11e-02 | 3.84 ± 3.13 | 0.99 ± 0.00 |
| Histogram Mean | 7.81e-03 ± 0.00e+00 | 0.00 ± 0.00 | 1.00 ± 0.00 |
| Histogram Median | 2.60e-06 ± 9.31e-06 | 0.00 ± 0.00 | 1.00 ± 0.00 |
| Histogram Min | 0.00e+00 ± 0.00e+00 | 0.00 ± 0.00 | 1.00 ± 0.00 |
| Histogram Range | 6.49e-02 ± 2.11e-02 | 3.84 ± 3.13 | 0.99 ± 0.00 |
| Histogram RMS | 1.75e-02 ± 2.06e-03 | 1.77 ± 1.28 | 0.98 ± 0.00 |
| Histogram Skewness | 2.19e+00 ± 7.20e-01 | 3.11 ± 2.04 | 0.99 ± 0.00 |
| Histogram STD | 1.57e-02 ± 2.32e-03 | 2.26 ± 1.64 | 0.98 ± 0.00 |
| Histogram Uniformity | 3.97e-02 ± 9.31e-03 | 3.49 ± 2.50 | 0.98 ± 0.00 |
| Histogram Variance | 2.51e-04 ± 7.33e-05 | 4.44 ± 3.20 | 0.98 ± 0.00 |
| Histogram TotalFrequency | 4.90e+04 ± 3.69e+04 | 8.06 ± 2.55 | 0.99 ± 0.00 |
| Signal Quantile 0.01 | 3.62e+02 ± 1.63e+02 | 39.75 ± 19.80 | 0.47 ± 0.04 |
| Signal Quantile 0.1 | 8.35e+02 ± 1.96e+02 | 8.38 ± 8.01 | 0.89 ± 0.01 |
| Signal Quantile 0.2 | 1.01e+03 ± 2.39e+02 | 4.45 ± 4.10 | 0.96 ± 0.00 |
| Signal Quantile 0.3 | 1.15e+03 ± 2.82e+02 | 3.60 ± 3.45 | 0.98 ± 0.01 |
| Signal Quantile 0.4 | 1.30e+03 ± 3.39e+02 | 3.20 ± 3.41 | 0.98 ± 0.01 |
| Signal Quantile 0.5 | 1.46e+03 ± 4.05e+02 | 2.81 ± 3.12 | 0.99 ± 0.00 |
| Signal Quantile 0.6 | 1.61e+03 ± 4.39e+02 | 2.34 ± 2.28 | 0.99 ± 0.00 |
| Signal Quantile 0.7 | 1.76e+03 ± 4.58e+02 | 1.81 ± 1.56 | 1.00 ± 0.00 |
| Signal Quantile 0.8 | 1.94e+03 ± 4.47e+02 | 1.61 ± 1.78 | 1.00 ± 0.00 |
| Signal Quantile 0.9 | 2.19e+03 ± 4.10e+02 | 1.35 ± 2.00 | 1.00 ± 0.00 |
| Signal Quantile 0.99 | 2.71e+03 ± 3.26e+02 | 1.27 ± 2.17 | 0.98 ± 0.01 |

**Table 35** Grey Level Co-occurrence Matrix (GLCM) features for the patients of the Soft-Tissues Sarcomas (STS) dataset. In the table, the features computed for 16-bins histogram discretization are presented. The first column shows the mean and standard deviations of the original radiomic features computed on all the patients. The mean and standard deviation of both percentage difference and Intra-class Correlation Coefficient (ICC) are shown for small entity rotations. Values are shown with scientific format only for the original features. The numbers are approximated to the second decimal significant digit.

| **GREY LEVEL CO-OCCURRENCE MATRIX (16-BINS DISCRETIZATION)** | | | |
| --- | --- | --- | --- |
| **Feature name** | **Original value** | **Diff %** | **ICC_SMALL_** |
| Autocorrelation | 3.49e+01 ± 1.71e+01 | 4.49 ± 4.17 | 0.99 ± 0.00 |
| Cluster Prominence | 3.38e+04 ± 3.17e+04 | 6.67 ± 7.97 | 1.00 ± 0.00 |
| Cluster Shade | 2.08e+03 ± 1.49e+03 | 5.61 ± 5.96 | 1.00 ± 0.00 |
| Cluster Tendency | 1.41e+02 ± 6.84e+01 | 4.37 ± 4.13 | 0.99 ± 0.00 |
| Contrast | 3.65e+00 ± 1.20e+00 | 4.49 ± 3.69 | 0.99 ± 0.00 |
| Correlation | 6.98e+03 ± 6.12e+03 | 5.10 ± 4.44 | 0.99 ± 0.00 |
| Difference Entropy | 2.17e+00 ± 1.76e-01 | 1.24 ± 1.03 | 0.98 ± 0.00 |
| Dissimilarity | 1.34e+00 ± 2.20e-01 | 2.24 ± 1.83 | 0.99 ± 0.00 |
| Energy | 3.79e-02 ± 1.71e-02 | 4.72 ± 3.68 | 0.99 ± 0.00 |
| Entropy | 5.54e+00 ± 4.66e-01 | 1.33 ± 1.15 | 0.98 ± 0.00 |
| Homogeneity | 5.69e-01 ± 3.69e-02 | 0.81 ± 0.77 | 0.99 ± 0.00 |
| Homogeneity2 | 5.23e-01 ± 4.53e-02 | 1.09 ± 1.05 | 0.99 ± 0.00 |
| IMOC1 | -1.16e-01 ± 4.84e-02 | 9.21 ± 8.53 | 0.98 ± 0.00 |
| IMOC2 | 6.78e-01 ± 1.23e-01 | 3.75 ± 3.85 | 0.97 ± 0.00 |
| Inertia | 1.00e+00 ± 1.83e-05 | 0.00 ± 0.00 | 0.99 ± 0.00 |
| Inverse Difference moment | 9.95e-01 ± 8.44e-04 | 0.01 ± 0.01 | 0.99 ± 0.00 |
| Inverse Difference moment2 | 4.38e-01 ± 2.67e-02 | 0.85 ± 0.73 | 0.98 ± 0.01 |
| Inverse Variance | 9.61e-02 ± 5.22e-02 | 4.82 ± 4.65 | 1.00 ± 0.00 |
| Max Probability | 1.10e+01 ± 2.72e+00 | 2.76 ± 2.41 | 0.99 ± 0.00 |
| Sum Average | 3.76e+00 ± 3.32e-01 | 1.61 ± 1.13 | 0.98 ± 0.00 |
| Sum Entropy | 3.65e+00 ± 1.20e+00 | 4.49 ± 3.69 | 0.99 ± 0.00 |

**Table 36** Grey Level Co-occurrence Matrix (GLCM) features for the patients of the Soft-Tissues Sarcomas (STS) dataset. In the table, the features computed for 32-bins histogram discretization are presented. The first column shows the mean and standard deviations of the original radiomic features computed on all the patients. The mean and standard deviation of both percentage difference and Intra-class Correlation Coefficient (ICC) are shown for small entity rotations. Values are shown with scientific format only for the original features.

| **GREY LEVEL CO-OCCURRENCE MATRIX (32-BINS DISCRETIZATION)** | | | |
| --- | --- | --- | --- |
| **Feature name** | **Original value** | **Diff %** | **ICC_SMALL_** |
| Autocorrelation | 1.51e+02 ± 7.13e+01 | 4.33 ± 3.98 | 0.99 ± 0.00 |
| Cluster Prominence | 6.23e+05 ± 5.66e+05 | 6.48 ± 7.53 | 1.00 ± 0.00 |
| Cluster Shade | 1.87e+04 ± 1.29e+04 | 5.42 ± 5.62 | 0.99 ± 0.00 |
| Cluster Tendency | 6.15e+02 ± 2.86e+02 | 4.20 ± 3.92 | 0.99 ± 0.00 |
| Contrast | 1.41e+01 ± 4.81e+00 | 4.69 ± 3.83 | 0.99 ± 0.01 |
| Correlation | 1.21e+05 ± 1.02e+05 | 5.06 ± 4.49 | 0.99 ± 0.01 |
| Difference Entropy | 3.00e+00 ± 1.97e-01 | 0.99 ± 0.83 | 0.98 ± 0.00 |
| Dissimilarity | 2.68e+00 ± 4.40e-01 | 2.27 ± 1.80 | 0.99 ± 0.00 |
| Energy | 1.04e-02 ± 4.87e-03 | 5.01 ± 4.09 | 0.99 ± 0.00 |
| Entropy | 7.46e+00 ± 4.81e-01 | 1.02 ± 0.87 | 0.98 ± 0.00 |
| Homogeneity | 4.21e-01 ± 3.37e-02 | 1.04 ± 0.99 | 0.98 ± 0.00 |
| Homogeneity2 | 3.45e-01 ± 3.99e-02 | 1.47 ± 1.45 | 0.99 ± 0.01 |
| IMOC1 | -9.54e-02 ± 3.82e-02 | 10.28 ± 9.75 | 0.97 ± 0.00 |
| IMOC2 | 7.03e-01 ± 1.12e-01 | 3.50 ± 3.39 | 0.97 ± 0.00 |
| Inertia | 1.00e+00 ± 4.59e-06 | 0.00 ± 0.00 | 0.98 ± 0.00 |
| Inverse Difference moment | 9.97e-01 ± 4.25e-04 | 0.01 ± 0.00 | 0.99 ± 0.00 |
| Inverse Difference moment2 | 3.34e-01 ± 3.22e-02 | 1.29 ± 1.11 | 0.99 ± 0.00 |
| Inverse Variance | 3.15e-02 ± 1.80e-02 | 5.28 ± 5.36 | 0.99 ± 0.00 |
| Max Probability | 2.30e+01 ± 5.44e+00 | 2.64 ± 2.29 | 0.99 ± 0.00 |
| Sum Average | 4.74e+00 ± 3.36e-01 | 1.31 ± 0.90 | 0.98 ± 0.00 |
| Sum Entropy | 1.41e+01 ± 4.81e+00 | 4.69 ± 3.83 | 0.99 ± 0.01 |

**Table 37** Grey Level Co-occurrence Matrix (GLCM) features for the patients of the Soft-Tissues Sarcomas (STS) dataset. In the table, the features computed for 64-bins histogram discretization are presented. The first column shows the mean and standard deviations of the original radiomic features computed on all the patients. The mean and standard deviation of both percentage difference and Intra-class Correlation Coefficient (ICC) are shown for small entity rotations. Values are shown with scientific format only for the original features. The numbers are approximated to the second decimal significant digit.

| **GREY LEVEL CO-OCCURRENCE MATRIX (64-BINS DISCRETIZATION)** | | | |
| --- | --- | --- | --- |
| **Feature name** | **Original value** | **Diff %** | **ICC_SMALL_** |
| Autocorrelation | 6.27e+02 ± 2.90e+02 | 4.25 ± 3.89 | 0.99 ± 0.00 |
| Cluster Prominence | 1.06e+07 ± 9.50e+06 | 6.39 ± 7.34 | 1.00 ± 0.00 |
| Cluster Shade | 1.58e+05 ± 1.07e+05 | 5.34 ± 5.48 | 0.99 ± 0.00 |
| Cluster Tendency | 2.56e+03 ± 1.17e+03 | 4.13 ± 3.83 | 0.99 ± 0.00 |
| Contrast | 5.61e+01 ± 1.92e+01 | 4.76 ± 3.88 | 0.99 ± 0.01 |
| Correlation | 2.01e+06 ± 1.67e+06 | 5.04 ± 4.49 | 0.99 ± 0.01 |
| Difference Entropy | 3.92e+00 ± 2.08e-01 | 0.80 ± 0.67 | 0.98 ± 0.00 |
| Dissimilarity | 5.36e+00 ± 8.79e-01 | 2.29 ± 1.81 | 0.99 ± 0.00 |
| Energy | 2.72e-03 ± 1.29e-03 | 5.02 ± 4.14 | 0.99 ± 0.00 |
| Entropy | 9.39e+00 ± 4.94e-01 | 0.80 ± 0.68 | 0.98 ± 0.00 |
| Homogeneity | 2.92e-01 ± 2.76e-02 | 1.23 ± 1.24 | 0.98 ± 0.01 |
| Homogeneity2 | 2.05e-01 ± 2.85e-02 | 1.75 ± 1.87 | 0.98 ± 0.01 |
| IMOC1 | -8.71e-02 ± 2.84e-02 | 9.99 ± 8.54 | 0.95 ± 0.00 |
| IMOC2 | 7.42e-01 ± 9.08e-02 | 3.16 ± 2.65 | 0.95 ± 0.01 |
| Inertia | 1.00e+00 ± 1.18e-06 | 0.00 ± 0.00 | 0.97 ± 0.02 |
| Inverse Difference moment | 9.99e-01 ± 2.14e-04 | 0.00 ± 0.00 | 0.99 ± 0.00 |
| Inverse Difference moment2 | 2.09e-01 ± 2.79e-02 | 1.65 ± 1.62 | 0.99 ± 0.01 |
| Inverse Variance | 9.18e-03 ± 5.00e-03 | 6.15 ± 5.89 | 0.99 ± 0.00 |
| Max Probability | 4.70e+01 ± 1.09e+01 | 2.58 ± 2.23 | 0.99 ± 0.00 |
| Sum Average | 5.74e+00 ± 3.37e-01 | 1.08 ± 0.74 | 0.98 ± 0.00 |
| Sum Entropy | 5.61e+01 ± 1.92e+01 | 4.76 ± 3.88 | 0.99 ± 0.01 |

**Table 38** Grey Level Run Length Matrix (GLRLM) features for the patients of the Soft-Tissues Sarcomas (STS) dataset. In the table, the features computed for 16-bins histogram discretization are presented. The first column shows the mean and standard deviations of the original radiomic features computed on all the patients. The mean and standard deviation of both percentage difference and Intra-class Correlation Coefficient (ICC) are shown for small rotations. Values are shown with scientific format only for the original features. The numbers are approximated to the second decimal significant digit.

| **GREY LEVEL RUN LENGTH MATRIX (16-BINS DISCRETIZATION)** | | | |
| --- | --- | --- | --- |
| **Feature name** | **Original value** | **Diff %** | **ICC_SMALL_** |
| Short Run Emphasis | 9.90e-01 ± 7.22e-03 | 0.05 ± 0.05 | 1.00 ± 0.00 |
| Long Run Emphasis | 1.04e+00 ± 3.57e-02 | 0.20 ± 0.19 | 1.00 ± 0.00 |
| Grey Level Non-Uniformity | 4.82e+03 ± 3.52e+03 | 10.29 ± 3.73 | 0.99 ± 0.00 |
| Run Length Non-Uniformity | 3.49e+04 ± 2.49e+04 | 7.76 ± 2.82 | 0.99 ± 0.00 |
| Run Percentage | 7.47e-01 ± 3.85e-02 | 0.63 ± 0.57 | 0.99 ± 0.00 |
| Low Grey Level Run Emphasis | 5.00e-02 ± 2.20e-02 | 27.59 ± 18.13 | 0.82 ± 0.02 |
| high Grey Level Run Emphasis | 4.75e+01 ± 1.66e+01 | 4.41 ± 3.47 | 0.99 ± 0.00 |
| Short Run Low Grey Level Emphasis | 4.96e-02 ± 2.16e-02 | 27.71 ± 18.23 | 0.82 ± 0.02 |
| Short Run High Grey Level Emphasis | 4.70e+01 ± 1.61e+01 | 4.45 ± 3.50 | 0.99 ± 0.00 |
| Long Run Low Grey Level Emphasis | 5.20e-02 ± 2.38e-02 | 27.08 ± 17.74 | 0.84 ± 0.01 |
| Long Run High Grey Level Emphasis | 4.97e+01 ± 1.93e+01 | 4.28 ± 3.38 | 0.99 ± 0.00 |

**Table 39** Grey Level Run Length Matrix (GLRLM) features for the patients of the Soft-Tissues Sarcomas (STS) dataset. In the table, the features computed for 32-bins histogram discretization are presented. The first column shows the mean and standard deviations of the original radiomic features computed on all the patients. The mean and standard deviation of both percentage difference and Intra-class Correlation Coefficient (ICC) are shown for small rotations. Values are shown with scientific format only for the original features. The numbers are approximated to the second decimal significant digit.

| **GREY LEVEL RUN LENGTH MATRIX (32-BINS DISCRETIZATION)** | | | |
| --- | --- | --- | --- |
| **Feature name** | **Original value** | **Diff %** | **ICC_SMALL_** |
| Short Run Emphasis | 9.82e-01 ± 7.84e-03 | 0.06 ± 0.06 | 0.99 ± 0.00 |
| Long Run Emphasis | 1.08e+00 ± 3.75e-02 | 0.27 ± 0.27 | 0.99 ± 0.00 |
| Grey Level Non-Uniformity | 3.01e+03 ± 2.23e+03 | 10.32 ± 3.78 | 0.99 ± 0.00 |
| Run Length Non-Uniformity | 3.97e+04 ± 2.85e+04 | 7.88 ± 2.74 | 0.99 ± 0.00 |
| Run Percentage | 8.64e-01 ± 2.28e-02 | 0.35 ± 0.34 | 0.98 ± 0.01 |
| Low Grey Level Run Emphasis | 1.63e-02 ± 8.58e-03 | 44.00 ± 29.00 | 0.73 ± 0.01 |
| high Grey Level Run Emphasis | 1.78e+02 ± 6.79e+01 | 4.36 ± 3.62 | 0.99 ± 0.00 |
| Short Run Low Grey Level Emphasis | 1.61e-02 ± 8.45e-03 | 44.24 ± 29.23 | 0.73 ± 0.01 |
| Short Run High Grey Level Emphasis | 1.74e+02 ± 6.56e+01 | 4.40 ± 3.65 | 0.99 ± 0.00 |
| Long Run Low Grey Level Emphasis | 1.74e-02 ± 9.30e-03 | 42.87 ± 28.11 | 0.74 ± 0.01 |
| Long Run High Grey Level Emphasis | 1.92e+02 ± 7.95e+01 | 4.15 ± 3.48 | 0.99 ± 0.00 |

**Table 40** Grey Level Run Length Matrix (GLRLM) features for the patients of the Soft-Tissues Sarcomas (STS) dataset. In the table, the features computed for 64-bins histogram discretization are presented. The first column shows the mean and standard deviations of the original radiomic features computed on all the patients. The mean and standard deviation of both percentage difference and Intra-class Correlation Coefficient (ICC) are shown for small rotations. Values are shown with scientific format only for the original features. The numbers are approximated to the second decimal significant digit. Features for which mean ICC_SMALL_ is less than 0.7 (unstable features) are highlighted in red.

| **GREY LEVEL RUN LENGTH MATRIX (64-BINS DISCRETIZATION)** | | | |
| --- | --- | --- | --- |
| **Feature name** | **Original value** | **Diff %** | **ICC_SMALL_** |
| Short Run Emphasis | 9.48e-01 ± 1.91e-02 | 0.12 ± 0.14 | 1.00 ± 0.00 |
| Long Run Emphasis | 1.24e+00 ± 9.39e-02 | 0.50 ± 0.50 | 1.00 ± 0.00 |
| Grey Level Non-Uniformity | 1.69e+03 ± 1.26e+03 | 10.42 ± 3.81 | 0.99 ± 0.00 |
| Run Length Non-Uniformity | 3.91e+04 ± 2.80e+04 | 8.02 ± 2.78 | 0.99 ± 0.00 |
| Run Percentage | 9.30e-01 ± 1.22e-02 | 0.19 ± 0.19 | 0.98 ± 0.01 |
| Low Grey Level Run Emphasis | 5.49e-03 ± 3.55e-03 | 65.95 ± 43.19 | 0.67 ± 0.01 |
| high Grey Level Run Emphasis | 6.86e+02 ± 2.75e+02 | 4.31 ± 3.69 | 0.99 ± 0.00 |
| Short Run Low Grey Level Emphasis | 5.28e-03 ± 3.43e-03 | 66.17 ± 43.47 | 0.67 ± 0.02 |
| Short Run High Grey Level Emphasis | 6.51e+02 ± 2.55e+02 | 4.38 ± 3.75 | 0.99 ± 0.00 |
| Long Run Low Grey Level Emphasis | 6.50e-03 ± 4.12e-03 | 64.43 ± 42.69 | 0.66 ± 0.00 |
| Long Run High Grey Level Emphasis | 8.48e+02 ± 3.86e+02 | 4.00 ± 3.45 | 0.99 ± 0.00 |

1. **TABLES OF FEATURES (SCALING)**

In this section the tables with the percentage variation of radiomic features and ICC after the application of scaling on both the x and y direction (in-plane directions) is presented. The stability in a range of scaling factors ranging from 0.5 to 1.5 with a step of 0.1. Considering the maximum entity scaling (0.5 and 1.5), it was observed that the ICC was remained high. This is because even for such scaling factors, the overlap between the original and transformed ROI is high. For this reason, those transformations were not used for the features relevance analysis. Therefore, only a stability analysis was performed, using the minimum entity scaling, was used (0.9 and 1.1). If the ICC_MEAN_ of the minimum intensity scaling for a particular feature is below 0.7 (threshold taken from the main paper), we consider the feature unstable. Although, the ICC obtained for large transformation (ICC_LARGE_) are not used for the relevance analysis, they are reported in the following tables, for the sake of completeness.

- 1. **Oropharyngeal cancers dataset**

**Table 41** First-Order Statistics (FOS) features for the patients of the OroPharyngeal Cancers (OPC) dataset. In the table, the bin-independent features are presented. The first column shows the mean and standard deviations of the original radiomic features computed on all the patients. The mean and standard deviation of both percentage difference and Intra-class Correlation Coefficient (ICC) are shown for both minimum and maximum entity scaling. Values are shown with scientific format only for the original features. The numbers are approximated to the second decimal significant digit. Features for which mean ICC_SMALL_ is less than 0.78 (unstable features) are highlighted in red.

| **FIRST-ORDER STATISTICS (BIN-INDEPENDENT)** | | | | | |
| --- | --- | --- | --- | --- | --- |
| **Feature name** | **Original value** | **Diff %**  **(min. entity)** | **Diff %**  **(max. entity)** | **ICC_SMALL_** | **ICC_LARGE_** |
| Signal Energy | 2.37e+09 ± 1.92e+09 | 21.61 ± 9.42 | 101.58 ± 28.09 | 0.92 ± 0.06 | 0.36 ± 0.25 |
| Signal Kurtosis | 3.24e+00 ± 7.17e-01 | 7.78 ± 8.49 | 34.06 ± 69.35 | 0.89 ± 0.10 | 0.34 ± 0.47 |
| Signal Mad | 3.01e+02 ± 5.77e+01 | 3.93 ± 3.67 | 15.80 ± 15.62 | 0.96 ± 0.04 | 0.52 ± 0.44 |
| Signal Max | 2.41e+03 ± 4.14e+02 | 1.73 ± 3.61 | 12.13 ± 10.98 | 0.97 ± 0.03 | 0.63 ± 0.26 |
| Signal Mean | 1.05e+03 ± 1.59e+02 | 2.27 ± 1.80 | 8.53 ± 6.32 | 0.98 ± 0.02 | 0.78 ± 0.18 |
| Signal Median | 9.83e+02 ± 1.49e+02 | 3.43 ± 3.57 | 11.10 ± 8.91 | 0.95 ± 0.01 | 0.70 ± 0.03 |
| Signal Min | 1.01e+02 ± 1.24e+02 | 389.62 ± 1992.48 | 578.52 ± 2055.04 | 0.67 ± 0.01 | 0.30 ± 0.04 |
| Signal Range | 2.31e+03 ± 4.54e+02 | 3.90 ± 5.51 | 19.03 ± 15.65 | 0.93 ± 0.05 | 0.48 ± 0.29 |
| Signal RMS | 1.12e+03 ± 1.53e+02 | 2.27 ± 1.80 | 8.50 ± 7.00 | 0.97 ± 0.02 | 0.73 ± 0.24 |
| Signal Skewness | 5.13e-01 ± 3.61e-01 | 23.23 ± 21.84 | 81.97 ± 65.83 | 0.92 ± 0.05 | 0.47 ± 0.04 |
| Signal STD | 3.72e+02 ± 5.90e+01 | 3.27 ± 2.97 | 13.49 ± 13.93 | 0.97 ± 0.03 | 0.52 ± 0.37 |
| Signal Variance | 1.42e+05 ± 4.39e+04 | 6.40 ± 5.66 | 24.61 ± 20.78 | 0.97 ± 0.03 | 0.54 ± 0.35 |

**Table 42** First-Order Statistics (FOS) features for the patients of the OroPharyngeal Cancers (OPC) dataset. In the table, the bin-dependent features calculated with a 16-bins discretization are presented. The first column shows the mean and standard deviations of the original radiomic features computed on all the patients. The mean and standard deviation of both percentage difference and Intra-class Correlation Coefficient (ICC) are shown for both minimum and maximum entity scaling. Values are shown with scientific format only for the original features. The numbers are approximated to the second decimal significant digit.

| **FIRST-ORDER STATISTICS (16-BINS DISCRETIZATION)** | | | | | |
| --- | --- | --- | --- | --- | --- |
| **Feature name** | **Original value** | **Diff %**  **(min. entity)** | **Diff %**  **(max. entity)** | **ICC_SMALL_** | **ICC_LARGE_** |
| Histogram Entropy | 2.51e+00 ± 1.91e-01 | 2.74 ± 2.12 | 11.95 ± 9.85 | 0.91 ± 0.08 | 0.39 ± 0.27 |
| Histogram Kurtosis | 9.46e+00 ± 2.26e+00 | 11.79 ± 9.35 | 39.99 ± 26.95 | 0.83 ± 0.08 | 0.27 ± 0.08 |
| Histogram Mad | 4.86e-02 ± 1.24e-03 | 0.77 ± 0.76 | 3.51 ± 3.21 | 0.91 ± 0.07 | 0.37 ± 0.22 |
| Histogram Max | 3.17e-01 ± 5.13e-02 | 7.07 ± 5.07 | 28.66 ± 17.00 | 0.88 ± 0.08 | 0.28 ± 0.06 |
| Histogram Mean | 3.13e-02 ± 0.00e+00 | 0.00 ± 0.00 | 0.00 ± 0.00 | 1.00 ± 0.00 | 1.00 ± 0.00 |
| Histogram Median | 0.00e+00 ± 0.00e+00 | 0.00 ± 0.00 | 0.00 ± 0.00 | 1.00 ± 0.00 | 1.00 ± 0.00 |
| Histogram Min | 0.00e+00 ± 0.00e+00 | 0.00 ± 0.00 | 0.00 ± 0.00 | 1.00 ± 0.00 | 1.00 ± 0.00 |
| Histogram Range | 3.17e-01 ± 5.13e-02 | 7.07 ± 5.07 | 28.66 ± 17.00 | 0.88 ± 0.08 | 0.28 ± 0.06 |
| Histogram RMS | 8.12e-02 ± 6.28e-03 | 3.14 ± 2.61 | 12.87 ± 11.15 | 0.89 ± 0.10 | 0.38 ± 0.24 |
| Histogram Skewness | 2.67e+00 ± 3.72e-01 | 6.67 ± 5.35 | 22.43 ± 14.46 | 0.85 ± 0.08 | 0.31 ± 0.02 |
| Histogram STD | 7.61e-02 ± 6.90e-03 | 3.69 ± 3.06 | 14.99 ± 12.80 | 0.89 ± 0.10 | 0.38 ± 0.24 |
| Histogram Uniformity | 2.12e-01 ± 3.35e-02 | 6.39 ± 5.46 | 27.87 ± 27.98 | 0.89 ± 0.10 | 0.37 ± 0.28 |
| Histogram Variance | 5.83e-03 ± 1.08e-03 | 7.52 ± 6.47 | 32.79 ± 33.21 | 0.89 ± 0.10 | 0.37 ± 0.28 |
| Histogram TotalFrequency | 1.81e+03 ± 1.34e+03 | 18.47 ± 7.93 | 91.16 ± 15.58 | 0.94 ± 0.05 | 0.39 ± 0.24 |
| Signal Quantile 0.01 | 3.25e+02 ± 1.92e+02 | 11.29 ± 12.77 | 41.39 ± 36.55 | 0.97 ± 0.00 | 0.66 ± 0.29 |
| Signal Quantile 0.1 | 6.10e+02 ± 1.76e+02 | 2.53 ± 2.59 | 10.19 ± 11.90 | 0.99 ± 0.00 | 0.92 ± 0.03 |
| Signal Quantile 0.2 | 7.24e+02 ± 1.64e+02 | 1.79 ± 1.34 | 7.64 ± 6.51 | 1.00 ± 0.00 | 0.94 ± 0.01 |
| Signal Quantile 0.3 | 8.16e+02 ± 1.55e+02 | 1.94 ± 1.28 | 8.66 ± 6.30 | 0.99 ± 0.00 | 0.88 ± 0.01 |
| Signal Quantile 0.4 | 9.01e+02 ± 1.49e+02 | 2.28 ± 1.94 | 9.72 ± 7.20 | 0.98 ± 0.01 | 0.80 ± 0.02 |
| Signal Quantile 0.5 | 9.93e+02 ± 1.48e+02 | 3.02 ± 2.65 | 10.52 ± 7.98 | 0.96 ± 0.01 | 0.72 ± 0.01 |
| Signal Quantile 0.6 | 1.10e+03 ± 1.56e+02 | 3.59 ± 3.78 | 10.85 ± 8.18 | 0.92 ± 0.06 | 0.65 ± 0.21 |
| Signal Quantile 0.7 | 1.23e+03 ± 1.75e+02 | 3.33 ± 3.14 | 10.52 ± 8.59 | 0.93 ± 0.06 | 0.63 ± 0.33 |
| Signal Quantile 0.8 | 1.38e+03 ± 1.89e+02 | 2.76 ± 2.35 | 9.74 ± 9.36 | 0.96 ± 0.04 | 0.63 ± 0.41 |
| Signal Quantile 0.9 | 1.58e+03 ± 2.04e+02 | 2.14 ± 1.96 | 7.89 ± 9.37 | 0.97 ± 0.03 | 0.65 ± 0.40 |
| Signal Quantile 0.99 | 2.04e+03 ± 2.39e+02 | 1.55 ± 1.23 | 7.61 ± 8.54 | 0.99 ± 0.01 | 0.66 ± 0.32 |

**Table 43** First-Order Statistics (FOS) features for the patients of the OroPharyngeal Cancers (OPC) dataset. In the table, the bin-dependent features calculated with a 32-bins discretization are presented. The first column shows the mean and standard deviations of the original radiomic features computed on all the patients. The mean and standard deviation of both percentage difference and Intra-class Correlation Coefficient (ICC) are shown for both minimum and maximum entity scaling. Values are shown with scientific format only for the original features. The numbers are approximated to the second decimal significant digit.

| **FIRST-ORDER STATISTICS (32-BINS DISCRETIZATION)** | | | | | |
| --- | --- | --- | --- | --- | --- |
| **Feature name** | **Original value** | **Diff %**  **(min. entity)** | **Diff %**  **(max. entity)** | **ICC_SMALL_** | **ICC_LARGE_** |
| Histogram Entropy | 3.47e+00 ± 2.05e-01 | 2.21 ± 1.65 | 9.75 ± 8.04 | 0.91 ± 0.08 | 0.39 ± 0.24 |
| Histogram Kurtosis | 1.08e+01 ± 2.95e+00 | 13.47 ± 11.36 | 45.06 ± 31.57 | 0.81 ± 0.06 | 0.26 ± 0.10 |
| Histogram Mad | 2.44e-02 ± 6.61e-04 | 0.78 ± 0.73 | 3.90 ± 3.18 | 0.93 ± 0.06 | 0.40 ± 0.18 |
| Histogram Max | 1.84e-01 ± 3.69e-02 | 8.38 ± 5.82 | 33.31 ± 21.43 | 0.88 ± 0.06 | 0.30 ± 0.05 |
| Histogram Mean | 1.56e-02 ± 0.00e+00 | 0.00 ± 0.00 | 0.00 ± 0.00 | 1.00 ± 0.00 | 1.00 ± 0.00 |
| Histogram Median | 0.00e+00 ± 0.00e+00 | 0.00 ± 0.00 | 0.00 ± 0.00 | 1.00 ± 0.00 | 1.00 ± 0.00 |
| Histogram Min | 0.00e+00 ± 0.00e+00 | 0.00 ± 0.00 | 0.00 ± 0.00 | 1.00 ± 0.00 | 1.00 ± 0.00 |
| Histogram Range | 1.84e-01 ± 3.69e-02 | 8.38 ± 5.82 | 33.31 ± 21.43 | 0.88 ± 0.06 | 0.30 ± 0.05 |
| Histogram RMS | 4.14e-02 ± 3.43e-03 | 3.55 ± 2.95 | 14.63 ± 13.11 | 0.88 ± 0.10 | 0.36 ± 0.22 |
| Histogram Skewness | 2.83e+00 ± 4.32e-01 | 7.42 ± 6.20 | 24.71 ± 16.10 | 0.84 ± 0.07 | 0.30 ± 0.06 |
| Histogram STD | 3.86e-02 ± 3.73e-03 | 4.13 ± 3.42 | 16.88 ± 14.86 | 0.88 ± 0.10 | 0.36 ± 0.21 |
| Histogram Uniformity | 1.11e-01 ± 1.86e-02 | 7.24 ± 6.20 | 32.16 ± 34.18 | 0.88 ± 0.11 | 0.35 ± 0.26 |
| Histogram Variance | 1.51e-03 ± 2.95e-04 | 8.45 ± 7.25 | 37.50 ± 40.06 | 0.88 ± 0.11 | 0.35 ± 0.26 |
| Histogram TotalFrequency | 1.81e+03 ± 1.34e+03 | 18.47 ± 7.93 | 91.16 ± 15.58 | 0.94 ± 0.05 | 0.39 ± 0.24 |
| Signal Quantile 0.01 | 3.49e+02 ± 2.12e+02 | 11.61 ± 14.21 | 40.00 ± 35.97 | 0.97 ± 0.00 | 0.68 ± 0.32 |
| Signal Quantile 0.1 | 6.27e+02 ± 1.80e+02 | 2.37 ± 2.71 | 9.74 ± 11.11 | 1.00 ± 0.00 | 0.93 ± 0.03 |
| Signal Quantile 0.2 | 7.32e+02 ± 1.62e+02 | 1.70 ± 1.55 | 7.43 ± 6.75 | 1.00 ± 0.00 | 0.94 ± 0.01 |
| Signal Quantile 0.3 | 8.14e+02 ± 1.54e+02 | 1.83 ± 1.09 | 8.23 ± 5.75 | 0.99 ± 0.00 | 0.89 ± 0.00 |
| Signal Quantile 0.4 | 8.94e+02 ± 1.49e+02 | 2.33 ± 1.81 | 9.88 ± 7.61 | 0.98 ± 0.01 | 0.80 ± 0.06 |
| Signal Quantile 0.5 | 9.85e+02 ± 1.48e+02 | 3.35 ± 3.21 | 10.94 ± 8.49 | 0.95 ± 0.01 | 0.71 ± 0.03 |
| Signal Quantile 0.6 | 1.10e+03 ± 1.56e+02 | 3.78 ± 4.23 | 11.16 ± 8.41 | 0.91 ± 0.08 | 0.64 ± 0.22 |
| Signal Quantile 0.7 | 1.22e+03 ± 1.74e+02 | 3.36 ± 3.13 | 10.97 ± 9.10 | 0.93 ± 0.07 | 0.61 ± 0.36 |
| Signal Quantile 0.8 | 1.37e+03 ± 1.94e+02 | 2.70 ± 2.31 | 10.16 ± 10.12 | 0.96 ± 0.04 | 0.62 ± 0.44 |
| Signal Quantile 0.9 | 1.57e+03 ± 2.02e+02 | 2.13 ± 1.92 | 7.88 ± 9.70 | 0.97 ± 0.03 | 0.64 ± 0.42 |
| Signal Quantile 0.99 | 2.02e+03 ± 2.29e+02 | 1.95 ± 2.19 | 7.73 ± 8.29 | 0.98 ± 0.01 | 0.66 ± 0.34 |

**Table 44** First-Order Statistics (FOS) features for the patients of the OroPharyngeal Cancers (OPC) dataset. In the table, the bin-dependent features calculated with a 64-bins discretization are presented. The first column shows the mean and standard deviations of the original radiomic features computed on all the patients. The mean and standard deviation of both percentage difference and Intra-class Correlation Coefficient (ICC) are shown for both minimum and maximum entity scaling. Values are shown with scientific format only for the original features. The numbers are approximated to the second decimal significant digit.

| **FIRST-ORDER STATISTICS (64-BINS DISCRETIZATION)** | | | | | |
| --- | --- | --- | --- | --- | --- |
| **Feature name** | **Original value** | **Diff %**  **(min. entity)** | **Diff %**  **(max. entity)** | **ICC_SMALL_** | **ICC_LARGE_** |
| Histogram Entropy | 4.44e+00 ± 2.12e-01 | 1.84 ± 1.34 | 8.57 ± 6.90 | 0.91 ± 0.08 | 0.39 ± 0.23 |
| Histogram Kurtosis | 1.14e+01 ± 3.10e+00 | 13.20 ± 11.10 | 49.44 ± 38.10 | 0.82 ± 0.07 | 0.25 ± 0.06 |
| Histogram Mad | 1.23e-02 ± 3.37e-04 | 0.72 ± 0.71 | 4.34 ± 3.32 | 0.94 ± 0.06 | 0.40 ± 0.15 |
| Histogram Max | 9.88e-02 ± 1.76e-02 | 8.72 ± 5.80 | 36.79 ± 25.35 | 0.85 ± 0.06 | 0.27 ± 0.01 |
| Histogram Mean | 7.81e-03 ± 0.00e+00 | 0.00 ± 0.00 | 0.00 ± 0.00 | 1.00 ± 0.00 | 1.00 ± 0.00 |
| Histogram Median | 0.00e+00 ± 0.00e+00 | 0.00 ± 0.00 | 0.00 ± 0.00 | 1.00 ± 0.00 | 1.00 ± 0.00 |
| Histogram Min | 0.00e+00 ± 0.00e+00 | 0.00 ± 0.00 | 0.00 ± 0.00 | 1.00 ± 0.00 | 1.00 ± 0.00 |
| Histogram Range | 9.88e-02 ± 1.76e-02 | 8.72 ± 5.80 | 36.79 ± 25.35 | 0.85 ± 0.06 | 0.27 ± 0.01 |
| Histogram RMS | 2.10e-02 ± 1.77e-03 | 3.71 ± 3.03 | 16.30 ± 14.25 | 0.88 ± 0.10 | 0.35 ± 0.21 |
| Histogram Skewness | 2.91e+00 ± 4.47e-01 | 7.33 ± 6.13 | 26.49 ± 17.82 | 0.84 ± 0.07 | 0.29 ± 0.04 |
| Histogram STD | 1.95e-02 ± 1.91e-03 | 4.30 ± 3.49 | 18.71 ± 16.02 | 0.88 ± 0.10 | 0.35 ± 0.20 |
| Histogram Uniformity | 5.67e-02 ± 9.73e-03 | 7.58 ± 6.39 | 36.25 ± 37.45 | 0.87 ± 0.11 | 0.34 ± 0.25 |
| Histogram Variance | 3.85e-04 ± 7.66e-05 | 8.80 ± 7.41 | 42.05 ± 43.51 | 0.87 ± 0.11 | 0.34 ± 0.25 |
| Histogram TotalFrequency | 1.81e+03 ± 1.34e+03 | 18.47 ± 7.93 | 91.16 ± 15.58 | 0.94 ± 0.05 | 0.39 ± 0.24 |
| Signal Quantile 0.01 | 3.58e+02 ± 2.16e+02 | 10.07 ± 13.00 | 41.59 ± 31.31 | 0.97 ± 0.00 | 0.67 ± 0.33 |
| Signal Quantile 0.1 | 6.33e+02 ± 1.78e+02 | 1.97 ± 1.99 | 9.25 ± 11.06 | 1.00 ± 0.00 | 0.93 ± 0.04 |
| Signal Quantile 0.2 | 7.37e+02 ± 1.62e+02 | 1.70 ± 1.45 | 7.11 ± 7.05 | 1.00 ± 0.00 | 0.94 ± 0.00 |
| Signal Quantile 0.3 | 8.16e+02 ± 1.52e+02 | 1.81 ± 1.09 | 7.90 ± 5.30 | 0.99 ± 0.00 | 0.90 ± 0.01 |
| Signal Quantile 0.4 | 8.92e+02 ± 1.50e+02 | 2.27 ± 1.76 | 9.72 ± 7.91 | 0.99 ± 0.01 | 0.80 ± 0.08 |
| Signal Quantile 0.5 | 9.83e+02 ± 1.49e+02 | 3.45 ± 3.49 | 11.02 ± 8.82 | 0.95 ± 0.01 | 0.70 ± 0.03 |
| Signal Quantile 0.6 | 1.10e+03 ± 1.56e+02 | 3.84 ± 4.42 | 11.19 ± 8.60 | 0.91 ± 0.08 | 0.63 ± 0.22 |
| Signal Quantile 0.7 | 1.22e+03 ± 1.75e+02 | 3.45 ± 3.08 | 11.12 ± 9.20 | 0.93 ± 0.06 | 0.60 ± 0.38 |
| Signal Quantile 0.8 | 1.37e+03 ± 1.95e+02 | 2.65 ± 2.34 | 10.26 ± 10.35 | 0.96 ± 0.04 | 0.62 ± 0.44 |
| Signal Quantile 0.9 | 1.57e+03 ± 2.04e+02 | 2.10 ± 1.88 | 7.89 ± 9.88 | 0.98 ± 0.02 | 0.64 ± 0.43 |
| Signal Quantile 0.99 | 2.02e+03 ± 2.26e+02 | 2.29 ± 2.98 | 7.96 ± 8.66 | 0.96 ± 0.04 | 0.65 ± 0.34 |

**Table 45** Grey Level Co-occurrence Matrix (GLCM) features for the patients of the OroPharyngeal Cancers (OPC) dataset. In the table, the features computed for 16-bins histogram discretization are presented. The first column shows the mean and standard deviations of the original radiomic features computed on all the patients. The mean and standard deviation of both percentage difference and Intra-class Correlation Coefficient (ICC) are shown for both minimum and maximum entity scaling. Values are shown with scientific format only for the original features. The numbers are approximated to the second decimal significant digit. Features for which mean ICC_SMALL_ is less than 0.78 (unstable features) are highlighted in red.

| **GREY LEVEL CO-OCCURRENCE MATRIX (16-BINS DISCRETIZATION)** | | | | | |
| --- | --- | --- | --- | --- | --- |
| **Feature name** | **Original value** | **Diff %**  **(min. entity)** | **Diff %**  **(max. entity)** | **ICC_SMALL_** | **ICC_LARGE_** |
| Autocorrelation | 1.40e+01 ± 4.53e+00 | 5.57 ± 5.22 | 20.70 ± 14.04 | 0.97 ± 0.02 | 0.76 ± 0.06 |
| Cluster Prominence | 5.14e+03 ± 2.78e+03 | 10.72 ± 9.78 | 39.13 ± 24.90 | 0.95 ± 0.04 | 0.70 ± 0.08 |
| Cluster Shade | 5.14e+02 ± 2.20e+02 | 8.32 ± 7.59 | 30.47 ± 19.61 | 0.96 ± 0.03 | 0.71 ± 0.07 |
| Cluster Tendency | 5.68e+01 ± 1.75e+01 | 5.61 ± 5.17 | 20.69 ± 14.09 | 0.97 ± 0.03 | 0.74 ± 0.07 |
| Contrast | 2.63e+00 ± 8.39e-01 | 7.71 ± 4.89 | 32.15 ± 20.79 | 0.96 ± 0.02 | 0.59 ± 0.04 |
| Correlation | 1.27e+03 ± 5.16e+02 | 13.47 ± 11.16 | 53.17 ± 38.12 | 0.90 ± 0.07 | 0.45 ± 0.03 |
| Difference Entropy | 1.97e+00 ± 1.87e-01 | 2.67 ± 1.95 | 10.28 ± 7.84 | 0.94 ± 0.03 | 0.51 ± 0.05 |
| Dissimilarity | 1.16e+00 ± 2.15e-01 | 4.62 ± 2.99 | 18.89 ± 11.96 | 0.96 ± 0.03 | 0.59 ± 0.05 |
| Energy | 7.10e-02 ± 2.28e-02 | 12.89 ± 10.45 | 57.50 ± 40.34 | 0.88 ± 0.09 | 0.32 ± 0.03 |
| Entropy | 4.53e+00 ± 4.62e-01 | 3.80 ± 2.92 | 16.64 ± 10.15 | 0.91 ± 0.08 | 0.40 ± 0.06 |
| Homogeneity | 5.94e-01 ± 4.75e-02 | 1.99 ± 1.22 | 7.52 ± 5.13 | 0.96 ± 0.03 | 0.59 ± 0.11 |
| Homogeneity2 | 5.54e-01 ± 5.82e-02 | 2.62 ± 1.61 | 9.86 ± 6.72 | 0.96 ± 0.03 | 0.59 ± 0.11 |
| IMOC1 | -1.39e-01 ± 1.08e-01 | 21.59 ± 23.44 | 79.47 ± 83.62 | 0.70 ± 0.41 | 0.34 ± 0.48 |
| IMOC2 | 6.14e-01 ± 1.32e-01 | 4.32 ± 3.87 | 14.99 ± 16.54 | 0.97 ± 0.01 | 0.69 ± 0.21 |
| Inertia | 1.00e+00 ± 1.28e-05 | 0.00 ± 0.00 | 0.00 ± 0.00 | 0.96 ± 0.02 | 0.59 ± 0.04 |
| Inverse Difference moment | 9.96e-01 ± 8.27e-04 | 0.02 ± 0.01 | 0.08 ± 0.05 | 0.96 ± 0.03 | 0.59 ± 0.05 |
| Inverse Difference moment2 | 4.55e-01 ± 3.79e-02 | 2.04 ± 1.89 | 6.72 ± 6.57 | 0.95 ± 0.01 | 0.54 ± 0.03 |
| Inverse Variance | 1.66e-01 ± 4.91e-02 | 8.72 ± 6.09 | 38.80 ± 21.90 | 0.93 ± 0.04 | 0.39 ± 0.14 |
| Max Probability | 7.16e+00 ± 1.21e+00 | 2.69 ± 2.50 | 10.30 ± 6.98 | 0.98 ± 0.02 | 0.78 ± 0.08 |
| Sum Average | 3.11e+00 ± 2.60e-01 | 2.88 ± 2.73 | 13.34 ± 10.42 | 0.91 ± 0.08 | 0.42 ± 0.14 |
| Sum Entropy | 2.63e+00 ± 8.39e-01 | 7.71 ± 4.89 | 32.15 ± 20.79 | 0.96 ± 0.02 | 0.59 ± 0.04 |

**Table 46** Grey Level Co-occurrence Matrix (GLCM) features for the patients of the OroPharyngeal Cancers (OPC) dataset. In the table, the features computed for 32-bins histogram discretization are presented. The first column shows the mean and standard deviations of the original radiomic features computed on all the patients. The mean and standard deviation of both percentage difference and Intra-class Correlation Coefficient (ICC) are shown for both minimum and maximum entity scaling. Values are shown with scientific format only for the original features. The numbers are approximated to the second decimal significant digit.

| **GREY LEVEL CO-OCCURRENCE MATRIX (32-BINS DISCRETIZATION)** | | | | | |
| --- | --- | --- | --- | --- | --- |
| **Feature name** | **Original value** | **Diff %**  **(min. entity)** | **Diff %**  **(max. entity)** | **ICC_SMALL_** | **ICC_LARGE_** |
| Autocorrelation | 6.32e+01 ± 1.93e+01 | 5.26 ± 4.91 | 19.52 ± 13.01 | 0.97 ± 0.02 | 0.76 ± 0.06 |
| Cluster Prominence | 1.03e+05 ± 5.31e+04 | 10.23 ± 9.31 | 37.29 ± 23.24 | 0.95 ± 0.04 | 0.70 ± 0.09 |
| Cluster Shade | 4.95e+03 ± 2.02e+03 | 7.86 ± 7.19 | 28.81 ± 18.32 | 0.96 ± 0.03 | 0.72 ± 0.07 |
| Cluster Tendency | 2.61e+02 ± 7.56e+01 | 5.25 ± 4.84 | 19.38 ± 12.97 | 0.97 ± 0.03 | 0.74 ± 0.07 |
| Contrast | 1.01e+01 ± 3.44e+00 | 7.98 ± 5.34 | 33.01 ± 20.17 | 0.97 ± 0.02 | 0.62 ± 0.03 |
| Correlation | 2.23e+04 ± 9.16e+03 | 14.51 ± 11.26 | 57.53 ± 40.60 | 0.90 ± 0.07 | 0.43 ± 0.05 |
| Difference Entropy | 2.77e+00 ± 2.26e-01 | 2.28 ± 1.68 | 9.22 ± 6.84 | 0.95 ± 0.04 | 0.50 ± 0.06 |
| Dissimilarity | 2.33e+00 ± 4.43e-01 | 4.86 ± 3.06 | 18.15 ± 11.07 | 0.96 ± 0.02 | 0.63 ± 0.01 |
| Energy | 2.32e-02 ± 1.04e-02 | 15.62 ± 11.66 | 81.93 ± 66.44 | 0.90 ± 0.05 | 0.30 ± 0.04 |
| Entropy | 6.18e+00 ± 6.42e-01 | 3.45 ± 2.49 | 16.23 ± 10.17 | 0.93 ± 0.06 | 0.47 ± 0.10 |
| Homogeneity | 4.43e-01 ± 4.81e-02 | 2.92 ± 1.69 | 8.56 ± 6.31 | 0.96 ± 0.03 | 0.63 ± 0.00 |
| Homogeneity2 | 3.71e-01 ± 5.89e-02 | 4.29 ± 2.54 | 12.45 ± 9.12 | 0.96 ± 0.03 | 0.63 ± 0.02 |
| IMOC1 | -1.58e-01 ± 1.27e-01 | 18.71 ± 20.92 | 70.35 ± 70.15 | 0.80 ± 0.27 | 0.29 ± 0.42 |
| IMOC2 | 7.38e-01 ± 1.31e-01 | 2.70 ± 2.77 | 10.77 ± 10.45 | 0.98 ± 0.01 | 0.77 ± 0.16 |
| Inertia | 1.00e+00 ± 3.35e-06 | 0.00 ± 0.00 | 0.00 ± 0.00 | 0.95 ± 0.03 | 0.63 ± 0.04 |
| Inverse Difference moment | 9.98e-01 ± 4.29e-04 | 0.01 ± 0.01 | 0.04 ± 0.02 | 0.96 ± 0.02 | 0.63 ± 0.01 |
| Inverse Difference moment2 | 3.59e-01 ± 4.71e-02 | 3.41 ± 2.36 | 13.75 ± 10.33 | 0.95 ± 0.03 | 0.59 ± 0.01 |
| Inverse Variance | 6.46e-02 ± 2.83e-02 | 11.67 ± 8.69 | 58.07 ± 49.24 | 0.92 ± 0.01 | 0.36 ± 0.08 |
| Max Probability | 1.53e+01 ± 2.41e+00 | 2.52 ± 2.36 | 9.65 ± 6.39 | 0.98 ± 0.02 | 0.78 ± 0.08 |
| Sum Average | 4.06e+00 ± 2.86e-01 | 2.53 ± 2.36 | 12.58 ± 11.71 | 0.91 ± 0.08 | 0.41 ± 0.16 |
| Sum Entropy | 1.01e+01 ± 3.44e+00 | 7.98 ± 5.34 | 33.01 ± 20.17 | 0.97 ± 0.02 | 0.62 ± 0.03 |

**Table 47** Grey Level Co-occurrence Matrix (GLCM) features for the patients of the OroPharyngeal Cancers (OPC) dataset. In the table, the features computed for 64-bins histogram discretization are presented. The first column shows the mean and standard deviations of the original radiomic features computed on all the patients. The mean and standard deviation of both percentage difference and Intra-class Correlation Coefficient (ICC) are shown for both minimum and maximum entity scaling. Values are shown with scientific format only for the original features. The numbers are approximated to the second decimal significant digit.

| **GREY LEVEL CO-OCCURRENCE MATRIX (64-BINS DISCRETIZATION)** | | | | | |
| --- | --- | --- | --- | --- | --- |
| **Feature name** | **Original value** | **Diff %**  **(min. entity)** | **Diff %**  **(max. entity)** | **ICC_SMALL_** | **ICC_LARGE_** |
| Autocorrelation | 2.68e+02 ± 7.98e+01 | 5.06 ± 4.74 | 18.79 ± 12.60 | 0.97 ± 0.02 | 0.76 ± 0.06 |
| Cluster Prominence | 1.82e+06 ± 9.20e+05 | 9.91 ± 9.07 | 36.27 ± 22.74 | 0.95 ± 0.04 | 0.70 ± 0.09 |
| Cluster Shade | 4.31e+04 ± 1.71e+04 | 7.59 ± 6.97 | 27.86 ± 17.88 | 0.96 ± 0.03 | 0.72 ± 0.07 |
| Cluster Tendency | 1.11e+03 ± 3.13e+02 | 5.05 ± 4.67 | 18.64 ± 12.57 | 0.97 ± 0.03 | 0.74 ± 0.07 |
| Contrast | 4.01e+01 ± 1.39e+01 | 7.99 ± 5.18 | 33.91 ± 20.66 | 0.97 ± 0.02 | 0.62 ± 0.03 |
| Correlation | 3.70e+05 ± 1.54e+05 | 14.74 ± 11.39 | 59.84 ± 41.22 | 0.91 ± 0.06 | 0.42 ± 0.03 |
| Difference Entropy | 3.65e+00 ± 2.54e-01 | 2.06 ± 1.71 | 8.70 ± 5.94 | 0.94 ± 0.05 | 0.49 ± 0.06 |
| Dissimilarity | 4.66e+00 ± 9.05e-01 | 4.81 ± 2.98 | 18.27 ± 10.82 | 0.96 ± 0.02 | 0.64 ± 0.00 |
| Energy | 8.55e-03 ± 6.12e-03 | 18.18 ± 12.52 | 116.23 ± 105.67 | 0.94 ± 0.06 | 0.39 ± 0.21 |
| Entropy | 7.62e+00 ± 9.68e-01 | 3.26 ± 2.33 | 16.55 ± 10.05 | 0.96 ± 0.04 | 0.59 ± 0.17 |
| Homogeneity | 3.10e-01 ± 4.29e-02 | 3.65 ± 2.28 | 10.36 ± 7.71 | 0.96 ± 0.02 | 0.64 ± 0.07 |
| Homogeneity2 | 2.22e-01 ± 4.75e-02 | 5.66 ± 4.15 | 15.68 ± 10.85 | 0.96 ± 0.02 | 0.65 ± 0.10 |
| IMOC1 | -1.64e-01 ± 1.10e-01 | 16.22 ± 17.53 | 81.74 ± 100.56 | 0.82 ± 0.21 | 0.18 ± 0.25 |
| IMOC2 | 8.66e-01 ± 9.76e-02 | 1.60 ± 1.73 | 7.03 ± 7.01 | 0.98 ± 0.02 | 0.71 ± 0.25 |
| Inertia | 1.00e+00 ± 8.26e-07 | 0.00 ± 0.00 | 0.00 ± 0.00 | 0.88 ± 0.11 | 0.62 ± 0.06 |
| Inverse Difference moment | 9.99e-01 ± 2.20e-04 | 0.01 ± 0.00 | 0.02 ± 0.01 | 0.96 ± 0.02 | 0.64 ± 0.00 |
| Inverse Difference moment2 | 2.30e-01 ± 4.85e-02 | 5.21 ± 4.27 | 15.32 ± 9.82 | 0.95 ± 0.05 | 0.65 ± 0.07 |
| Inverse Variance | 2.54e-02 ± 1.57e-02 | 15.41 ± 9.00 | 74.12 ± 38.24 | 0.94 ± 0.00 | 0.44 ± 0.06 |
| Max Probability | 3.16e+01 ± 4.83e+00 | 2.43 ± 2.27 | 9.28 ± 6.14 | 0.98 ± 0.02 | 0.78 ± 0.08 |
| Sum Average | 4.99e+00 ± 3.44e-01 | 2.32 ± 2.22 | 11.53 ± 9.97 | 0.92 ± 0.08 | 0.45 ± 0.15 |
| Sum Entropy | 4.01e+01 ± 1.39e+01 | 7.99 ± 5.18 | 33.91 ± 20.66 | 0.97 ± 0.02 | 0.62 ± 0.03 |

**Table 48** Grey Level Run Length Matrix (GLRLM) features for the patients of the OroPharyngeal Cancers (OPC) dataset. In the table, the features computed for 16-bins histogram discretization are presented. The first column shows the mean and standard deviations of the original radiomic features computed on all the patients. The mean and standard deviation of both percentage difference and Intra-class Correlation Coefficient (ICC) are shown for both minimal and maximal entity scaling. Values are shown with scientific format only for the original features. The numbers are approximated to the second decimal significant digit.

| **GREY LEVEL RUN LENGTH MATRIX (16-BINS DISCRETIZATION)** | | | | | |
| --- | --- | --- | --- | --- | --- |
| **Feature name** | **Original value** | **Diff %**  **(min. entity)** | **Diff %**  **(max. entity)** | **ICC_SMALL_** | **ICC_LARGE_** |
| Short Run Emphasis | 9.78e-01 ± 1.43e-02 | 0.29 ± 0.22 | 1.16 ± 1.35 | 0.97 ± 0.01 | 0.70 ± 0.15 |
| Long Run Emphasis | 1.10e+00 ± 7.03e-02 | 1.10 ± 0.80 | 4.45 ± 4.92 | 0.98 ± 0.01 | 0.72 ± 0.13 |
| Grey Level Non-Uniformity | 2.55e+02 ± 1.86e+02 | 15.18 ± 7.16 | 79.60 ± 17.10 | 0.96 ± 0.03 | 0.46 ± 0.20 |
| Run Length Non-Uniformity | 1.31e+03 ± 9.53e+02 | 19.39 ± 7.92 | 96.67 ± 21.84 | 0.93 ± 0.06 | 0.36 ± 0.22 |
| Run Percentage | 7.91e-01 ± 5.71e-02 | 1.25 ± 1.07 | 4.21 ± 3.62 | 0.98 ± 0.02 | 0.79 ± 0.07 |
| Low Grey Level Run Emphasis | 7.91e-02 ± 5.02e-02 | 4.59 ± 3.14 | 21.32 ± 17.76 | 1.00 ± 0.00 | 0.89 ± 0.02 |
| high Grey Level Run Emphasis | 2.62e+01 ± 6.58e+00 | 3.85 ± 2.86 | 14.35 ± 11.59 | 0.98 ± 0.02 | 0.73 ± 0.27 |
| Short Run Low Grey Level Emphasis | 7.77e-02 ± 5.06e-02 | 4.63 ± 3.21 | 21.04 ± 17.85 | 1.00 ± 0.00 | 0.90 ± 0.01 |
| Short Run High Grey Level Emphasis | 2.58e+01 ± 6.37e+00 | 3.96 ± 2.95 | 14.66 ± 12.09 | 0.97 ± 0.02 | 0.71 ± 0.30 |
| Long Run Low Grey Level Emphasis | 8.52e-02 ± 4.86e-02 | 4.50 ± 3.36 | 22.10 ± 19.85 | 0.99 ± 0.00 | 0.85 ± 0.08 |
| Long Run High Grey Level Emphasis | 2.82e+01 ± 7.77e+00 | 3.45 ± 2.62 | 13.26 ± 10.06 | 0.98 ± 0.01 | 0.82 ± 0.16 |

**Table 49** Grey Level Run Length Matrix (GLRLM) features for the patients of the OroPharyngeal Cancers (OPC) dataset. In the table, the features computed for 32-bins histogram discretization are presented. The first column shows the mean and standard deviations of the original radiomic features computed on all the patients. The mean and standard deviation of both percentage difference and Intra-class Correlation Coefficient (ICC) are shown for both minimal and maximal entity scaling. Values are shown with scientific format only for the original features. The numbers are approximated to the second decimal significant digit.

| **GREY LEVEL RUN LENGTH MATRIX (32-BINS DISCRETIZATION)** | | | | | |
| --- | --- | --- | --- | --- | --- |
| **Feature name** | **Original value** | **Diff %**  **(min. entity)** | **Diff %**  **(max. entity)** | **ICC_SMALL_** | **ICC_LARGE_** |
| Short Run Emphasis | 9.79e-01 ± 1.10e-02 | 0.22 ± 0.20 | 0.88 ± 0.95 | 0.97 ± 0.02 | 0.71 ± 0.13 |
| Long Run Emphasis | 1.11e+00 ± 6.23e-02 | 1.08 ± 0.95 | 4.11 ± 4.96 | 0.97 ± 0.02 | 0.70 ± 0.13 |
| Grey Level Non-Uniformity | 1.59e+02 ± 1.18e+02 | 13.57 ± 7.16 | 73.92 ± 14.61 | 0.97 ± 0.02 | 0.51 ± 0.20 |
| Run Length Non-Uniformity | 1.49e+03 ± 1.08e+03 | 19.12 ± 8.05 | 94.64 ± 19.40 | 0.93 ± 0.05 | 0.37 ± 0.22 |
| Run Percentage | 8.88e-01 ± 3.43e-02 | 0.81 ± 0.52 | 2.19 ± 1.67 | 0.97 ± 0.01 | 0.78 ± 0.01 |
| Low Grey Level Run Emphasis | 2.89e-02 ± 2.63e-02 | 6.34 ± 4.80 | 29.09 ± 28.21 | 0.99 ± 0.00 | 0.89 ± 0.01 |
| High Grey Level Run Emphasis | 9.26e+01 ± 2.36e+01 | 4.10 ± 3.20 | 15.17 ± 11.73 | 0.97 ± 0.02 | 0.74 ± 0.25 |
| Short Run Low Grey Level Emphasis | 2.84e-02 ± 2.61e-02 | 6.54 ± 4.98 | 29.33 ± 28.98 | 0.99 ± 0.01 | 0.89 ± 0.00 |
| Short Run High Grey Level Emphasis | 9.12e+01 ± 2.29e+01 | 4.21 ± 3.29 | 15.45 ± 12.17 | 0.97 ± 0.02 | 0.72 ± 0.26 |
| Long Run Low Grey Level Emphasis | 3.16e-02 ± 2.72e-02 | 6.25 ± 4.52 | 29.14 ± 26.11 | 0.99 ± 0.00 | 0.88 ± 0.03 |
| Long Run High Grey Level Emphasis | 9.98e+01 ± 2.75e+01 | 3.58 ± 2.71 | 13.64 ± 9.82 | 0.98 ± 0.01 | 0.82 ± 0.16 |

**Table 50** Grey Level Run Length Matrix (GLRLM) features for the patients of the OroPharyngeal Cancers (OPC) dataset. In the table, the features computed for 64-bins histogram discretization are presented. The first column shows the mean and standard deviations of the original radiomic features computed on all the patients. The mean and standard deviation of both percentage difference and Intra-class Correlation Coefficient (ICC) are shown for both minimal and maximal entity scaling. Values are shown with scientific format only for the original features. The numbers are approximated to the second decimal significant digit.

| **GREY LEVEL RUN LENGTH MATRIX (64-BINS DISCRETIZATION)** | | | | | |
| --- | --- | --- | --- | --- | --- |
| **Feature name** | **Original value** | **Diff %**  **(min. entity)** | **Diff %**  **(max. entity)** | **ICC_SMALL_** | **ICC_LARGE_** |
| Short Run Emphasis | 9.41e-01 ± 1.25e-02 | 0.48 ± 0.41 | 1.47 ± 1.54 | 0.91 ± 0.08 | 0.52 ± 0.14 |
| Long Run Emphasis | 1.30e+00 ± 8.36e-02 | 1.98 ± 1.38 | 6.97 ± 5.97 | 0.93 ± 0.05 | 0.56 ± 0.17 |
| Grey Level Non-Uniformity | 8.94e+01 ± 6.71e+01 | 12.75 ± 7.23 | 70.26 ± 14.31 | 0.98 ± 0.02 | 0.54 ± 0.21 |
| Run Length Non-Uniformity | 1.43e+03 ± 1.05e+03 | 19.26 ± 8.23 | 94.99 ± 19.51 | 0.93 ± 0.05 | 0.37 ± 0.22 |
| Run Percentage | 9.42e-01 ± 1.96e-02 | 0.49 ± 0.35 | 1.19 ± 0.86 | 0.96 ± 0.00 | 0.78 ± 0.06 |
| Low Grey Level Run Emphasis | 1.08e-02 ± 1.35e-02 | 8.14 ± 7.48 | 46.67 ± 52.55 | 0.99 ± 0.01 | 0.87 ± 0.00 |
| high Grey Level Run Emphasis | 3.47e+02 ± 8.93e+01 | 4.26 ± 3.34 | 15.63 ± 11.81 | 0.97 ± 0.02 | 0.73 ± 0.23 |
| Short Run Low Grey Level Emphasis | 1.02e-02 ± 1.30e-02 | 8.21 ± 8.13 | 48.23 ± 54.75 | 0.99 ± 0.01 | 0.86 ± 0.01 |
| Short Run High Grey Level Emphasis | 3.30e+02 ± 8.35e+01 | 4.48 ± 3.51 | 16.26 ± 12.26 | 0.97 ± 0.02 | 0.71 ± 0.25 |
| Long Run Low Grey Level Emphasis | 1.34e-02 ± 1.57e-02 | 7.91 ± 5.87 | 45.00 ± 43.74 | 1.00 ± 0.00 | 0.89 ± 0.01 |
| Long Run High Grey Level Emphasis | 4.29e+02 ± 1.24e+02 | 3.64 ± 2.77 | 13.22 ± 9.42 | 0.98 ± 0.01 | 0.83 ± 0.12 |

- 1. **Soft-Tissues Sarcomas dataset**

**Table 51** First-Order Statistics (FOS) features for the patients of the Soft-Tissues Sarcomas (STS) dataset. In the table, the bin-independent features are presented. The first column shows the mean and standard deviations of the original radiomic features computed on all the patients. The mean and standard deviation of both percentage difference and Intra-class Correlation Coefficient (ICC) are shown for both minimum and maximum entity scaling. Values are shown with scientific format only for the original features. The numbers are approximated to the second decimal significant digit. Features for which mean ICC_SMALL_ is less than 0.78 (unstable features) are highlighted in red.

| **FIRST-ORDER STATISTICS (BIN-INDEPENDENT)** | | | | | |
| --- | --- | --- | --- | --- | --- |
| **Feature name** | **Original value** | **Diff %**  **(min. entity)** | **Diff %**  **(max. entity)** | **ICC_SMALL_** | **ICC_LARGE_** |
| Signal Energy | 1.34e+11 ± 1.11e+11 | 14.24 ± 9.38 | 54.28 ± 24.68 | 0.97 ± 0.03 | 0.59 ± 0.43 |
| Signal Kurtosis | 3.17e+00 ± 1.05e+00 | 6.74 ± 7.76 | 19.99 ± 16.65 | 0.93 ± 0.07 | 0.64 ± 0.17 |
| Signal Mad | 4.37e+02 ± 1.26e+02 | 4.24 ± 3.64 | 19.38 ± 21.92 | 0.99 ± 0.00 | 0.69 ± 0.26 |
| Signal Max | 3.41e+03 ± 3.80e+02 | 0.86 ± 1.83 | 4.69 ± 6.41 | 0.98 ± 0.01 | 0.84 ± 0.15 |
| Signal Mean | 1.48e+03 ± 3.20e+02 | 2.95 ± 1.78 | 15.49 ± 9.10 | 0.98 ± 0.00 | 0.71 ± 0.28 |
| Signal Median | 1.46e+03 ± 4.06e+02 | 3.28 ± 3.40 | 16.16 ± 10.62 | 0.98 ± 0.00 | 0.68 ± 0.33 |
| Signal Min | 6.93e+00 ± 1.80e+01 | 8.4e+11 ± 5.04e+12 | 2.34e+13 ± 1.4e+14 | 0.28 ± 0.28 | 0.05 ± 0.07 |
| Signal Range | 3.40e+03 ± 3.87e+02 | 1.22 ± 2.27 | 5.77 ± 7.71 | 0.98 ± 0.02 | 0.80 ± 0.20 |
| Signal RMS | 1.58e+03 ± 3.30e+02 | 2.51 ± 1.46 | 12.17 ± 6.60 | 0.99 ± 0.00 | 0.79 ± 0.18 |
| Signal Skewness | 2.47e-01 ± 6.00e-01 | 493.07 ± 1502.92 | 1062.88 ± 3167.72 | 0.97 ± 0.01 | 0.78 ± 0.10 |
| Signal STD | 5.35e+02 ± 1.26e+02 | 4.20 ± 3.56 | 17.14 ± 18.41 | 0.98 ± 0.01 | 0.70 ± 0.27 |
| Signal Variance | 3.01e+05 ± 1.50e+05 | 8.43 ± 7.13 | 38.82 ± 48.41 | 0.98 ± 0.01 | 0.73 ± 0.25 |

**Table 52** First-Order Statistics (FOS) features for the patients of the Soft-Tissues Sarcomas (STS) dataset. In the table, the bin-dependent features calculated with a 16-bins discretization are presented. The first column shows the mean and standard deviations of the original radiomic features computed on all the patients. The mean and standard deviation of both percentage difference and Intra-class Correlation Coefficient (ICC) are shown for both minimum and maximum entity scaling. Values are shown with scientific format only for the original features. The numbers are approximated to the second decimal significant digit. Features for which mean ICC_SMALL_ is less than 0.7 (unstable features) are highlighted in red.

| **FIRST-ORDER STATISTICS (16-BINS DISCRETIZATION)** | | | | | |
| --- | --- | --- | --- | --- | --- |
| **Feature name** | **Original value** | **Diff %**  **(min. entity)** | **Diff %**  **(max. entity)** | **ICC_SMALL_** | **ICC_LARGE_** |
| Histogram Entropy | 2.99e+00 ± 2.61e-01 | 2.44 ± 1.94 | 6.25 ± 6.68 | 0.95 ± 0.01 | 0.66 ± 0.16 |
| Histogram Kurtosis | 6.74e+00 ± 3.81e+00 | 8.29 ± 8.75 | 29.94 ± 18.81 | 0.98 ± 0.01 | 0.57 ± 0.48 |
| Histogram Mad | 4.39e-02 ± 2.56e-03 | 1.77 ± 1.32 | 3.98 ± 3.67 | 0.93 ± 0.01 | 0.68 ± 0.20 |
| Histogram Max | 2.34e-01 ± 6.81e-02 | 5.29 ± 5.54 | 19.31 ± 12.07 | 0.96 ± 0.02 | 0.62 ± 0.27 |
| Histogram Mean | 3.13e-02 ± 0.00e+00 | 0.00 ± 0.00 | 0.00 ± 0.00 | 1.00 ± 0.00 | 1.00 ± 0.00 |
| Histogram Median | 2.07e-05 ± 6.82e-05 | 0.00 ± 0.00 | 0.00 ± 0.00 | 0.98 ± 0.02 | 0.53 ± 0.45 |
| Histogram Min | 0.00e+00 ± 0.00e+00 | 0.00 ± 0.00 | 0.00 ± 0.00 | 1.00 ± 0.00 | 1.00 ± 0.00 |
| Histogram Range | 2.34e-01 ± 6.81e-02 | 5.29 ± 5.54 | 19.31 ± 12.07 | 0.96 ± 0.02 | 0.62 ± 0.27 |
| Histogram RMS | 6.89e-02 ± 7.77e-03 | 2.67 ± 2.44 | 7.67 ± 7.43 | 0.95 ± 0.02 | 0.62 ± 0.22 |
| Histogram Skewness | 2.10e+00 ± 6.69e-01 | 5.24 ± 5.03 | 19.51 ± 13.28 | 0.98 ± 0.01 | 0.60 ± 0.43 |
| Histogram STD | 6.23e-02 ± 8.91e-03 | 3.32 ± 2.91 | 9.60 ± 9.07 | 0.95 ± 0.01 | 0.63 ± 0.23 |
| Histogram Uniformity | 1.54e-01 ± 3.44e-02 | 5.37 ± 5.05 | 15.04 ± 14.94 | 0.94 ± 0.03 | 0.59 ± 0.17 |
| Histogram Variance | 3.96e-03 ± 1.11e-03 | 6.68 ± 6.06 | 18.64 ± 18.00 | 0.94 ± 0.03 | 0.59 ± 0.17 |
| Histogram TotalFrequency | 4.90e+04 ± 3.69e+04 | 18.07 ± 7.40 | 83.35 ± 11.32 | 0.95 ± 0.04 | 0.43 ± 0.27 |
| Signal Quantile 0.01 | 3.39e+02 ± 1.51e+02 | 39.18 ± 25.05 | 77.10 ± 45.57 | 0.68 ± 0.06 | 0.27 ± 0.32 |
| Signal Quantile 0.1 | 8.17e+02 ± 2.06e+02 | 7.30 ± 6.59 | 37.71 ± 29.24 | 0.93 ± 0.06 | 0.46 ± 0.55 |
| Signal Quantile 0.2 | 1.00e+03 ± 2.41e+02 | 4.04 ± 3.24 | 28.42 ± 22.23 | 0.97 ± 0.02 | 0.50 ± 0.48 |
| Signal Quantile 0.3 | 1.15e+03 ± 2.81e+02 | 3.64 ± 3.12 | 23.16 ± 17.18 | 0.97 ± 0.00 | 0.55 ± 0.36 |
| Signal Quantile 0.4 | 1.30e+03 ± 3.37e+02 | 3.74 ± 3.84 | 19.08 ± 12.60 | 0.96 ± 0.00 | 0.63 ± 0.34 |
| Signal Quantile 0.5 | 1.46e+03 ± 4.02e+02 | 3.22 ± 3.39 | 16.28 ± 10.67 | 0.98 ± 0.00 | 0.68 ± 0.33 |
| Signal Quantile 0.6 | 1.61e+03 ± 4.32e+02 | 2.86 ± 2.63 | 14.17 ± 9.43 | 0.98 ± 0.00 | 0.72 ± 0.28 |
| Signal Quantile 0.7 | 1.77e+03 ± 4.52e+02 | 2.19 ± 1.63 | 12.16 ± 9.63 | 0.99 ± 0.00 | 0.77 ± 0.17 |
| Signal Quantile 0.8 | 1.95e+03 ± 4.41e+02 | 1.96 ± 1.63 | 10.01 ± 7.66 | 0.99 ± 0.00 | 0.85 ± 0.05 |
| Signal Quantile 0.9 | 2.20e+03 ± 4.12e+02 | 1.94 ± 1.95 | 7.38 ± 4.95 | 0.99 ± 0.00 | 0.91 ± 0.04 |
| Signal Quantile 0.99 | 2.74e+03 ± 3.34e+02 | 1.24 ± 1.74 | 5.20 ± 6.33 | 0.99 ± 0.01 | 0.86 ± 0.01 |

**Table 53** First-Order Statistics (FOS) features for the patients of the Soft-Tissues Sarcomas (STS) dataset. In the table, the bin-dependent features calculated with a 32-bins discretization are presented. The first column shows the mean and standard deviations of the original radiomic features computed on all the patients. The mean and standard deviation of both percentage difference and Intra-class Correlation Coefficient (ICC) are shown for both minimum and maximum entity scaling. Values are shown with scientific format only for the original features. The numbers are approximated to the second decimal significant digit. Features for which mean ICC_SMALL_ is less than 0.7 (unstable features) are highlighted in red.

| **FIRST-ORDER STATISTICS (32-BINS DISCRETIZATION)** | | | | | |
| --- | --- | --- | --- | --- | --- |
| **Feature name** | **Original value** | **Diff %**  **(min. entity)** | **Diff %**  **(max. entity)** | **ICC_SMALL_** | **ICC_LARGE_** |
| Histogram Entropy | 3.97e+00 ± 2.64e-01 | 1.92 ± 1.48 | 4.85 ± 5.08 | 0.95 ± 0.01 | 0.65 ± 0.17 |
| Histogram Kurtosis | 7.13e+00 ± 4.09e+00 | 8.78 ± 10.77 | 30.10 ± 19.23 | 0.98 ± 0.01 | 0.60 ± 0.44 |
| Histogram Mad | 2.21e-02 ± 1.30e-03 | 1.88 ± 1.24 | 4.04 ± 3.81 | 0.93 ± 0.01 | 0.67 ± 0.19 |
| Histogram Max | 1.26e-01 ± 4.04e-02 | 6.15 ± 5.89 | 19.60 ± 12.53 | 0.96 ± 0.02 | 0.67 ± 0.21 |
| Histogram Mean | 1.56e-02 ± 0.00e+00 | 0.00 ± 0.00 | 0.00 ± 0.00 | 1.00 ± 0.00 | 1.00 ± 0.00 |
| Histogram Median | 6.08e-06 ± 2.23e-05 | 0.00 ± 0.00 | 0.00 ± 0.00 | 0.98 ± 0.02 | 0.71 ± 0.38 |
| Histogram Min | 0.00e+00 ± 0.00e+00 | 0.00 ± 0.00 | 0.00 ± 0.00 | 1.00 ± 0.00 | 1.00 ± 0.00 |
| Histogram Range | 1.26e-01 ± 4.04e-02 | 6.15 ± 5.89 | 19.60 ± 12.53 | 0.96 ± 0.02 | 0.67 ± 0.21 |
| Histogram RMS | 3.49e-02 ± 4.06e-03 | 2.80 ± 2.51 | 8.02 ± 7.46 | 0.95 ± 0.02 | 0.63 ± 0.22 |
| Histogram Skewness | 2.16e+00 ± 6.99e-01 | 5.42 ± 5.63 | 19.56 ± 13.29 | 0.98 ± 0.01 | 0.62 ± 0.40 |
| Histogram STD | 3.14e-02 ± 4.59e-03 | 3.47 ± 2.98 | 9.98 ± 9.04 | 0.95 ± 0.02 | 0.63 ± 0.22 |
| Histogram Uniformity | 7.89e-02 ± 1.83e-02 | 5.65 ± 5.21 | 15.70 ± 14.95 | 0.94 ± 0.03 | 0.60 ± 0.16 |
| Histogram Variance | 1.00e-03 ± 2.90e-04 | 6.99 ± 6.21 | 19.35 ± 17.87 | 0.94 ± 0.03 | 0.60 ± 0.16 |
| Histogram TotalFrequency | 4.90e+04 ± 3.69e+04 | 18.07 ± 7.40 | 83.35 ± 11.32 | 0.95 ± 0.04 | 0.43 ± 0.27 |
| Signal Quantile 0.01 | 3.59e+02 ± 1.62e+02 | 38.51 ± 25.18 | 76.70 ± 48.81 | 0.67 ± 0.06 | 0.28 ± 0.32 |
| Signal Quantile 0.1 | 8.33e+02 ± 1.97e+02 | 6.74 ± 6.29 | 37.30 ± 29.95 | 0.93 ± 0.06 | 0.47 ± 0.57 |
| Signal Quantile 0.2 | 1.01e+03 ± 2.40e+02 | 3.89 ± 3.31 | 28.43 ± 22.59 | 0.97 ± 0.02 | 0.50 ± 0.49 |
| Signal Quantile 0.3 | 1.15e+03 ± 2.82e+02 | 3.55 ± 3.08 | 22.94 ± 17.35 | 0.97 ± 0.00 | 0.54 ± 0.36 |
| Signal Quantile 0.4 | 1.30e+03 ± 3.38e+02 | 3.76 ± 3.93 | 19.11 ± 12.58 | 0.96 ± 0.00 | 0.63 ± 0.34 |
| Signal Quantile 0.5 | 1.46e+03 ± 4.04e+02 | 3.25 ± 3.40 | 16.19 ± 10.60 | 0.98 ± 0.00 | 0.68 ± 0.33 |
| Signal Quantile 0.6 | 1.61e+03 ± 4.37e+02 | 2.87 ± 2.66 | 14.20 ± 9.48 | 0.98 ± 0.00 | 0.72 ± 0.28 |
| Signal Quantile 0.7 | 1.77e+03 ± 4.58e+02 | 2.27 ± 1.69 | 12.37 ± 9.81 | 0.99 ± 0.00 | 0.77 ± 0.17 |
| Signal Quantile 0.8 | 1.94e+03 ± 4.46e+02 | 1.94 ± 1.64 | 9.83 ± 7.89 | 0.99 ± 0.00 | 0.85 ± 0.05 |
| Signal Quantile 0.9 | 2.19e+03 ± 4.11e+02 | 1.90 ± 1.88 | 7.46 ± 5.09 | 0.99 ± 0.00 | 0.91 ± 0.04 |
| Signal Quantile 0.99 | 2.71e+03 ± 3.26e+02 | 1.29 ± 1.78 | 5.11 ± 6.27 | 0.99 ± 0.01 | 0.87 ± 0.00 |

**Table 54** First-Order Statistics (FOS) features for the patients of the Soft-Tissues Sarcomas (STS) dataset. In the table, the bin-dependent features calculated with a 64-bins discretization are presented. The first column shows the mean and standard deviations of the original radiomic features computed on all the patients. The mean and standard deviation of both percentage difference and Intra-class Correlation Coefficient (ICC) are shown for both minimum and maximum entity scaling. Values are shown with scientific format only for the original features. The numbers are approximated to the second decimal significant digit. Features for which mean ICC_SMALL_ is less than 0.7 (unstable features) are highlighted in red.

| **FIRST-ORDER STATISTICS (64-BINS DISCRETIZATION)** | | | | | |
| --- | --- | --- | --- | --- | --- |
| **Feature name** | **Original value** | **Diff %**  **(min. entity)** | **Diff %**  **(max. entity)** | **ICC_SMALL_** | **ICC_LARGE_** |
| Histogram Entropy | 4.96e+00 ± 2.65e-01 | 1.55 ± 1.18 | 3.93 ± 4.05 | 0.95 ± 0.01 | 0.65 ± 0.17 |
| Histogram Kurtosis | 7.30e+00 ± 4.33e+00 | 8.75 ± 10.98 | 30.52 ± 19.69 | 0.98 ± 0.01 | 0.60 ± 0.43 |
| Histogram Mad | 1.10e-02 ± 6.48e-04 | 1.86 ± 1.25 | 4.06 ± 3.83 | 0.93 ± 0.01 | 0.67 ± 0.19 |
| Histogram Max | 6.49e-02 ± 2.11e-02 | 6.09 ± 5.90 | 21.16 ± 12.90 | 0.96 ± 0.02 | 0.65 ± 0.19 |
| Histogram Mean | 7.81e-03 ± 0.00e+00 | 0.00 ± 0.00 | 0.00 ± 0.00 | 1.00 ± 0.00 | 1.00 ± 0.00 |
| Histogram Median | 2.60e-06 ± 9.31e-06 | 0.00 ± 0.00 | 0.00 ± 0.00 | 0.98 ± 0.02 | 0.88 ± 0.12 |
| Histogram Min | 0.00e+00 ± 0.00e+00 | 0.00 ± 0.00 | 0.00 ± 0.00 | 1.00 ± 0.00 | 1.00 ± 0.00 |
| Histogram Range | 6.49e-02 ± 2.11e-02 | 6.09 ± 5.90 | 21.16 ± 12.90 | 0.96 ± 0.02 | 0.65 ± 0.19 |
| Histogram RMS | 1.75e-02 ± 2.06e-03 | 2.84 ± 2.55 | 8.13 ± 7.56 | 0.95 ± 0.02 | 0.62 ± 0.21 |
| Histogram Skewness | 2.19e+00 ± 7.20e-01 | 5.40 ± 5.69 | 19.62 ± 13.32 | 0.98 ± 0.01 | 0.63 ± 0.39 |
| Histogram STD | 1.57e-02 ± 2.32e-03 | 3.51 ± 3.01 | 10.10 ± 9.13 | 0.95 ± 0.02 | 0.63 ± 0.22 |
| Histogram Uniformity | 3.97e-02 ± 9.31e-03 | 5.73 ± 5.29 | 15.93 ± 15.20 | 0.94 ± 0.03 | 0.60 ± 0.16 |
| Histogram Variance | 2.51e-04 ± 7.33e-05 | 7.07 ± 6.28 | 19.60 ± 18.10 | 0.94 ± 0.03 | 0.60 ± 0.16 |
| Histogram TotalFrequency | 4.90e+04 ± 3.69e+04 | 18.07 ± 7.40 | 83.35 ± 11.32 | 0.95 ± 0.04 | 0.43 ± 0.27 |
| Signal Quantile 0.01 | 3.62e+02 ± 1.63e+02 | 38.98 ± 25.85 | 76.95 ± 50.45 | 0.67 ± 0.05 | 0.28 ± 0.31 |
| Signal Quantile 0.1 | 8.35e+02 ± 1.96e+02 | 6.73 ± 6.35 | 37.41 ± 29.96 | 0.93 ± 0.06 | 0.46 ± 0.57 |
| Signal Quantile 0.2 | 1.01e+03 ± 2.39e+02 | 3.85 ± 3.30 | 28.40 ± 22.66 | 0.97 ± 0.02 | 0.50 ± 0.49 |
| Signal Quantile 0.3 | 1.15e+03 ± 2.82e+02 | 3.52 ± 3.08 | 22.96 ± 17.41 | 0.97 ± 0.00 | 0.54 ± 0.36 |
| Signal Quantile 0.4 | 1.30e+03 ± 3.39e+02 | 3.77 ± 3.96 | 19.14 ± 12.59 | 0.96 ± 0.01 | 0.63 ± 0.33 |
| Signal Quantile 0.5 | 1.46e+03 ± 4.05e+02 | 3.28 ± 3.38 | 16.18 ± 10.60 | 0.98 ± 0.00 | 0.68 ± 0.33 |
| Signal Quantile 0.6 | 1.61e+03 ± 4.39e+02 | 2.86 ± 2.71 | 14.24 ± 9.52 | 0.98 ± 0.00 | 0.72 ± 0.28 |
| Signal Quantile 0.7 | 1.76e+03 ± 4.58e+02 | 2.30 ± 1.73 | 12.40 ± 9.84 | 0.99 ± 0.00 | 0.77 ± 0.17 |
| Signal Quantile 0.8 | 1.94e+03 ± 4.47e+02 | 1.95 ± 1.69 | 9.84 ± 7.97 | 0.99 ± 0.00 | 0.85 ± 0.04 |
| Signal Quantile 0.9 | 2.19e+03 ± 4.10e+02 | 1.90 ± 1.86 | 7.50 ± 5.15 | 0.99 ± 0.00 | 0.91 ± 0.04 |
| Signal Quantile 0.99 | 2.71e+03 ± 3.26e+02 | 1.28 ± 1.79 | 5.08 ± 6.34 | 0.99 ± 0.01 | 0.87 ± 0.01 |

**Table 55** Grey Level Co-occurrence Matrix (GLCM) features for the patients of the Soft-Tissues Sarcomas (STS) dataset. In the table, the features computed for 16-bins histogram discretization are presented. The first column shows the mean and standard deviations of the original radiomic features computed on all the patients. The mean and standard deviation of both percentage difference and Intra-class Correlation Coefficient (ICC) are shown for both minimum and maximum entity scaling. Values are shown with scientific format only for the original features. The numbers are approximated to the second decimal significant digit.

| **GREY LEVEL CO-OCCURRENCE MATRIX (16-BINS DISCRETIZATION)** | | | | | |
| --- | --- | --- | --- | --- | --- |
| **Feature name** | **Original value** | **Diff %**  **(min. entity)** | **Diff %**  **(max. entity)** | **ICC_SMALL_** | **ICC_LARGE_** |
| Autocorrelation | 3.49e+01 ± 1.71e+01 | 4.83 ± 3.02 | 23.01 ± 12.52 | 0.99 ± 0.00 | 0.79 ± 0.18 |
| Cluster Prominence | 3.38e+04 ± 3.17e+04 | 8.11 ± 5.75 | 34.74 ± 20.76 | 0.99 ± 0.00 | 0.81 ± 0.16 |
| Cluster Shade | 2.08e+03 ± 1.49e+03 | 6.51 ± 4.49 | 29.17 ± 16.08 | 0.99 ± 0.00 | 0.81 ± 0.17 |
| Cluster Tendency | 1.41e+02 ± 6.84e+01 | 4.69 ± 3.07 | 22.59 ± 12.23 | 0.99 ± 0.00 | 0.79 ± 0.18 |
| Contrast | 3.65e+00 ± 1.20e+00 | 7.61 ± 5.34 | 16.27 ± 17.35 | 0.97 ± 0.01 | 0.79 ± 0.12 |
| Correlation | 6.98e+03 ± 6.12e+03 | 6.08 ± 6.46 | 28.23 ± 24.17 | 0.99 ± 0.01 | 0.85 ± 0.13 |
| Difference Entropy | 2.17e+00 ± 1.76e-01 | 2.17 ± 1.78 | 4.50 ± 4.63 | 0.95 ± 0.03 | 0.74 ± 0.07 |
| Dissimilarity | 1.34e+00 ± 2.20e-01 | 3.99 ± 3.04 | 8.24 ± 7.75 | 0.96 ± 0.02 | 0.80 ± 0.07 |
| Energy | 3.79e-02 ± 1.71e-02 | 8.58 ± 7.10 | 23.17 ± 20.37 | 0.94 ± 0.03 | 0.61 ± 0.15 |
| Entropy | 5.54e+00 ± 4.66e-01 | 2.21 ± 1.85 | 5.48 ± 6.13 | 0.95 ± 0.01 | 0.68 ± 0.17 |
| Homogeneity | 5.69e-01 ± 3.69e-02 | 1.60 ± 1.08 | 3.06 ± 2.81 | 0.96 ± 0.02 | 0.81 ± 0.05 |
| Homogeneity2 | 5.23e-01 ± 4.53e-02 | 2.14 ± 1.43 | 4.06 ± 3.66 | 0.96 ± 0.02 | 0.81 ± 0.05 |
| IMOC1 | -1.16e-01 ± 4.84e-02 | 7.12 ± 6.67 | 34.10 ± 43.63 | 0.98 ± 0.01 | 0.73 ± 0.20 |
| IMOC2 | 6.78e-01 ± 1.23e-01 | 2.85 ± 2.89 | 12.99 ± 15.52 | 0.98 ± 0.01 | 0.66 ± 0.23 |
| Inertia | 1.00e+00 ± 1.83e-05 | 0.00 ± 0.00 | 0.00 ± 0.00 | 0.97 ± 0.01 | 0.79 ± 0.12 |
| Inverse Difference moment | 9.95e-01 ± 8.44e-04 | 0.02 ± 0.01 | 0.04 ± 0.04 | 0.96 ± 0.02 | 0.80 ± 0.07 |
| Inverse Difference moment2 | 4.38e-01 ± 2.67e-02 | 1.15 ± 0.86 | 3.26 ± 2.45 | 0.97 ± 0.01 | 0.75 ± 0.12 |
| Inverse Variance | 9.61e-02 ± 5.22e-02 | 8.46 ± 5.89 | 30.64 ± 25.76 | 0.97 ± 0.02 | 0.71 ± 0.11 |
| Max Probability | 1.10e+01 ± 2.72e+00 | 2.58 ± 1.64 | 14.49 ± 8.07 | 0.99 ± 0.00 | 0.77 ± 0.21 |
| Sum Average | 3.76e+00 ± 3.32e-01 | 1.59 ± 1.74 | 6.06 ± 6.25 | 0.97 ± 0.00 | 0.68 ± 0.22 |
| Sum Entropy | 3.65e+00 ± 1.20e+00 | 7.61 ± 5.34 | 16.27 ± 17.35 | 0.97 ± 0.01 | 0.79 ± 0.12 |

**Table 56** Grey Level Co-occurrence Matrix (GLCM) features for the patients of the Soft-Tissues Sarcomas (STS) dataset. In the table, the features computed for 32-bins histogram discretization are presented. The first column shows the mean and standard deviations of the original radiomic features computed on all the patients. The mean and standard deviation of both percentage difference and Intra-class Correlation Coefficient (ICC) are shown for both minimum and maximum entity scaling. Values are shown with scientific format only for the original features. The numbers are approximated to the second decimal significant digit.

| **GREY LEVEL CO-OCCURRENCE MATRIX (32-BINS DISCRETIZATION)** | | | | | |
| --- | --- | --- | --- | --- | --- |
| **Feature name** | **Original value** | **Diff %**  **(min. entity)** | **Diff %**  **(max. entity)** | **ICC_SMALL_** | **ICC_LARGE_** |
| Autocorrelation | 1.51e+02 ± 7.13e+01 | 4.64 ± 2.89 | 22.28 ± 12.13 | 0.99 ± 0.00 | 0.79 ± 0.18 |
| Cluster Prominence | 6.23e+05 ± 5.66e+05 | 7.86 ± 5.49 | 33.84 ± 19.99 | 0.99 ± 0.00 | 0.81 ± 0.16 |
| Cluster Shade | 1.87e+04 ± 1.29e+04 | 6.27 ± 4.27 | 28.30 ± 15.53 | 0.99 ± 0.00 | 0.81 ± 0.17 |
| Cluster Tendency | 6.15e+02 ± 2.86e+02 | 4.49 ± 2.92 | 21.78 ± 11.87 | 0.99 ± 0.00 | 0.79 ± 0.18 |
| Contrast | 1.41e+01 ± 4.81e+00 | 7.90 ± 5.51 | 17.05 ± 18.18 | 0.97 ± 0.01 | 0.78 ± 0.12 |
| Correlation | 1.21e+05 ± 1.02e+05 | 6.16 ± 6.37 | 28.40 ± 24.66 | 0.99 ± 0.01 | 0.85 ± 0.12 |
| Difference Entropy | 3.00e+00 ± 1.97e-01 | 1.78 ± 1.47 | 3.66 ± 3.73 | 0.95 ± 0.03 | 0.74 ± 0.07 |
| Dissimilarity | 2.68e+00 ± 4.40e-01 | 3.95 ± 2.98 | 8.27 ± 7.73 | 0.96 ± 0.02 | 0.80 ± 0.08 |
| Energy | 1.04e-02 ± 4.87e-03 | 9.30 ± 7.25 | 24.85 ± 19.87 | 0.94 ± 0.03 | 0.62 ± 0.15 |
| Entropy | 7.46e+00 ± 4.81e-01 | 1.75 ± 1.40 | 4.33 ± 4.68 | 0.95 ± 0.02 | 0.67 ± 0.16 |
| Homogeneity | 4.21e-01 ± 3.37e-02 | 1.99 ± 1.25 | 4.03 ± 3.41 | 0.96 ± 0.02 | 0.80 ± 0.04 |
| Homogeneity2 | 3.45e-01 ± 3.99e-02 | 2.86 ± 1.71 | 5.75 ± 4.86 | 0.96 ± 0.02 | 0.80 ± 0.05 |
| IMOC1 | -9.54e-02 ± 3.82e-02 | 7.76 ± 7.34 | 35.67 ± 41.10 | 0.98 ± 0.01 | 0.70 ± 0.18 |
| IMOC2 | 7.03e-01 ± 1.12e-01 | 2.49 ± 2.39 | 11.52 ± 12.66 | 0.98 ± 0.01 | 0.65 ± 0.24 |
| Inertia | 1.00e+00 ± 4.59e-06 | 0.00 ± 0.00 | 0.00 ± 0.00 | 0.96 ± 0.01 | 0.79 ± 0.11 |
| Inverse Difference moment | 9.97e-01 ± 4.25e-04 | 0.01 ± 0.01 | 0.02 ± 0.02 | 0.96 ± 0.02 | 0.80 ± 0.08 |
| Inverse Difference moment2 | 3.34e-01 ± 3.22e-02 | 2.44 ± 1.68 | 4.42 ± 3.68 | 0.95 ± 0.03 | 0.83 ± 0.05 |
| Inverse Variance | 3.15e-02 ± 1.80e-02 | 8.17 ± 5.63 | 32.83 ± 24.09 | 0.98 ± 0.01 | 0.74 ± 0.10 |
| Max Probability | 2.30e+01 ± 5.44e+00 | 2.47 ± 1.57 | 13.86 ± 7.76 | 0.99 ± 0.00 | 0.77 ± 0.21 |
| Sum Average | 4.74e+00 ± 3.36e-01 | 1.29 ± 1.39 | 4.96 ± 5.00 | 0.97 ± 0.00 | 0.67 ± 0.23 |
| Sum Entropy | 1.41e+01 ± 4.81e+00 | 7.90 ± 5.51 | 17.05 ± 18.18 | 0.97 ± 0.01 | 0.78 ± 0.12 |

**Table 57** Grey Level Co-occurrence Matrix (GLCM) features for the patients of the Soft-Tissues Sarcomas (STS) dataset. In the table, the features computed for 64-bins histogram discretization are presented. The first column shows the mean and standard deviations of the original radiomic features computed on all the patients. The mean and standard deviation of both percentage difference and Intra-class Correlation Coefficient (ICC) are shown for both minimum and maximum entity scaling. Values are shown with scientific format only for the original features. The numbers are approximated to the second decimal significant digit.

| **GREY LEVEL CO-OCCURRENCE MATRIX (64-BINS DISCRETIZATION)** | | | | | |
| --- | --- | --- | --- | --- | --- |
| **Feature name** | **Original value** | **Diff %**  **(min. entity)** | **Diff %**  **(max. entity)** | **ICC_SMALL_** | **ICC_LARGE_** |
| Autocorrelation | 6.27e+02 ± 2.90e+02 | 4.56 ± 2.83 | 21.93 ± 11.94 | 0.99 ± 0.00 | 0.79 ± 0.18 |
| Cluster Prominence | 1.06e+07 ± 9.50e+06 | 7.74 ± 5.39 | 33.42 ± 19.63 | 0.99 ± 0.00 | 0.81 ± 0.16 |
| Cluster Shade | 1.58e+05 ± 1.07e+05 | 6.16 ± 4.18 | 27.90 ± 15.29 | 0.99 ± 0.00 | 0.80 ± 0.17 |
| Cluster Tendency | 2.56e+03 ± 1.17e+03 | 4.40 ± 2.86 | 21.42 ± 11.70 | 0.99 ± 0.00 | 0.79 ± 0.18 |
| Contrast | 5.61e+01 ± 1.92e+01 | 8.00 ± 5.58 | 17.38 ± 18.52 | 0.97 ± 0.01 | 0.78 ± 0.12 |
| Correlation | 2.01e+06 ± 1.67e+06 | 6.20 ± 6.32 | 28.32 ± 24.45 | 0.99 ± 0.01 | 0.86 ± 0.11 |
| Difference Entropy | 3.92e+00 ± 2.08e-01 | 1.46 ± 1.20 | 3.00 ± 3.03 | 0.95 ± 0.03 | 0.74 ± 0.06 |
| Dissimilarity | 5.36e+00 ± 8.79e-01 | 3.97 ± 2.99 | 8.35 ± 7.80 | 0.96 ± 0.02 | 0.80 ± 0.08 |
| Energy | 2.72e-03 ± 1.29e-03 | 9.75 ± 7.35 | 26.34 ± 20.84 | 0.94 ± 0.04 | 0.61 ± 0.15 |
| Entropy | 9.39e+00 ± 4.94e-01 | 1.46 ± 1.13 | 4.08 ± 3.54 | 0.95 ± 0.02 | 0.66 ± 0.13 |
| Homogeneity | 2.92e-01 ± 2.76e-02 | 2.36 ± 1.45 | 4.90 ± 4.07 | 0.96 ± 0.02 | 0.79 ± 0.04 |
| Homogeneity2 | 2.05e-01 ± 2.85e-02 | 3.44 ± 1.97 | 7.32 ± 5.88 | 0.96 ± 0.02 | 0.79 ± 0.05 |
| IMOC1 | -8.71e-02 ± 2.84e-02 | 7.27 ± 6.75 | 38.95 ± 36.87 | 0.97 ± 0.02 | 0.49 ± 0.03 |
| IMOC2 | 7.42e-01 ± 9.08e-02 | 2.10 ± 1.95 | 10.76 ± 9.54 | 0.98 ± 0.02 | 0.54 ± 0.11 |
| Inertia | 1.00e+00 ± 1.18e-06 | 0.00 ± 0.00 | 0.00 ± 0.00 | 0.93 ± 0.02 | 0.64 ± 0.24 |
| Inverse Difference moment | 9.99e-01 ± 2.14e-04 | 0.00 ± 0.00 | 0.01 ± 0.01 | 0.96 ± 0.02 | 0.80 ± 0.08 |
| Inverse Difference moment2 | 2.09e-01 ± 2.79e-02 | 3.42 ± 1.94 | 7.02 ± 5.43 | 0.96 ± 0.02 | 0.79 ± 0.05 |
| Inverse Variance | 9.18e-03 ± 5.00e-03 | 9.65 ± 5.70 | 37.22 ± 26.45 | 0.97 ± 0.02 | 0.68 ± 0.07 |
| Max Probability | 4.70e+01 ± 1.09e+01 | 2.42 ± 1.54 | 13.56 ± 7.61 | 0.99 ± 0.00 | 0.77 ± 0.21 |
| Sum Average | 5.74e+00 ± 3.37e-01 | 1.08 ± 1.14 | 4.15 ± 4.11 | 0.97 ± 0.01 | 0.67 ± 0.23 |
| Sum Entropy | 5.61e+01 ± 1.92e+01 | 8.00 ± 5.58 | 17.38 ± 18.52 | 0.97 ± 0.01 | 0.78 ± 0.12 |

**Table 58** Grey Level Run Length Matrix (GLRLM) features for the patients of the Soft-Tissues Sarcomas (STS) dataset. In the table, the features computed for 16-bins histogram discretization are presented. The first column shows the mean and standard deviations of the original radiomic features computed on all the patients. The mean and standard deviation of both percentage difference and Intra-class Correlation Coefficient (ICC) are shown for both minimal and maximal entity scaling. Values are shown with scientific format only for the original features. The numbers are approximated to the second decimal significant digit.

| **GREY LEVEL RUN LENGTH MATRIX (16-BINS DISCRETIZATION)** | | | | | |
| --- | --- | --- | --- | --- | --- |
| **Feature name** | **Original value** | **Diff %**  **(min. entity)** | **Diff %**  **(max. entity)** | **ICC_SMALL_** | **ICC_LARGE_** |
| Short Run Emphasis | 9.90e-01 ± 7.22e-03 | 0.11 ± 0.15 | 0.31 ± 0.43 | 0.97 ± 0.02 | 0.83 ± 0.04 |
| Long Run Emphasis | 1.04e+00 ± 3.57e-02 | 0.47 ± 0.67 | 1.37 ± 2.06 | 0.97 ± 0.02 | 0.81 ± 0.03 |
| Grey Level Non-Uniformity | 4.82e+03 ± 3.52e+03 | 15.17 ± 8.59 | 71.38 ± 19.88 | 0.95 ± 0.05 | 0.46 ± 0.35 |
| Run Length Non-Uniformity | 3.49e+04 ± 2.49e+04 | 19.46 ± 7.44 | 86.76 ± 15.14 | 0.93 ± 0.05 | 0.39 ± 0.25 |
| Run Percentage | 7.47e-01 ± 3.85e-02 | 1.24 ± 0.87 | 2.98 ± 2.27 | 0.96 ± 0.01 | 0.79 ± 0.10 |
| Low Grey Level Run Emphasis | 5.00e-02 ± 2.20e-02 | 20.13 ± 11.57 | 131.88 ± 127.57 | 0.88 ± 0.06 | 0.46 ± 0.46 |
| high Grey Level Run Emphasis | 4.75e+01 ± 1.66e+01 | 5.18 ± 2.79 | 21.89 ± 11.63 | 0.98 ± 0.00 | 0.72 ± 0.26 |
| Short Run Low Grey Level Emphasis | 4.96e-02 ± 2.16e-02 | 20.28 ± 11.52 | 132.11 ± 127.49 | 0.88 ± 0.06 | 0.45 ± 0.46 |
| Short Run High Grey Level Emphasis | 4.70e+01 ± 1.61e+01 | 5.14 ± 2.73 | 21.78 ± 11.53 | 0.98 ± 0.00 | 0.72 ± 0.26 |
| Long Run Low Grey Level Emphasis | 5.20e-02 ± 2.38e-02 | 19.47 ± 11.77 | 130.78 ± 127.95 | 0.89 ± 0.06 | 0.48 ± 0.47 |
| Long Run High Grey Level Emphasis | 4.97e+01 ± 1.93e+01 | 5.36 ± 3.06 | 22.36 ± 12.36 | 0.98 ± 0.00 | 0.72 ± 0.23 |

**Table 59** Grey Level Run Length Matrix (GLRLM) features for the patients of the Soft-Tissues Sarcomas (STS) dataset. In the table, the features computed for 32-bins histogram discretization are presented. The first column shows the mean and standard deviations of the original radiomic features computed on all the patients. The mean and standard deviation of both percentage difference and Intra-class Correlation Coefficient (ICC) are shown for both minimal and maximal entity scaling. Values are shown with scientific format only for the original features. The numbers are approximated to the second decimal significant digit.

| **GREY LEVEL RUN LENGTH MATRIX (32-BINS DISCRETIZATION)** | | | | | |
| --- | --- | --- | --- | --- | --- |
| **Feature name** | **Original value** | **Diff %**  **(min. entity)** | **Diff %**  **(max. entity)** | **ICC_SMALL_** | **ICC_LARGE_** |
| Short Run Emphasis | 9.82e-01 ± 7.84e-03 | 0.16 ± 0.13 | 0.39 ± 0.37 | 0.97 ± 0.02 | 0.82 ± 0.03 |
| Long Run Emphasis | 1.08e+00 ± 3.75e-02 | 0.65 ± 0.56 | 1.67 ± 1.53 | 0.97 ± 0.02 | 0.82 ± 0.01 |
| Grey Level Non-Uniformity | 3.01e+03 ± 2.23e+03 | 14.09 ± 8.72 | 67.54 ± 21.21 | 0.96 ± 0.04 | 0.49 ± 0.36 |
| Run Length Non-Uniformity | 3.97e+04 ± 2.85e+04 | 18.98 ± 7.41 | 85.74 ± 13.87 | 0.94 ± 0.05 | 0.40 ± 0.26 |
| Run Percentage | 8.64e-01 ± 2.28e-02 | 0.61 ± 0.40 | 1.74 ± 1.10 | 0.97 ± 0.00 | 0.76 ± 0.08 |
| Low Grey Level Run Emphasis | 1.63e-02 ± 8.58e-03 | 29.14 ± 18.29 | 228.41 ± 238.44 | 0.82 ± 0.08 | 0.39 ± 0.42 |
| High Grey Level Run Emphasis | 1.78e+02 ± 6.79e+01 | 5.10 ± 2.83 | 22.57 ± 11.88 | 0.98 ± 0.00 | 0.73 ± 0.24 |
| Short Run Low Grey Level Emphasis | 1.61e-02 ± 8.45e-03 | 29.43 ± 18.21 | 228.33 ± 237.05 | 0.81 ± 0.08 | 0.38 ± 0.41 |
| Short Run High Grey Level Emphasis | 1.74e+02 ± 6.56e+01 | 5.06 ± 2.78 | 22.54 ± 11.77 | 0.98 ± 0.00 | 0.73 ± 0.24 |
| Long Run Low Grey Level Emphasis | 1.74e-02 ± 9.30e-03 | 28.00 ± 18.53 | 228.71 ± 244.72 | 0.83 ± 0.09 | 0.40 ± 0.43 |
| Long Run High Grey Level Emphasis | 1.92e+02 ± 7.95e+01 | 5.28 ± 3.08 | 22.72 ± 12.36 | 0.98 ± 0.00 | 0.74 ± 0.23 |

**Table 60** Grey Level Run Length Matrix (GLRLM) features for the patients of the Soft-Tissues Sarcomas (STS) dataset. In the table, the features computed for 64-bins histogram discretization are presented. The first column shows the mean and standard deviations of the original radiomic features computed on all the patients. The mean and standard deviation of both percentage difference and Intra-class Correlation Coefficient (ICC) are shown for both minimal and maximal entity scaling. Values are shown with scientific format only for the original features. The numbers are approximated to the second decimal significant digit. Features for which mean ICC_SMALL_ is less than 0.78 (unstable features) are highlighted in red.

| **GREY LEVEL RUN LENGTH MATRIX (64-BINS DISCRETIZATION)** | | | | | |
| --- | --- | --- | --- | --- | --- |
| **Feature name** | **Original value** | **Diff %**  **(min. entity)** | **Diff %**  **(max. entity)** | **ICC_SMALL_** | **ICC_LARGE_** |
| Short Run Emphasis | 9.48e-01 ± 1.91e-02 | 0.27 ± 0.21 | 0.60 ± 0.53 | 0.99 ± 0.01 | 0.93 ± 0.01 |
| Long Run Emphasis | 1.24e+00 ± 9.39e-02 | 1.08 ± 0.84 | 2.39 ± 2.12 | 0.98 ± 0.01 | 0.91 ± 0.01 |
| Grey Level Non-Uniformity | 1.69e+03 ± 1.26e+03 | 13.78 ± 8.61 | 65.82 ± 22.19 | 0.96 ± 0.04 | 0.51 ± 0.37 |
| Run Length Non-Uniformity | 3.91e+04 ± 2.80e+04 | 18.92 ± 7.47 | 85.60 ± 13.54 | 0.94 ± 0.05 | 0.40 ± 0.26 |
| Run Percentage | 9.30e-01 ± 1.22e-02 | 0.30 ± 0.22 | 0.86 ± 0.59 | 0.96 ± 0.00 | 0.75 ± 0.08 |
| Low Grey Level Run Emphasis | 5.49e-03 ± 3.55e-03 | 40.87 ± 26.79 | 362.11 ± 398.69 | 0.76 ± 0.08 | 0.32 ± 0.34 |
| high Grey Level Run Emphasis | 6.86e+02 ± 2.75e+02 | 5.05 ± 2.89 | 22.93 ± 12.06 | 0.98 ± 0.00 | 0.74 ± 0.23 |
| Short Run Low Grey Level Emphasis | 5.28e-03 ± 3.43e-03 | 41.16 ± 26.46 | 359.06 ± 390.51 | 0.76 ± 0.07 | 0.31 ± 0.33 |
| Short Run High Grey Level Emphasis | 6.51e+02 ± 2.55e+02 | 5.00 ± 2.85 | 22.80 ± 11.93 | 0.98 ± 0.00 | 0.74 ± 0.23 |
| Long Run Low Grey Level Emphasis | 6.50e-03 ± 4.12e-03 | 39.48 ± 28.44 | 373.23 ± 434.02 | 0.77 ± 0.11 | 0.34 ± 0.38 |
| Long Run High Grey Level Emphasis | 8.48e+02 ± 3.86e+02 | 5.34 ± 3.12 | 23.38 ± 12.74 | 0.98 ± 0.00 | 0.76 ± 0.21 |
